# Genetic dissection of DNA damage tolerance in *Bacillus subtilis*: RecA and recombination functions regulate translesion synthesis

**DOI:** 10.1093/nar/gkag673

**Published:** 2026-07-06

**Authors:** Rubén Torres, Juan C Alonso

**Affiliations:** Department of Microbial Biotechnology, Centro Nacional de Biotecnología (CNB-CSIC), 3 Darwin Str, Madrid 28049, Spain; Department of Microbial Biotechnology, Centro Nacional de Biotecnología (CNB-CSIC), 3 Darwin Str, Madrid 28049, Spain

## Abstract

Mutagenesis is a fundamental, yet poorly understood, source of genetic variation that underpins microbial evolution and adaptation. When the *Bacillus subtilis* replicative DNA polymerase (DNAP) PolC encounters DNA lesions induced by endogenous or exogenous insults, it stalls and disassembles. If error-free DNA damage tolerance (DDT) sub-pathways fail to circumvent the lesion, bipartite translesion synthesis (TLS) DNAPs (PolY1 or PolY2 together with PolA) may bypass the damage to resume DNA synthesis. Mismatch repair subsequently removes misincorporated nucleotides. Here, we investigate which proteins loaded at stalled replication forks influence mutation dynamics mediated by TLS DNAPs. We demonstrate that Δ*recA*, Δ*polA*, or Δ*polY1* Δ*polY2* mutations strongly reduce cell survival following DNA damage and mutagenesis. The accessory proteins DisA, RarA, RecD2, DinG, and Mfd, which physically interact with PolA and/or RecA, differentially affect cell survival after DNA damage in the absence of TLS DNAPs and differentially modulate mutagenesis by regulating the activity of distinct TLS DNAPs, highlighting their roles in error-prone DDT. We also reveal that SOS-independent mutagenesis operates in the Δ*polA* Δ*recA* background. Elucidating the regulatory network underlying TLS provides a framework to understand bacterial speciation and may uncover new avenues to limit antibiotic resistance emergence.

## Graphical Abstract

**Graphical Abstract:**
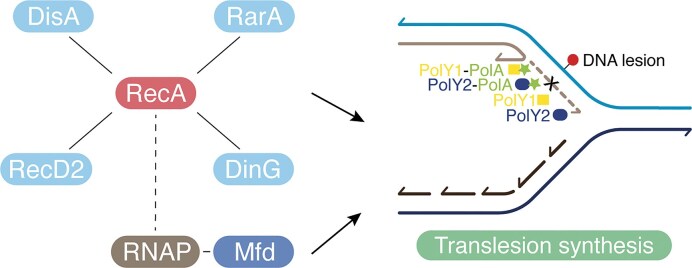

## Introduction

In bacteria, mutations arise from errors during DNA replication, endogenous processes, environmental damage, and horizontal gene transfer. When not lethal, these mutations generate heritable diversity within bacterial populations, playing a critical role in bacterial evolution and in the development of antibiotic resistance [1–3].

One of the main sources of mutations is the activity of DNA polymerases (DNAPs). Unless stated otherwise, the indicated genes and gene products are of *Bacillus subtilis* origin. Those derived from other organisms are designated using an abbreviation that includes the genus and species of the respective organism; e.g. *Escherichia coli* Pol IV is referred to as Pol IV*_Eco_. Bacillus subtilis* encodes a single DNAP with proofreading activity: the essential, high-fidelity, and highly processive C-family enzyme PolC, which catalyses leading- and lagging-strand synthesis during chromosome replication [4–7]. In addition, *B. subtilis* encodes four DNAPs that lack proofreading activity: the essential C-family polymerase DnaE, and the dispensable A-family PolA (Pol I) and Y-family polymerases PolY1 [YqjH, a functional homologue of *E. coli* Pol IV (Pol IV_*Eco*_)] and PolY2 (YqjW, a functional counterpart of Pol V_*Eco*_) (reviewed in [8–11]). These five single-polypeptide DNAPs interact with the DnaN sliding clamp through a specific clamp-binding motif (CBM), albeit with differing affinities [12]. As DnaE primarily contributes to primer synthesis and its overexpression does not increase spontaneous mutation rates in wild-type (wt) cells [5, 7] (reviewed in [13, 14]), it falls outside the scope of this study.

During replication, the replisome frequently encounters unremoved lesions or collides with arrays of RNA polymerases (RNAPs), triggering a replication stress response to promote DNA integrity [15, 16]. In response to replication stress, the replisome transiently stalls and disassembles in a significant fraction of *B. subtilis* cells [17, 18]. Cells possess error-free DNA damage tolerance (DDT) sub-pathways that allow genome replication and support cell survival. If error-free DDT sub-pathways fail to circumvent the barriers, error-prone DDT sub-pathways, catalysed by translesion synthesis (TLS) DNAPs, operate to bypass the lesions, potentially resulting in mutagenesis (reviewed in [16, 19], and references therein).

In response to replication stress, the recombinase RecA, assembled at stalled replication forks [20, 21], interacts with a variety of factors enriched at these sites that contribute to error-free DDT sub-pathways, including both positive and negative RecA modulators (such as the RarA ATPase and the RecD2 5′ → 3′ DNA helicase, respectively), the DNA damage checkpoint protein DisA, or the 3′ → 5′ exo(ribo)nuclease DinG [22–28]. Furthermore, PolA and PolY1, which contribute to error-prone DDT sub-pathways, form spontaneous foci enriched at or near replication forks, through their interactions with DnaN, even in the absence of DNA damage, and directly (PolA) or indirectly (PolY1) with RecA [28–30]. If the lesion persists, the SOS response is triggered, as sustained RecA nucleoprotein filaments promote autoproteolysis of the LexA repressor, resulting in increased expression of the PolY2 enzyme [31]. To our knowledge, single-molecule live-cell studies of the PolY2 enzyme have not been reported.

PolA interacts with PolY1 and PolY2 via a specific PolA interacting domain (SID) [22]. Deletion of *polY1* or *polY2* does not significantly affect mutagenesis; however, deletion of both genes results in a reduction in mutation frequencies comparable to that observed upon deletion of *polA* [22, 28, 32]. Overproduction of PolY1 or PolY2 increases mutagenesis, whereas overproduction of PolA does not [22]. These observations have led to the proposal that PolY1–PolA and PolY2–PolA function as bipartite error-prone DNAPs that catalyse stable, yet frequently erroneous, nucleotide incorporation opposite damaged templates at stalled replication forks [22].

The balance between error-free and error-prone DDT sub-pathways at stalled replication forks is expected to influence overall mutagenesis levels. Therefore, mis-regulation of the choice between these pathways may alter mutagenesis levels and is likely to compromise fork, and ultimately genome stability [16]. However, limited information is available regarding the players and accessory factors that control pathway selection or the activity of TLS DNAPs. Genetic studies have indicated that RarA, DisA, and DinG on one hand, and RecD2, LexA, or Mfd on the other, may regulate spontaneous and damage-induced PolY1- and PolY2-dependent mutagenesis [23, 24, 27, 33]. Nevertheless, their precise roles, and whether they influence pathway choice or directly regulate the activity of TLS DNAPs, remain unclear. In this study, we used a combination of genetic assays to dissect the roles of PolA and these recombination factors in error-prone DDT sub-pathways influencing mutagenesis.

Base-pair mismatches arising from misincorporations by DNAPs that escape proofreading are recognised and removed by the mismatch repair (MMR) mechanism in a post-replicative manner [34]. In *B. subtilis*, MMR relies on a minimal set of protein activities: the mismatch-sensor MutS interacts with and recruits the MutL endonuclease [35–37]. Because cells lacking MutS and MutL display elevated frequencies of spontaneous and methyl methanesulfonate (MMS)-induced mutagenesis [36, 37], the double Δ*mutS* Δ*mutL* (Δ*mutSL*) mutant strain was used as a control of our experiments.

Investigating how evolutionarily divergent bacteria—such as *E. coli* and *B. subtilis*, which diverged >2 billion years ago—efficiently cope with different forms of replication stress will deepen our understanding of DDT. Notably, key differences exist between these organisms. First, in *E. coli*, TLS occurs primarily post-replicatively at DNA gaps left behind the replisome, and expression of the Pol II*_Eco_*, Pol IV*_Eco_*, and Pol V*_Eco_* enzymes is damage-inducible [9, 10, 38, 39]. In contrast, TLS in *B. subtilis* occurs primarily at stalled replication forks; *polA* and *polY1* are constitutively expressed, and the SOS response induces only PolY2 expression [29–31]. Second, PolA is dispensable and functions in concert with the PolY1 and PolY2 DNAPs [22]. Like Pol I*_Eco_* (PolA*_Eco_*), which contains an N-terminal 5′→3′ flap endo/exonuclease, a central 3′→5′ proofreading exonuclease, and a C-terminal polymerase domain, PolA also possesses these three domains; however, its proofreading domain is inactive [11, 40]. Finally, in *E. coli* the activated form of Pol V*_Eco_* consists of UmuD′_2_  *_Eco_*·UmuC*_Eco_*·RecA*_Eco_*·ATP [also known as the Mutasome (Mut) complex] [9, 10]. AlphaFold modelling has also predicted a Mut-like complex (PolY2·YqjX·RecA) in *B. subtilis*, in which the RecA-NT motif of PolY2 interacts with RecA, and the N-terminal arm of YqjX, a potential UmuD-like protein, interacts with PolY2, although this predicted interface is approximately twice as large as the interaction between the UmuD′_2_  *_Eco_* N-terminal arm and UmuC*_Eco_* [9, 11, 41]. However, several observations challenge this model: (i) PolY2 appears to function as a single polypeptide capable of filling small DNA gaps in the absence of additional accessory proteins [42]; (ii) inactivation of YqjX does not significantly affect spontaneous and UV-induced mutagenesis, either in the absence or upon overproduction of PolY2 [43]; and (iii) despite our efforts, we did not detect a physical interaction between PolY2 and YqjX (unpublished data) or RecA (see the ‘Results’ section). Thus, it remains unclear whether PolY2 functions as a standalone TLS DNAP or operates in concert with RecA and YqjX, or with the SPβ-encoded YolD, another potential UmuD-like protein.

## Materials and methods

### Bacterial strains and plasmids

The *B. subtilis* wt BG214 strain, a 168-derived strain lacking bacteriophage SPβ and the conjugative transposon ICE*Bs*1, and its isogenic derivatives are listed in Supplementary Table S1. Double- or triple-mutant strains were constructed by transferring the deletion cassette of one gene, replacing the gene by an antibiotic resistance gene, into an isogenic recipient strain carrying the other gene(s) deletion via SPP1 phage generalised transduction [44]. The accuracy of the double- or triple-null mutations was confirmed by polymerase chain reaction (PCR) analysis and DNA sequencing. As gene deletion cassettes were constructed in *E. coli*, no additional alterations to the genetic background, beyond the specific gene deletion, are expected.

The *E. coli* BTH101 strain was used to analyse protein–protein interaction *in vivo* using the bacterial adenylate cyclase two-hybrid assay. For this purpose, the T18 or T25 catalytic domain of the *Bordetella* adenylate cyclase gene, carried on the pUT18, pUT18C, pKT25, or pKNT25 vectors, was fused to either the 5´- or 3´-terminus of the *disA, dinG, recD2, rarA, mfd, polA, polY1, polY2, dnaN, mutS, mutL*, or *recA* genes. The pKT25-zip and pUT18C-zip plasmids were used as positive controls, as described previously [45].

### *In vivo* protein–protein interaction assays

Protein–protein interactions were analysed using the adenylate cyclase-based bacterial two-hybrid system, as described previously [33, 45]. Plasmids encoding N- or C-terminal fusions of each gene of interest to the T18 fragment of the catalytic domain of *Bordetella pertussis* adenylate cyclase were co-transformed with plasmids encoding N- or C-terminal fusions of each gene of interest to the T25 fragment into the reporter *E. coli* BTH101 strain. Empty vectors, as well as the pKT25-zip and pUT18C-zip plasmids, were co-transformed into the reporter strain as negative and positive controls, respectively. Co-transformants were spotted onto LB plates supplemented with ampicillin, kanamycin, streptomycin, 0.5 mM isopropyl β-D-1-thiogalactopyranoside (IPTG), and 10% X-gal. Plates were incubated at 25°C for 3–4 days and photographed. Each co-transformation was performed in at least triplicate, and a representative result is shown.

### Cell viability and survival studies

MMS (Merck, Darmstadt, Germany) is an S_N_2-type alkylating agent. Alkylation-induced DNA lesions are primarily removed by base excision repair and DNA alkyltransferases; however, if apurinic sites persist, they are processed by recombinational repair pathways [15, 46]. To assess cell sensitivity to chronic exposure to MMS (for 16 h), cultures were grown at 37°C with agitation to an OD_560_ of 0.4, and appropriate dilutions were plated onto freshly prepared LB agar containing increasing concentrations of MMS, as described previously [47]. Plates were incubated overnight at 37°C and colony-forming units (CFUs) were counted. To ensure consistency between experiments, a control strain with a well-characterised phenotype was included in each assay.

All experiments were performed independently at least four times. The surviving fraction was calculated as the ratio of MMS-resistant CFUs to the total number of CFUs, and data are presented as mean ± SD. Statistical significance was assessed using a two-tailed Student’s *t*-test.

### RecA protein expression

Wt, Δ*lexA, lexA*(Ind^−^), or Δ*recA* strains were grown in LB broth at 37°C with agitation to an OD_560_ = 0.4 and subsequently treated with increasing concentrations of MMS for 30 min. Aliquots (1 ml) were harvested by centrifugation, resuspended in 150 μl of phophate buffered saline (PBS), and lysed by sonication. Cellular extracts from each experimental condition were normalised to equal total protein concentrations and resolved by 10% sodium dodecyl sulphate–polyacrylamide gel electrophoresis (SDS-PAGE) alongside purified RecA protein standards (0.5–2 ng), as previously described [48]. Proteins were transferred (300 mA, 100 min, 4°C) to Immobilon-P PVDF membranes (Merck, Darmstadt, Germany) for subsequent western blot analyses. Membranes were probed with a rabbit polyclonal anti-RecA primary antibody (120 min, 22°C) [48], followed by a goat anti-rabbit IgG-horseradish peroxidase conjugated secondary antibody (60 min, 22°C) (Merck, Darmstadt, Germany). Signal detection was performed using the Clarity Western ECL Substrate (Bio-Rad, Hercules, CA, USA).

RecA-specific bands were visualised and quantified using a ChemiDoc Touch Imaging System and the ImageLab software (Bio-Rad, Hercules, CA, USA). No signal was detected in extracts from Δ*recA* strain, indicating that the polyclonal anti-RecA antibody did not exhibit cross-reactivity with unrelated proteins. Signal intensity showed a linear correlation with RecA concentration in the purified standards, allowing quantification of RecA levels in cell extracts by interpolation from the standard curve. Intracellular RecA concentration and the number of RecA molecules per cell were estimated using the total CFU count (∼1 × 10^8^ CFUs/ml) and the molecular weight of RecA (37.9 kDa). As most CFUs consisted of single cells or non-separated cell pairs, a correction factor of ∼1.6 cells/CFU was applied, as previously described [48].

### Mutagenesis assays

Cells are unable to form colonies in the presence of rifampicin (Rif) at 8 μg·ml^−1^ [49]. However, colonies carrying specific mutations in the *rpoB* gene, which encodes the essential RNAP β-subunit RpoB, display reduced affinity for Rif, resulting in a Rif-resistant (Rif^R^) phenotype. Exploiting this property, mutagenesis was analysed using Luria-Delbrück fluctuation assays to measure the frequency of mutations occurring within the Rif-binding pocket of RpoB [33].

Thirty independent cultures per strain were grown in LB broth at 37°C with shaking to an OD_560_ = 0.8. To assess spontaneous or damage-induced mutagenesis frequencies, cultures were either left untreated or exposed to 3 mM MMS for 15 min, respectively, as described previously [33]. MMS predominantly methylates nitrogen atoms in purine bases, and excision of these lesions generates apurinic sites. If base excision repair is incomplete, error-prone DDT sub-pathways can fill these sites, resulting primarily in transversions and, to a lesser extent, transitions [50]. Cells (50 ml) were harvested by centrifugation, resuspended in LB, and plated onto LB agar plates containing Rif (8 μg·ml^−1^). Total viable cell counts were determined by plating appropriate dilutions onto LB agar plates lacking antibiotic. Plates were incubated overnight (16–18 h) at 37°C, and CFUs were counted. Mutation frequencies were calculated using the mean value from 30 independent cultures per strain, expressed as the ratio of Rif^R^ colonies to total viable cells. Statistical significance was assessed using Student’s *t*-tests.

To identify mutations conferring Rif^R^, the *rpoB* gene from Rif^R^ colonies was amplified by PCR and sequenced. The resulting nucleotide sequences were compared with the wt *rpoB* sequence to determine the nature and position of the mutations. For each strain and condition, at least 10 independent Rif^R^ colonies were analysed. Statistical significance was assessed using Student’s *t*-tests.

## Results and discussion

### PolA interacts with PolY1, DnaN, MutSL, RecA, DisA, RecD2, DinG, and Mfd

**Figure 1.**
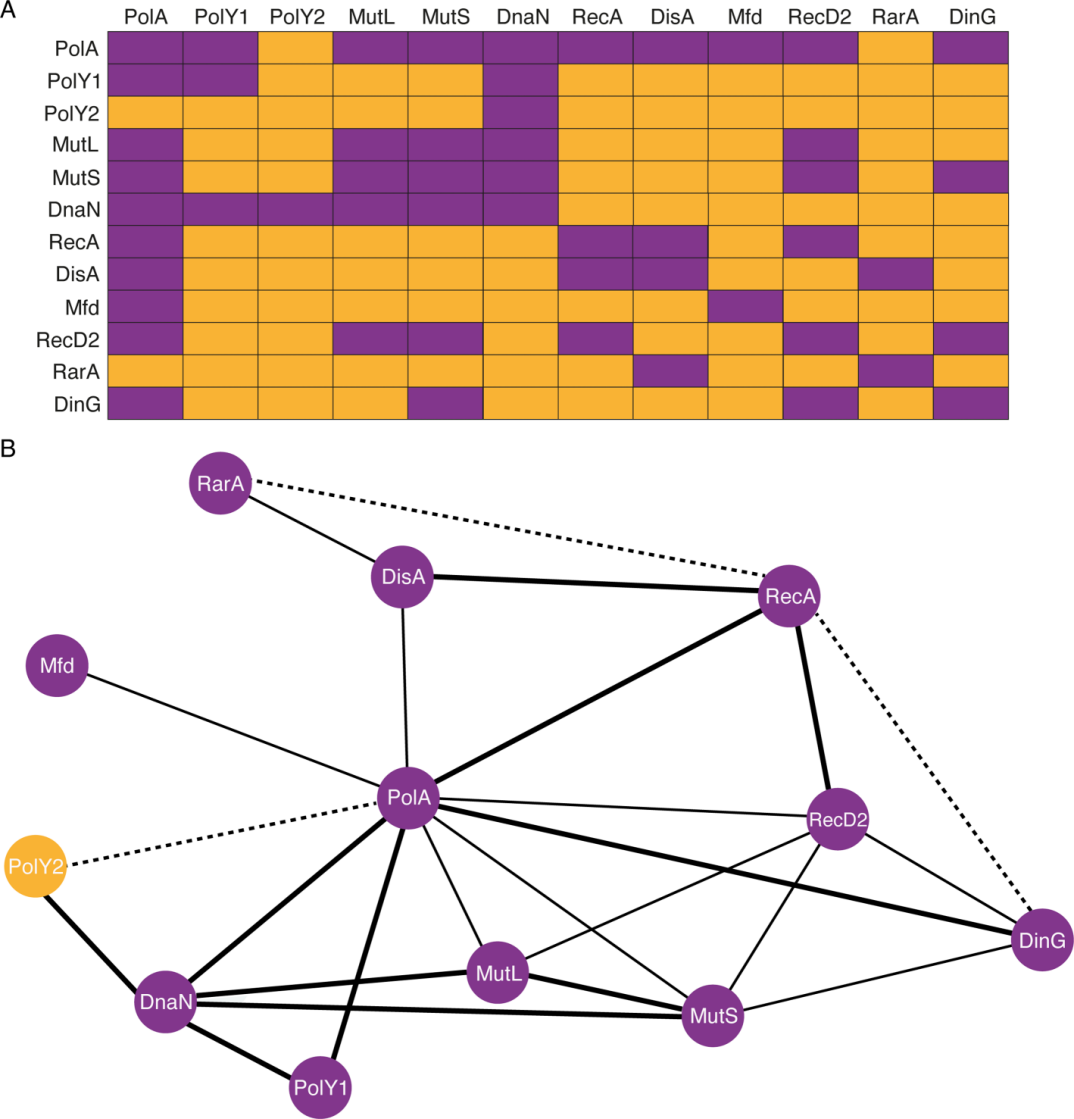
PolA interacts with PolY1, DnaN, DisA, DinG, Mfd, RecD2, MutL, MutS, and RecA. (**A**) Table summarising the results of bacterial two-hybrid interaction assays between full-length proteins. Purple colour, interaction; orange colour, no interaction. The results of each independent *in vivo* assay are provided in Supplementary Material File S2. (**B**) Schematic representation of the physical interactions previously identified and confirmed here using the bacterial two-hybrid system (thick solid lines), newly identified using the bacterial two-hybrid system (thin solid lines), and previously identified by *in vitro* biochemical assays but not confirmed here using the bacterial two-hybrid system (dashed lines). Purple circles, self-interaction; orange circles, no self-interaction.

To begin deciphering a regulatory network governing DDT pathway choice, that would impact the level of mutagenesis, we analysed physical interactions among proteins known to function during replication stress and influence mutagenesis levels (see the ‘Introduction’ section) using bacterial two-hybrid assays. We first confirmed several previously reported interactions [12, 22, 26–28, 35]: (i) RecA interacts with DisA, RecD2, and PolA; (ii) DnaN interacts with PolA, PolY1, PolY2, MutS, and MutL; (iii) PolA interacts with PolY1, RecA, and DinG; and (iv) MutS interacts with MutL (Fig. 1 and Supplementary Material File S2). However, we were unable to confirm several previously reported interactions—between RecA and RarA or DinG, or between PolA and PolY2 [22, 25, 27, 28]—, potentially due to limitations of this approach.

Notably, we report here for the first time that: (i) PolA physically interacts with DisA, Mfd, RecD2, DinG, MutS, and MutL; (ii) DinG interacts with RecD2, PolA, and MutS; (iii) RecD2 interacts with MutL and MutS; and (iv) DisA interacts with RarA and PolA (Fig. 1 and Supplementary Material File S2). Together, these interaction hubs reveal a dense network connecting proteins involved in the response to replication stress. Given that most of these proteins interact with PolA and RecA, our results suggest that these proteins may play central roles in DDT pathway choice and mutagenesis.

### Inactivation of *polA* differentially affects Δ*polY1* and Δ*polY2* cells in response to MMS

**Figure 2.**
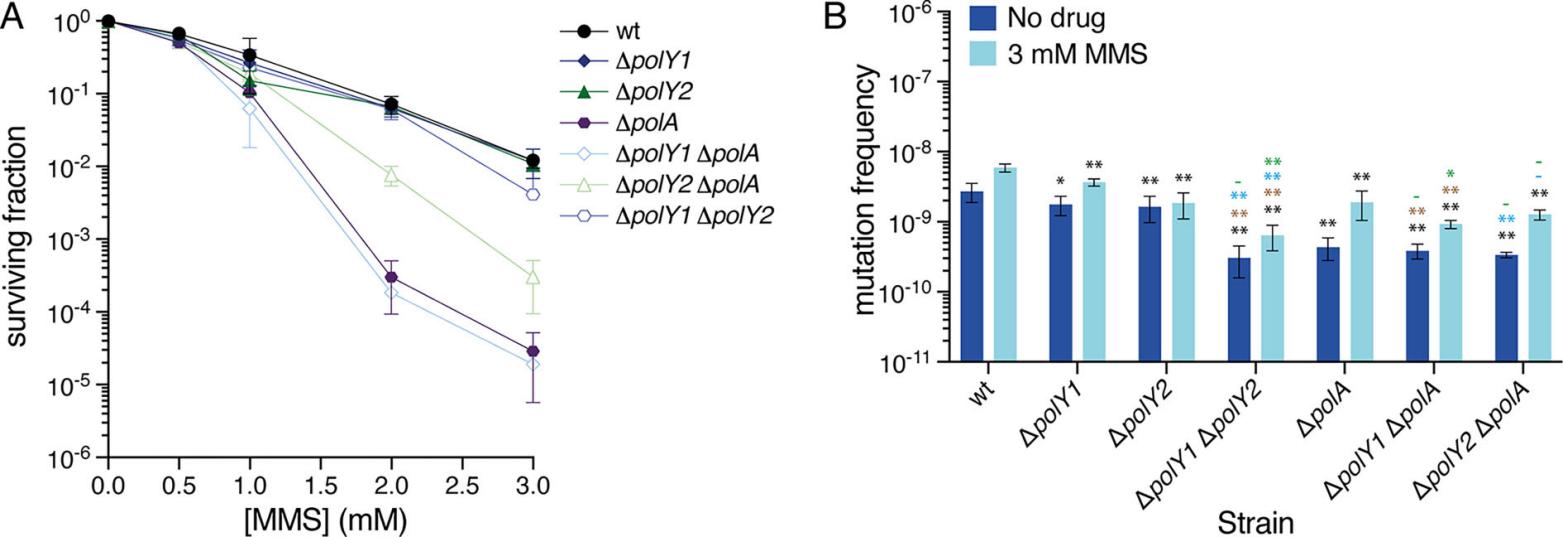
Survival following DNA damage and spontaneous and MMS-induced mutagenesis in the absence of TLS DNAPs. (**A**) The indicated strains were grown to exponential phase (OD_560_ = 0.4) in LB medium at 37°C, serially diluted, and plated on LB plates containing the indicated concentrations of MMS. Plates were incubated overnight at 37°C, and survival is plotted as CFUs after MMS exposure normalised to those obtained from untreated cells, set to 1. Data represent the mean ± SD of >3 independent experiments. Numerical data are provided in Supplementary Table S2, and *P*-values for pairwise comparisons are provided in Supplementary Table S3. (**B**) Luria–Delbrück fluctuation analyses were performed by growing the indicated strains to OD_560_ = 0.8 in LB medium. A 10-ml aliquot of each culture was exposed to 3 mM MMS for 15 min. Cultures were then plated on LB or LB supplemented with Rif, and mutation frequencies were calculated as Rif^R^ CFUs normalised to total CFUs obtained on LB from untreated cells. Data represent the mean ± SD from at least five independent experiments. Statistical significance was denoted as follows: -, *P* > 0.05; *, *P* < 0.05; **, *P* < 0.01; black colour, *P*-values relative to the wt strain; brown colour, *P*-values relative to the Δ*polY1* parental strain; blue colour, *P*-values relative to the Δ*polY2* parental strain; and green colour, *P*-values relative to the Δ*polA* parental strain. Numerical data are provided in Supplementary Table S2, and *P*-values for pairwise comparisons are provided in Supplementary Tables S4 and S5.

**Figure 3.**
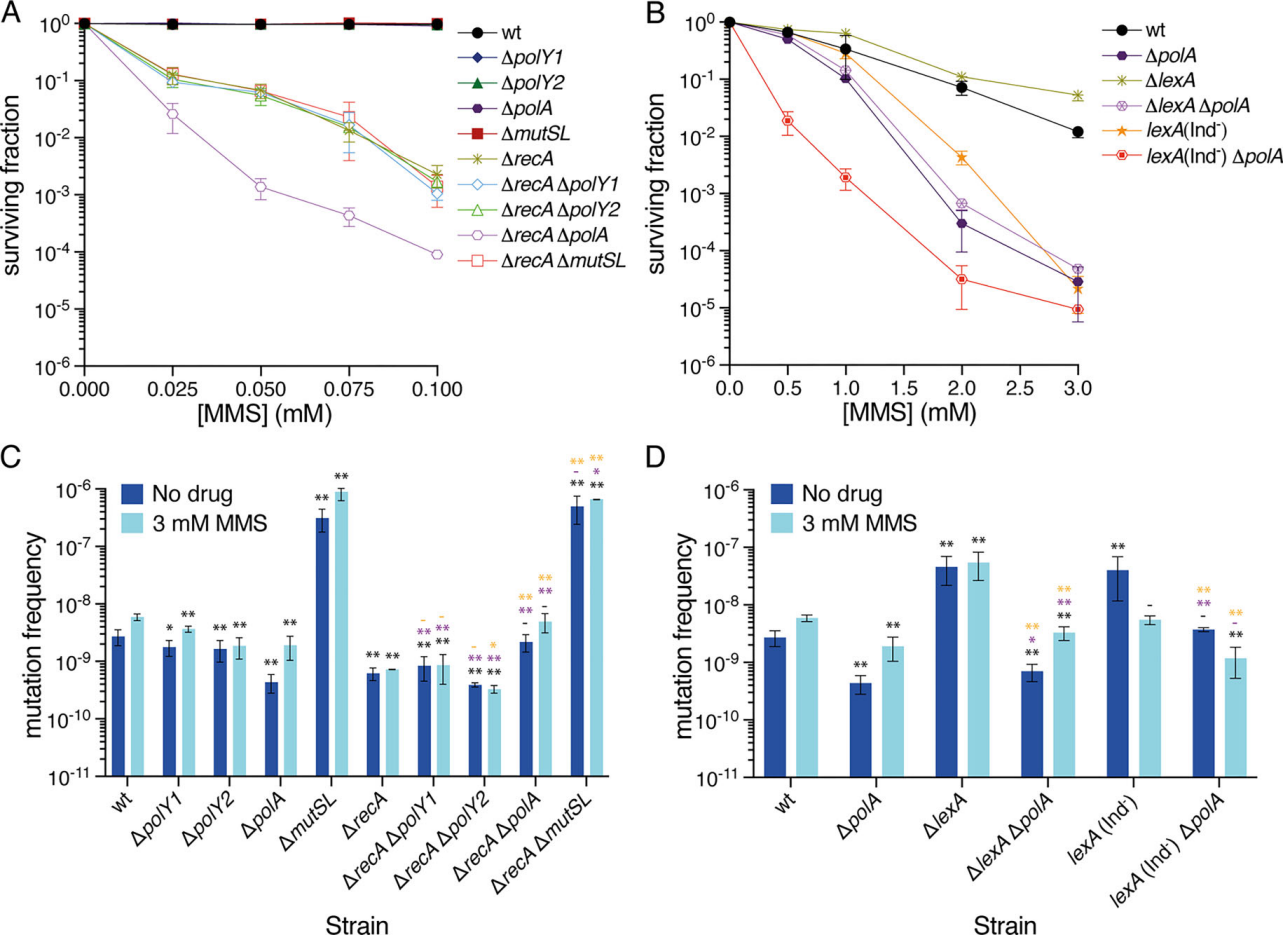
Survival following DNA damage and spontaneous and MMS-induced mutagenesis in Δ*polA* and Δ*recA* mutants in the absence of TLS DNAPs, MMR, or SOS regulation. (**A, B**) The indicated strains were grown to exponential phase (OD_560_ = 0.4) in LB medium at 37°C, serially diluted, and plated on LB plates containing the indicated concentrations of MMS. Plates were incubated overnight at 37°C, and survival is plotted as CFUs after MMS exposure normalised to those obtained from untreated cells, set to 1. Data represent the mean ± SD of >3 independent experiments. Numerical data are provided in Supplementary Table S2, and *P*-values for pairwise comparisons are provided in Supplementary Table S3. (**C, D**) Luria–Delbrück fluctuation analyses were performed by growing the indicated strains to OD_560_ = 0.8 in LB medium. A 10-ml aliquot of each culture was exposed to 3 mM MMS for 15 min. Cultures were then plated on LB or LB supplemented with Rif, and mutation frequencies were calculated as Rif^R^ CFUs normalised to total CFUs obtained on LB from untreated cells. Data represent the mean ± SD from at least five independent experiments. Statistical significance was denoted as follows: -, *P* > 0.05; *, *P* < 0.05; **, *P* < 0.01; black colour, *P*-values relative to the wt strain; purple colour, *P*-values relative to the Δ*polY1*, Δ*polY2*, Δ*polA*, or Δ*mutSL* parental strains; and orange colour, *P*-values relative to the Δ*recA*, Δ*lexA*, or *lexA*(Ind^−^) parental strains. Numerical data are provided in Supplementary Table S2, and *P*-values for pairwise comparisons are provided in Supplementary Tables S4 and S5.

**Figure 4.**
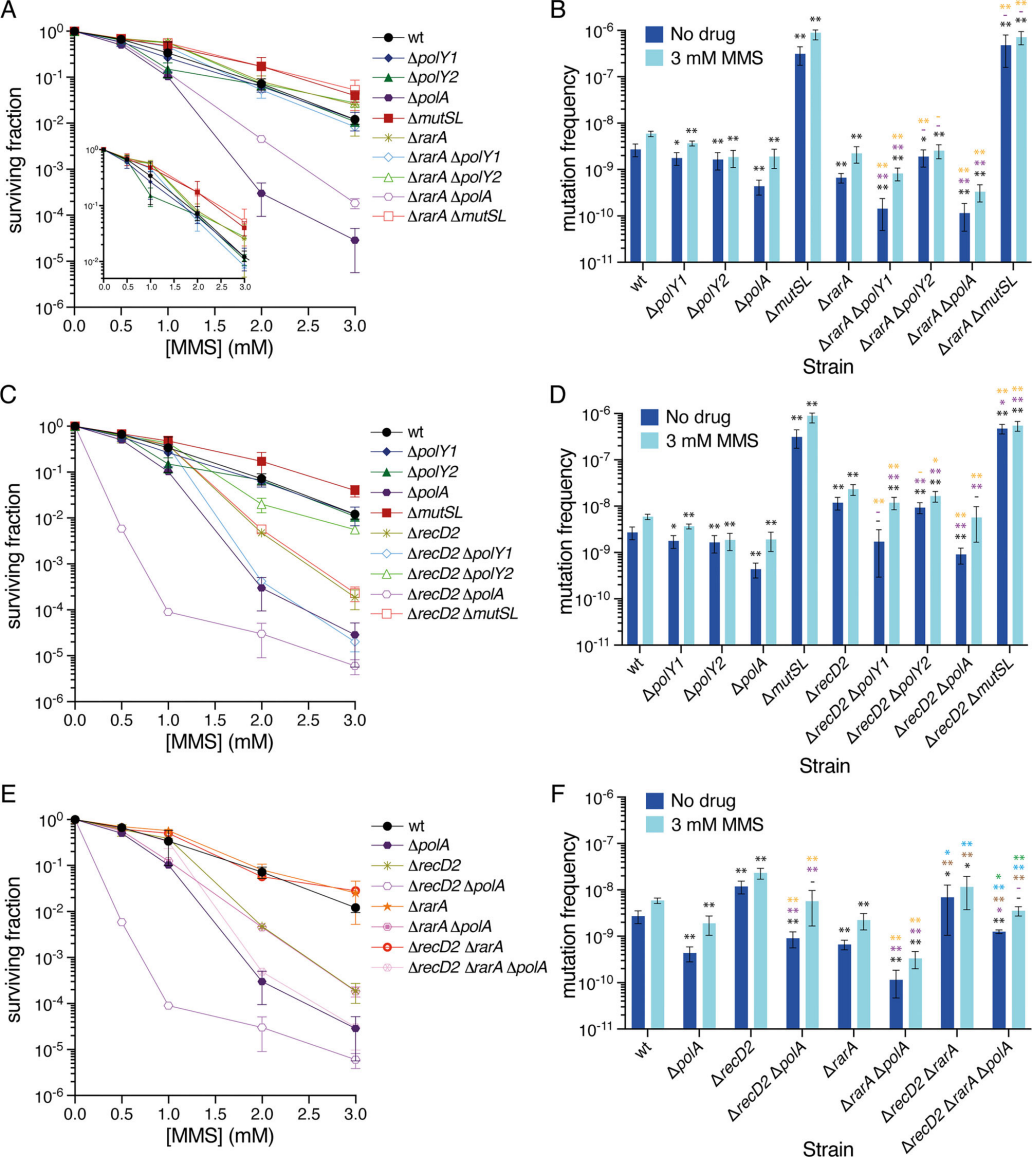
Survival following DNA damage and spontaneous and MMS-induced mutagenesis in TLS DNAPs or MMR mutants in the absence of functions modulating RecA activity. (**A, C, E**) The indicated strains were grown to exponential phase (OD_560_ = 0.4) in LB medium at 37°C, serially diluted, and plated on LB plates containing the indicated concentrations of MMS. Plates were incubated overnight at 37°C, and survival is plotted as CFUs after MMS exposure normalised to those obtained from untreated cells, set to 1. Data represent the mean ± SD of >3 independent experiments. In panel (A, inset), graphs corresponding to the less sensitive strains are shown at higher magnification. Numerical data are provided in Supplementary Table S2, and *P*-values for pairwise comparisons are provided in Supplementary Table S3. (**B, D, F**) Luria–Delbrück fluctuation analyses were performed by growing the indicated strains to OD_560_ = 0.8 in LB medium. A 10-ml aliquot of each culture was exposed to 3 mM MMS for 15 min. Cultures were then plated on LB or LB supplemented with Rif, and mutation frequencies were calculated as Rif^R^ CFUs normalised to total CFUs obtained on LB from untreated cells. Data represent the mean ± SD from at least five independent experiments. Statistical significance was denoted as follows: -, *P* > 0.05; *, *P* < 0.05; **, *P* < 0.01; black colour, *P*-values relative to the wt strain; purple colour, *P*-values relative to the Δ*polY1*, Δ*polY2*, Δ*polA*, or Δ*mutSL* parental strains; orange colour, *P*-values relative to the Δ*rarA* or Δ*recD2* parental strains; brown colour, *P*-values relative to the Δ*rarA* parental strain; blue colour, *P*-values relative to the Δ*recD2* parental strain; and green colour, *P*-values relative to the Δ*recD2* Δ*rarA* parental strain. Numerical data are provided in Supplementary Table S2, and *P*-values for pairwise comparisons are provided in Supplementary Tables S4 and S5.

**Figure 5.**
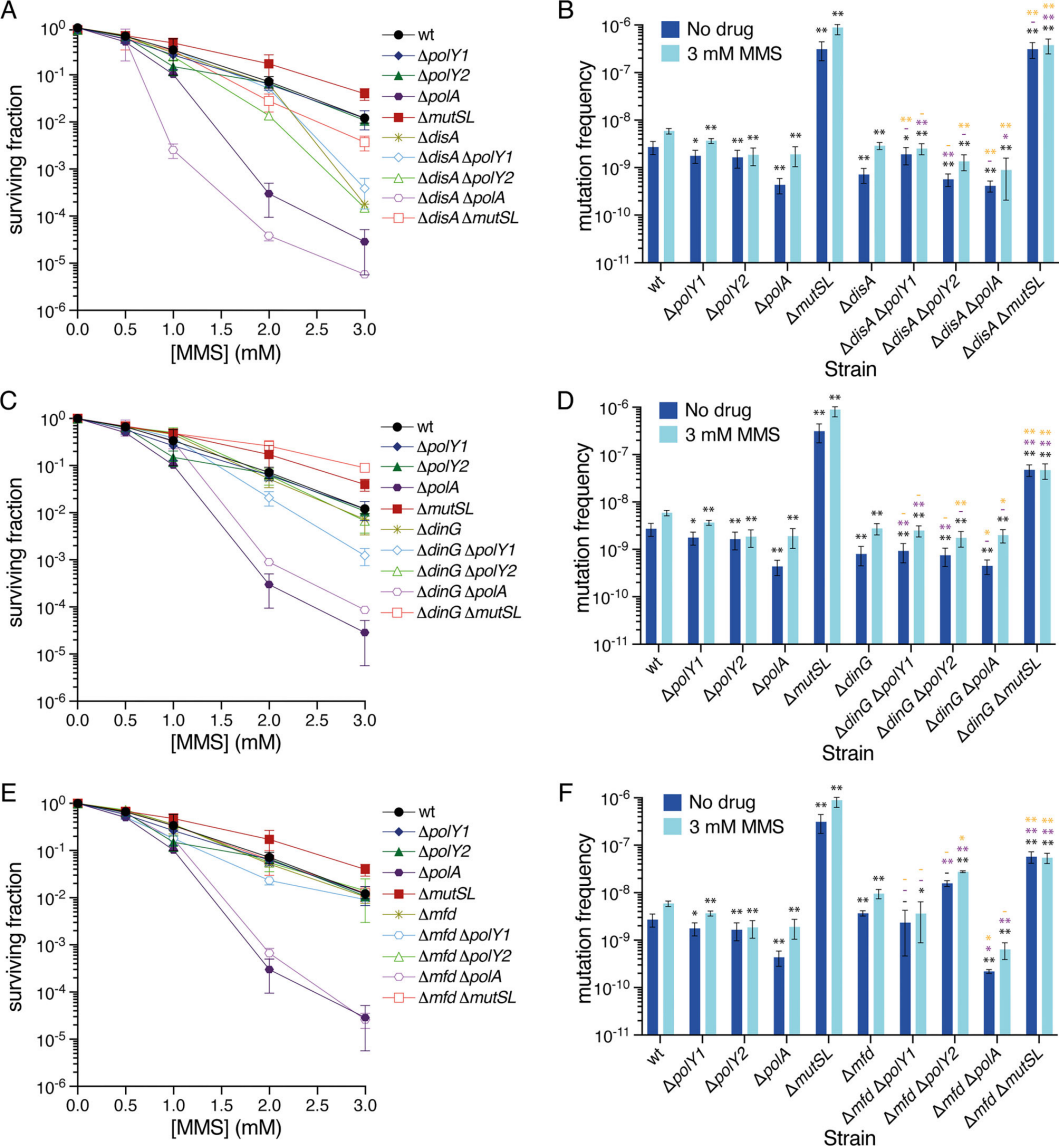
Survival following DNA damage and spontaneous and MMS-induced mutagenesis in TLS DNAPs or MMR mutants in the absence of functions regulating DDT. (**A, C, E**) The indicated strains were grown to exponential phase (OD_560_ = 0.4) in LB medium at 37°C, serially diluted, and plated on LB plates containing the indicated concentrations of MMS. Plates were incubated overnight at 37°C, and survival is plotted as CFUs after MMS exposure normalised to those obtained from untreated cells, set to 1. Data represent the mean ± SD of >3 independent experiments. Numerical data are provided in Supplementary Table S2, and *P*-values for pairwise comparisons are provided in Supplementary Table S3. (**B, D, F**) Luria–Delbrück fluctuation analyses were performed by growing the indicated strains to OD_560_ = 0.8 in LB medium. A 10-ml aliquot of each culture was exposed to 3 mM MMS for 15 min. Cultures were then plated on LB or LB supplemented with Rif, and mutation frequencies were calculated as Rif^R^ CFUs normalised to total CFUs obtained on LB from untreated cells. Data represent the mean ± SD from at least five independent experiments. Statistical significance was denoted as follows: -, *P* > 0.05; *, *P* < 0.05; **, *P* < 0.01; black colour, *P*-values relative to the wt strain; purple colour, *P*-values relative to the Δ*polY1*, Δ*polY2*, Δ*polA*, or Δ*mutSL* parental strains; and orange colour, *P*-values relative to the Δ*disA*, Δ*dinG*, or Δ*mfd* parental strains. Numerical data are provided in Supplementary Table S2, and *P*-values for pairwise comparisons are provided in Supplementary Tables S4 and S5.

Previous studies have shown that: (i) inactivation of *polY1, polY2, polA, recD2, disA, mfd*, and *recA* impairs survival of exponentially growing cells following acute exposure (15 min) to increasing concentrations of the alkylating agent MMS, albeit to varying degrees [33, 51–54]; and (ii) inactivation of *rarA* or *dinG* does not impair survival following MMS exposure, although it does impair survival in response to other DNA-damaging agents [28, 55]. These observations confirm that these proteins contribute to DNA repair. To investigate a potential interplay between TLS DNAPs and recombination functions that may influence error-prone DDT pathway choice, we constructed double-mutant strains lacking both a recombination function and a TLS DNAP in the BG214 background (Supplementary Table S1), and subjected them to chronic exposure to increasing MMS concentrations (Figs 2– 5).

**Figure 6.**
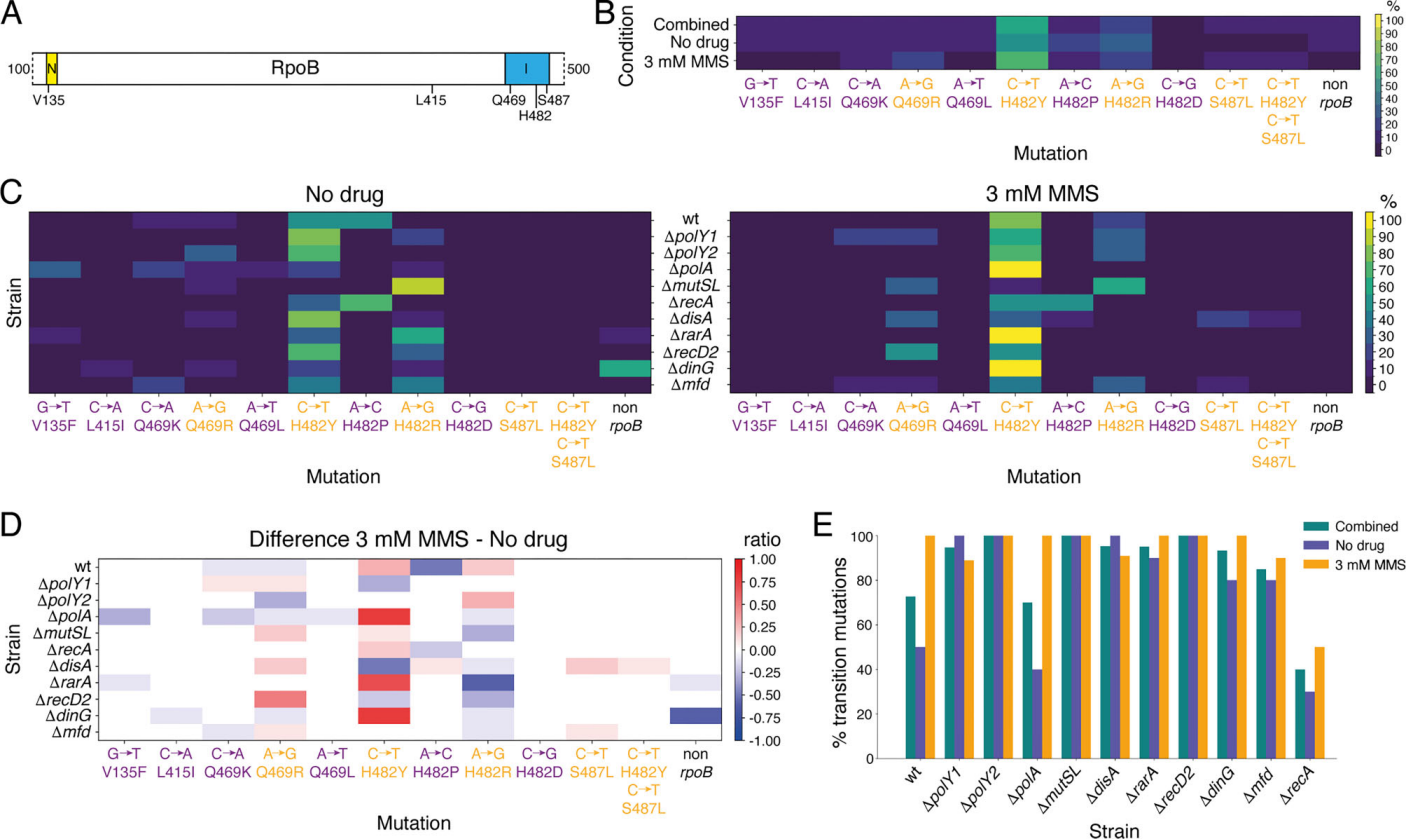
Spontaneous and MMS-induced mutational landscape in TLS DNAPs or MMR mutants, or in the absence of functions regulating DDT. (**A**) Schematic representation of the region spanning amino acids 100–500 of the *B. subtilis* RpoB protein, highlighting the mutations identified. Clusters N and I are indicated. (**B**) Heat map showing the proportions of each mutation identified across all genetic backgrounds, either combined or separated according to the absence or presence of MMS. Orange colour, transition mutation; purple colour, transversion mutation. (**C**) Heat maps showing the proportion of each mutation detected in each strain, in the absence or presence of MMS. Orange colour, transition mutation; purple colour, transversion mutation. (**D**) Heat map showing the ratio between mutations detected in each strain in the presence versus the absence of MMS. A ratio of 1 indicates that the specific mutation dominated the mutational spectrum in the presence of MMS; a ratio of −1 indicates that the mutation disappeared in the presence of MMS; and a ratio of 0 indicates no change of mutation at that position. Orange colour, transition mutation; purple colour, transversion mutation. (**E**) Bar graph showing the proportion of transition mutations in each strain, combining both conditions (green), or separately in the absence (blue) or presence (orange) of MMS.

Under chronic exposure to low MMS concentrations (up to 3 mM), the survival of Δ*polY1* and Δ*polY2* single mutants was similar to that of the wt strain (*P* > 0.05) (Fig. 2A). Survival of the Δ*polY1* Δ*polY2* double mutant was modestly reduced (*P* ∼0.002), whereas survival of the Δ*polA* mutant was dramatically decreased (*P* < 0.00001) relative to the wt strain (Fig. 2A), indicating that PolA activity is highly relevant for cell survival following MMS-induced DNA damage. The survival of the Δ*polY1* Δ*polA* double mutant was similar to that of the Δ*polA* single mutant (*P* > 0.05), whereas inactivation of *polY2* partially suppressed (*P* ∼0.0001) the Δ*polA* survival defect (Fig. 2A), suggesting that PolY2 may generate DNA products that are toxic in the absence of PolA and/or have PolA-independent activities, as proposed earlier [22].

### PolA assists PolY1 or PolY2 to lesion bypass in wt cells

To examine mutagenesis, parallel cultures were grown exponentially under unstressed conditions; half were treated with 3 mM MMS for 15 min, while the remainder were left untreated (Figs 2–6). Because the nature of the damage may modulate TLS, we restricted our analysis to spontaneous and MMS-induced mutagenesis. Mutation frequencies were quantified as Rif^R^ conferring mutations. This assay underestimates mutagenesis, because synonymous substitutions, insertions, deletions, or frameshifts that inactivate the essential *rpoB* gene, or do not confer Rif^R^, are not detected. In addition, mutation rates inferred from phenotypic reporters may not reflect genome-wide mutation frequencies. Nevertheless, this approach provides a practical readout of mutagenic processes that allows comparison with the existing literature to assess the interplay among TLS DNAPs. We note that: (i) spontaneous Rif^R^ mutations arise at a constant rate in isogenic populations and follow Poisson dynamics [56, 57]; (ii) in unperturbed cells, induction of the SOS response is undetectable in bulk assays (<0.1% of total cells) [58]; and (iii) although the term ‘spontaneous mutagenesis’ is imprecise, we use it here to refer to mutations arising in the absence of exogenous DNA damage.

The spontaneous Rif^R^ mutation frequency in wt BG214 cells was 2–3 × 10^−9^ (Fig. 2B), consistent with previous reports [28, 33, 55]. MMS treatment increased the mutation frequency by ∼2- to 3-fold. These results are consistent with those obtained using another 168-derivative strain (R^168^), which also lacks SPβ, PBSX, SKIN, and ICE*Bs*1 [59]. In contrast, the widely used strain 168, which is lysogenic for the SPβ, PBSX, and SKIN prophages and carries the mobile element ICE*Bs*1, showed spontaneous mutation frequencies of 1–3 × 10^−8^, and DNA damage increases the frequency of Rif^R^ mutants ∼14-fold relative to basal levels in the wt strain [43]. These observations suggest that the putative SPβ-encoded TLS DNAP YolD-UvrX, absent in BG214 and R^168^, may account for the ∼10-fold increase in spontaneous mutagenesis and the ∼7-fold increase in damage-induced mutagenesis observed upon SPβ reactivation following SOS response activation. Therefore, these strains may differ in the control of TLS.

Inactivation of either *polY1* or *polY2* caused only modest but statistically significant reductions in spontaneous mutagenesis in exponentially growing cells (<2-fold) relative to wt cells (*P* ∼0.01 and ∼0.008, respectively), with no significant difference between the two single mutants (*P* ∼0.6). In contrast, spontaneous mutagenesis in the Δ*polY1* Δ*polY2* double mutant was strongly reduced (∼9-fold) relative to wt (*P* < 0.00001) (Fig. 2B), indicating that loss of both Y-family polymerases is required to markedly impair spontaneous mutagenesis in the BG214 background.

MMS-induced mutagenesis was modestly though significantly reduced in Δ*polY1* (∼1.6-fold, *P* ∼0.0001), more reduced in Δ*polY2* (∼3-fold, *P* < 0.00001), and profoundly reduced (∼9-fold, *P* < 0.00001) in the Δ*polY1* Δ*polY2* double mutant relative to wt (Fig. 2B). The ratio of MMS-induced to spontaneous mutagenesis was lower in the Δ*polY2* strain, consistent with the absence of DNA damage-induced mutagenesis in the absence of the only SOS-inducible TLS DNAP. Similar trends were observed following exposure to other genotoxic agents (4-nitroquiniline-1-oxide or H_2_O_2_) [28, 33, 55]. Furthermore, comparable results were also obtained in the SPβ-containing background, where either single mutant resembled wt, whereas the double mutant was largely defective in mutagenesis [23, 32].

Deletion of *polA* significantly reduced spontaneous mutagenesis (∼7-fold, *P* < 0.00001) relative to wt, to levels comparable to those observed in the Δ*polY1* Δ*polY2* strain (*P* > 0.5) (Fig. 2B). Spontaneous mutation frequencies in Δ*polY1* Δ*polA* and Δ*polY2* Δ*polA* strains were not significantly different from those of the Δ*polA* single mutant or the Δ*polY1* Δ*polY2* double mutant (*P* > 0.5) (Fig. 2B). Since the contribution of PolY1 and PolY2 to mutagenesis is masked in the Δ*polA* background, this finding suggests that both PolY1 and PolY2 function in concert with the low-fidelity PolA polymerase within the same mutagenic pathway during spontaneous error-prone repair. This interpretation is consistent with the interaction between PolA and the DnaN sliding clamp (Fig. 1) [22], as well as with previous observations (see the ‘Introduction’ section). Similar patterns were observed in the SPβ-containing background, where the strong reduction in mutagenesis in Δ*polY1* Δ*polY2* cells was comparable to that seen in Δ*polA* and Δ*polY1* Δ*polY2* Δ*polA* strains [32].

MMS-induced mutagenesis was reduced ∼3-fold in the Δ*polA* strain, significantly lower than in Δ*polY1* (*P* ∼0.00005) but not significantly different from Δ*polY2* (*P* > 0.05). Notably, MMS-induced mutagenesis was significantly lower in the Δ*polY1* Δ*polY2* double mutant than in the Δ*polA* strain (*P* ∼0.0004), suggesting that PolY1 and/or PolY2 promote mutagenesis in a PolA-independent manner [22]. The Δ*polY1* Δ*polA* strain showed a slight further reduction relative to Δ*polA* (*P* ∼0.01), whereas Δ*polY2* Δ*polA* behaved similar to Δ*polA* (*P* ∼0.4) (Fig. 2B). Comparable results were obtained following treatment with 4-nitroquiniline-1-oxide or H_2_O_2_ [28, 33, 55].

**Figure 7.**
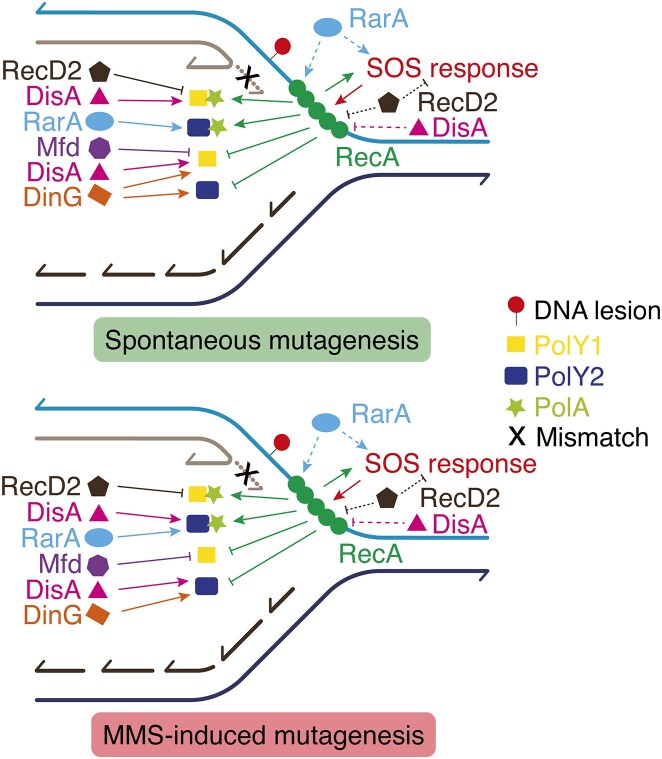
Model for the regulation of TLS in *B. subtilis*. Following replication fork stalling due to endogenous or exogenous DNA damage, TLS DNAPs (PolY1–PolA, PolY2–PolA, PolY1, and PolY2) may catalyse nucleotide incorporation opposite DNA lesions, potentially leading to spontaneous or MMS-induced mutagenesis. Repair functions assembled at stalled forks interact with each other and with RecA and/or PolA, thereby contributing to the regulation of TLS DNAPs activity: (i) RecA may promote PolY1–PolA- and PolY2–PolA-mediated spontaneous and MMS-induced mutagenesis, but inhibit the activities of PolY1 and PolY2 alone; (ii) RecA promotes the SOS response, which in turn increases RecA levels and enhances mutagenesis; (iii) DisA may promote both PolY1–PolA- and PolY1-mediated spontaneous mutagenesis, as well as PolY2–PolA- and PolY2-mediated MMS-induced mutagenesis, in addition to potentially influencing mutagenesis through modulation of RecA activity; (iv) RarA may enhance PolY2–PolA activity, in addition to potentially influencing mutagenesis through promoting RecA filament dynamics and SOS response; (v) RecD2 may inhibit PolY1–PolA-mediated TLS, while also potentially influencing mutagenesis via RecA filament destabilisation and SOS response regulation; (vi) DinG may promote spontaneous mutagenesis mediated by PolY1 and PolY2, and MMS-induced mutagenesis mediated by PolY2; and (vii) Mfd may downregulate PolY1 activity. For simplicity, protein–protein interactions are not depicted. Arrow, stimulation; blunt-ended arrow, inhibition; and dotted line, indirect effect through regulation of RecA activity and/or the SOS response.

Collectively, these data support several conclusions (Fig. 7). First, the constitutively expressed PolY1 and the SOS-inducible PolY2 both contribute to spontaneous and MMS-induced mutagenesis, with partial functional redundancy, as absence of both block mutagenesis, though PolY2 appears to play a more prominent role during MMS-induced mutagenesis, consistent with its induction in response to DNA damage. By contrast, a $\Delta dinB_{Eco}$ mutant displays no detectable phenotype on spontaneous mutagenesis, whereas MMS-induced mutagenesis is strongly reduced in the $\Delta umuDC_{Eco}$ mutant strain [8]. Second, in a two-step TLS mechanism, PolA may substantially assist PolY1 and PolY2 during spontaneous mutagenesis, whereas its contribution to MMS-induced mutagenesis may be less pronounced. At stalled replication forks, DnaN and RecA may interact with and recruit PolA as part of the bipartite PolA–PolY1 and PolA–PolY2 DNAPs. PolA may then position PolY1 or PolY2 at the appropriate termini, allowing these TLS DNAPs to incorporate a limited number of nucleotides to bypass damaged or distorted template bases, albeit at the cost of increased mutagenesis. The resulting short primer may be extended by PolA, thereby fixing the mutation. Then, the termini are handed-off to the replicative PolC DNAP upon replisome reassembly, thereby rescuing DNA synthesis. Third, in Δ*polA* cells, PolY1 and/or PolY2 may promote mutagenesis in a PolA-independent manner [22], and such effect is unlinked to cell death (see Fig. 2A). Indeed, under exogenous DNA damage, the general SOS response increases PolY2 expression [31], which may outcompete PolA for binding to DnaN at sites of damage and to bypass lesions in a one-step process, with direct hand-off of the primer to PolC. This interpretation is consistent with the observations that: (i) DnaN interacts with PolY1, PolY2, and PolA, and PolA interacts with PolY1 and PolY2 (Fig. 1); and (ii) deletion of the PolA SID region, which mediates its interaction with PolY1 and PolY2, reduces mutagenesis when compared to *polA*^+^ cells [12, 22, 30, 32], albeit less severely than complete loss of PolA, supporting the existence of a PolA-independent TLS pathway.

### Deletion of *polY1* and *polY2* does not affect survival of Δ*recA* cells to MMS, but deletion of *polA* does

RecA fulfils multiple functions in the cell [60, 61]. It forms foci in ∼20% of unperturbed *lexA*^+^ or *lexA*(Ind*^−^*), encoding a non-cleavable LexA G92D protein variant that is defective in SOS response induction [62], and this proportion increases to >85% following exposure to UV doses insufficient to trigger the SOS response in wt cells [20]. These RecA foci colocalise with replisome markers in >90% of cells. RecA regulates error-free DDT sub-pathways *in vivo* [16, 43] and PriA-dependent replication initiation *in vitro* [63]. If ssDNA regions persist, RecA bound to ssDNA triggers either a local SOS response or a global SOS response in response to exogenous threats, promoting autocleavage of the LexA repressor and increasing expression of the PolY2 polymerase [31, 43].

The viability of the Δ*recA* single mutant was reduced ∼5-fold even in the absence of exogenous DNA damage, as previously reported [64]. Here, we observed that the plating efficiency of the Δ*recA* Δ*polY2*, and Δ*recA* Δ*mutSL* strains was comparable to that of the Δ*recA* strain, whereas plating efficiency was further reduced ∼2-fold in the Δ*recA* Δ*polY1* strain and ∼10-fold in the Δ*recA* Δ*polA* strain.

Because Δ*recA* mutants are extremely sensitive to MMS, lower MMS concentrations were used in these experiments. At MMS concentrations up to 0.1 mM, the survival of the Δ*polY1*, Δ*polY2*, Δ*polA*, and Δ*mutSL* mutants did not differ significantly from that of the wt strain (*P* > 0.05). Survival of the Δ*recA* Δ*polY1*, Δ*recA* Δ*polY2*, and Δ*recA* Δ*mutSL* mutants was reduced ∼900-fold relative to wt (*P* < 0.0002), similar to the Δ*recA* single mutant (*P* > 0.05) (Fig. 3A). The Δ*recA* Δ*polA* strain was extremely sensitive to 0.1 mM MMS, with survival reduced 10-fold relative to the Δ*recA* strain (*P* ∼0.004) (Fig. 3A). This finding is consistent with the multifunctional nature of RecA and PolA, which likely act both cooperatively through a physical interaction (Fig. 1) and independently: RecA is the central player of homologous recombination; and PolA—reported to possess a robust reverse transcriptase activity—may bypass long stretches of templated ribonucleotides [65], and its 5′ → 3′ polymerase activity may also contribute to excision repair pathways.

To address the contribution of RecA levels and SOS response activation to TLS, Δ*lexA* and *lexA*(Ind^−^) strains were analysed. First, RecA levels in these strains were quantified by immunoblotting (Supplementary Fig. S1). MMS moderately induced the SOS response in wt cells. No RecA signal was observed in the Δ*recA* strain. In the Δ*lexA* strain, *recA* was constitutively expressed at levels comparable to those observed under full SOS induction (∼0.6 μM mitomycin C, ∼25 000 monomers/cell) in wt cells [48]. In contrast, the *lexA*(Ind^−^) variant did not alter basal RecA levels (estimated at ∼4500 monomers/cell), and increasing MMS concentrations failed to induce RecA accumulation. These results confirm that RecA levels in the Δ*lexA, lexA*(Ind^−^), and Δ*recA* strains are as expected [48].

The Δ*lexA* mutation conferred increased tolerance to alkylation damage (*P* ∼0.003). In contrast, survival of the *lexA*(Ind^−^) strain was reduced relative to the Δ*polY1*, Δ*polY2*, or wt strains upon chronic exposure to 3 mM MMS (*P* < 0.00001) (Fig. 3B). Survival of the Δ*lexA* Δ*polA* strain was reduced relative to wt (*P* ∼0.0002), similar to the Δ*polA* single mutant (*P* > 0.05), whereas survival of the *lexA*(Ind^−^) Δ*polA* strain was further reduced relative to wt (*P* < 0.00001) (Fig. 3B). These data suggest that: (i) induction of the SOS response promotes, and is required for, survival following MMS exposure; and (ii) SOS induction, or elevated RecA levels, is essential for coping with alkylation DNA damage in the absence of PolA.

### Inactivation of *recA* increases mutagenesis in the Δ*polA* context

We next tested whether RecA, by modulating the balance between error-free and error-prone pathway choice and/or by affecting the loading or activity of DDT factors, directly or indirectly alters the frequency of mutations. Deletion of *recA* significantly reduced both spontaneous and MMS-induced mutagenesis (∼4- and ∼8-fold, respectively, *P* < 0.00001) relative to wt cells. Consistent with the lack of SOS-induction in the absence of RecA, MMS failed to increase mutagenesis (Fig. 3C).

To assess whether RecA influences MMR, mutagenesis was analysed in the Δ*mutSL* background. Inactivation of *mutS* and *mutL* (Δ*mutSL*) caused a dramatic increase in spontaneous and MMS-induced mutation frequencies relative to wt (∼115- and ∼150-fold, respectively, *P* < 0.0001), consistent with the role of MMR in correcting replication and TLS-associated errors post-replicatively [35, 36, 66, 67]. In the Δ*recA* Δ*mutSL* mutant, spontaneous mutagenesis was not significantly different from that in Δ*mutSL* (*P* > 0.05), whereas MMS-induced mutagenesis was slightly reduced (*P* ∼0.02), and the MMS-induced/spontaneous mutagenesis ratio was decreased, as in the Δ*recA* single mutant (Fig. 3C). These data indicate that RecA contributes to MMS-induced error-prone repair independently of MMR.

The spontaneous and MMS-induced mutation frequencies in Δ*recA* Δ*polY1* and Δ*recA* Δ*polY2* strains were significantly lower than in the corresponding single Δ*polY1* and Δ*polY2* mutants (*P* < 0.001). However, only MMS-induced mutagenesis in Δ*recA* Δ*polY2* was significantly reduced relative to Δ*recA* (∼2-fold, P ∼0.02) (Fig. 3C). In both cases, the MMS-induced/spontaneous mutagenesis ratio was reduced, as observed in the Δ*recA* strain.

Unexpectedly, both spontaneous and MMS-induced mutagenesis frequencies in the Δ*recA* Δ*polA* strain were increased (∼5- and ∼2-fold, respectively, *P* < 0.0001) relative to the Δ*polA* single mutant, and ∼3- and ∼7-fold (*P* < 0.0001), respectively, relative to the Δ*recA* strain. Notably, both the proportion of Rif^R^ mutants and the MMS-induced/spontaneous mutagenesis ratio were similar to those of wt (Fig. 3C), indicating that SOS-independent mutagenesis operates in the Δ*recA* Δ*polA* background. This contrasts with *E. coli*, in which loss of RecA*_Eco_* and SOS induction results in a non-mutable phenotype, and absence of PolA*_Eco_* does not affect mutagenesis (reviewed in [8, 15]).

Together, these results support a dual role for RecA in TLS in *B. subtilis* (Fig. 7). First, RecA assembled at stalled forks, as a mutator, likely interacts with and positions PolA at lesions, thereby promoting two-step error-prone TLS, as deletion of RecA markedly reduces TLS, regardless of the presence of PolY1 or PolY2. Second, RecA may also function as an anti-mutator by limiting PolA-independent TLS mediated by PolY1 or PolY2, as revealed by the increased mutagenesis observed in the Δ*recA* Δ*polA* background. This inhibitory effect may reflect that RecA restricts or competes DnaN-mediated loading of PolY1 or PolY2 onto primer-termini. Since PolY1 and PolY2 TLS pathways remain operative in the absence of both RecA and PolA, YqjX may not be required for PolY2 activity in *B. subtilis* (see the ‘Introduction’ section). It remains unclear whether a potential PolY2·YqjX complex can function in the absence of RecA, or whether PolY2 works in concert with the SPβ-encoded YolD protein. Finally, an indirect effect of RecA via modulation of the SOS response cannot be excluded.

### Altering RecA levels and SOS induction modulates mutagenesis

Because, in the absence of PolA, induction of the SOS response—and thus elevated levels of RecA—is required to tolerate DNA damage (Fig. 3B), and RecA limits mutagenesis in this context (Fig. 3C), we next examined whether the extent of SOS induction modulates PolA-dependent mutagenesis.

Deletion of *lexA*, which results in constitutive expression of SOS genes (Supplementary Fig. S1), significantly increased both spontaneous and MMS-induced mutation frequencies (∼17- and ∼9-fold, respectively, *P* < 0.00001) relative to wt. Consistent with full SOS induction in the absence of exogenous damage, the MMS-induced/spontaneous mutagenesis ratio was ∼1. Deletion of *lexA* in the Δ*polA* background significantly increased spontaneous and MMS-induced mutagenesis relative to the Δ*polA* single mutant (*P* ∼0.01 and ∼0.0002, respectively), but resulted in extremely lower mutagenesis than that observed in the Δ*lexA* strain (*P* ∼0.00004) (Fig. 3D). These results reinforce our previous conclusions: (i) constitutive SOS induction, leading to elevated RecA and PolY2 levels, promotes error-prone repair; and (ii) in the absence of PolA, RecA functions as an anti-mutator.

Unexpectedly, in the *lexA*(Ind^−^) strain, in which no SOS induction occurs even in the presence of MMS, and, therefore RecA levels do not increase (Supplementary Fig. S1) and PolY2 levels are not expected to rise, the spontaneous mutation frequency was comparable to that observed under constitutive SOS induction in the Δ*lexA* strain (*P* > 0.05). However, MMS-induced mutagenesis was similar to that of wt (*P* > 0.05). Upon deletion of *polA* in this background, the spontaneous mutation frequency decreased to wt levels, and MMS-induced mutagenesis was reduced to levels comparable to those of the Δ*polA* strain (*P* > 0.05) (Fig. 3D). This strongly contrasts with *E. coli*, in which lesion bypass by TLS DNAPs is minimal in the absence of SOS induction [10].

These results cannot be explained solely by considering RecA levels. Several, non-mutually exclusive hypotheses may account for these phenotypes: (i) the LexA G92D variant may differentially interact with RecA and alters its conformation, filament stability, or activity, thereby promoting its interaction with PolA and/or other factor(s) that enhance mutagenesis; or (ii) yet unidentified factor(s) may drive mutagenesis in the *lexA*(Ind^−^) background.

Collectively, these data indicate that: (i) the extent of misincorporation is not determined solely by the cellular abundance of DNA-damage inducible proteins; and (ii) a critical level of RecA engaged at stalled forks is required both to promote PolA-dependent TLS and to antagonise PolA-independent mutagenic pathways.

### Inactivation of *rarA* increases survival of cells lacking TLS DNAPs in response to MMS

Previous work has shown that proteins loaded at stalled replication forks, which directly or indirectly interact with RecA and/or PolA, modulate mutagenesis levels: RarA, DisA, and DinG promote mutagenesis; whereas RecD2 and Mfd suppress it. These factors are therefore attractive candidate regulators of mutagenic DNAPs and of the balance between error-free and error-prone DDT pathways [27, 28, 33, 55], and their contribution is further characterised here.

RarA functions as a positive modulator of RecA filament growth with inactivation of *rarA* reducing the formation of visible RecA threads *in vivo* following DNA damage [64]. In addition, SsbA interacts with and recruits RarA to the replication fork even in the absence of DNA damage [68]. RarA preferentially binds ssDNA and replication fork structures and, together with SsbA, slows PriA-dependent DNA replication restart *in vitro*, thereby preventing recombination intermediates from promoting pathological DNA replication restart events [24, 55, 64].

Upon chronic exposure to increasing concentrations of MMS, survival of the Δ*rarA* strain was modestly increased relative to wt cells (*P* ∼0.03) (Fig. 4A). The Δ*rarA* mutation also modestly increases the survival of Δ*polY2* cells (*P* ∼0.02) and moderately increased the survival of Δ*polA* cells (*P* ∼0.0005), while having no detectable effect on the survival of Δ*polY1* strains (*P* > 0.05) upon chronic exposure to 3 mM MMS (Fig. 4A). The Δ*mutSL* mutant, used as a control, displayed increased tolerance to alkylation damage, with survival enhanced ∼3-fold (*P* ∼0.003), and deletion of *rarA* has no effect on its survival following MMS treatment (*P* > 0.05) (Fig. 4A). In the wt background, or in the absence of *polY2* or *polA*, deletion of *rarA* may prevent the formation of toxic intermediates that compromise cell survival, such as those arising from unnecessary RecA-mediated SOS response activation or recombinational activity, which are facilitated by RarA [64].

### RarA promotes mutagenesis in the Δ*polY1* and Δ*polA* backgrounds

RarA does not appear to interact with TLS DNAPs (Fig. 1), but has been shown to interact with RecA by *in vitro* assays [64]. Both spontaneous and MMS-induced mutagenesis were reduced (∼4- and ∼3-fold, respectively) in the Δ*rarA* strain relative to wt (*P* < 0.00001) (Fig. 4B), consistent with previous reports [55]. Inactivation of *rarA* did not significantly affect spontaneous or MMS-induced mutagenesis in the Δ*polY2* or Δ*mutSL* contexts, when compared with their Δ*polY2* or Δ*mutSL* parental strains (*P* > 0.05). In contrast, deletion of *rarA* markedly reduced both spontaneous and MMS-induced mutagenesis in the Δ*rarA* Δ*polY1* and Δ*rarA* Δ*polA* strains relative to the corresponding single parental mutants (*P* < 0.00001) (Fig. 4B), uncovering a novel role for RarA in promoting error-prone DDT sub-pathways, especially PolA-dependent PolY2 activity (Fig. 7).

RarA may contribute to error-prone DDT by facilitating the recruitment of TLS DNAPs at sites of DNA damage. A similar role has been proposed for the eukaryotic Mgs1 and WRNIP1 proteins, which exhibit significant sequence identity to RarA [69]. We cannot exclude an indirect effect of RarA on mutagenesis, as RarA promotes RecA filament formation, and therefore deletion of *rarA* compromises the SOS response [64] altering RecA levels, which strongly influence mutagenesis (Fig. 3).

### *recD2* is not epistatic with *polY1, polY2*, and *polA* in response to MMS

RecD2, which is absent in *E. coli* cells, acts as a negative modulator of RecA by promoting the disassembly of RecA nucleoprotein filaments from ssDNA, thereby limiting RecA activities, including inhibition of PriA-dependent replication initiation or SOS induction [27, 64]. However, in conjunction with SsbA, RecD2 regulates replication restart [27]. In addition, RecD2 catalyses 5′ → 3′ branch migration and strand switching on forked DNA substrates [70].

Survival of the Δ*recD2* single mutant was reduced following chronic exposure to 3 mM MMS relative to the wt strain (*P* ∼0.0005) (Fig. 4C), consistent with previous observations [23]. Survival of the Δ*recD2* Δ*mutSL* mutant was comparable to that of the Δ*recD2* single mutant (*P* > 0.05). In contrast, survival of the Δ*recD2* Δ*polY2* strain was moderately reduced (*P* ∼0.02) relative to Δ*polY2* strain, but markedly increased relative to the Δ*recD2* strain (*P* ∼0.0007). This finding suggests that loss of PolY2 suppresses the DNA repair defect of Δ*recD2* cells, or the accumulation of DNA products that may be toxic in the Δ*recD2* context. Survival of the Δ*recD2* Δ*polY1* strain was strongly reduced relative to the Δ*polY1* (*P* < 0.00001) and Δ*recD2* strain (*P* ∼0.0006), and survival of the Δ*recD2* Δ*polA* strain was significantly reduced (*P* ∼0.004) compared with the Δ*polA* strain (Fig. 4C). These results indicate that *recD2* is not epistatic with *polY1, polY2*, or *polA* in response to MMS-induced DNA damage (Fig. 4C). This is consistent with the multifunctional role of RecD2 in the cell.

### RecD2 decreases mutagenesis

RecD2 physically interacts with RecA, PolA, and MutS (Fig. 1). Both spontaneous and MMS-induced mutagenesis were increased in the Δ*recD2* strain relative to the wt control (∼4-fold, *P* < 0.00001) (Fig. 4D). Deletion of *recD2* did not significantly affect the spontaneous mutation frequency of Δ*recD2* Δ*polY1* cells when compared with the Δ*polY1* strain (*P* > 0.05), whereas MMS-induced mutagenesis was increased by ∼4-fold (*P* < 0.00001). However, both frequencies were significantly lower than in the Δ*recD2* single mutant (*P* < 0.001). In the Δ*recD2* Δ*polY2* strain, both spontaneous and MMS-induced mutation frequencies were significantly increased relative to the Δ*polY2* single mutant (*P* < 0.00001), but were comparable to those observed in the Δ*recD2* single mutant (*P* > 0.05) (Fig. 4D). Similarly, spontaneous and MMS-induced mutagenesis were also increased in the Δ*recD2* Δ*polA* strain when compared with Δ*polA* (∼2- and ∼3- fold, respectively, P ∼0.001), but remained strongly reduced relative to the Δ*recD2* strain (∼13- and ∼4-fold, respectively, *P* < 0.00001). Therefore, RecD2 may restrain PolY1–PolA-dependent TLS (Fig. 7), although the precise molecular mechanism remains to be determined. Moreover, because RecD2 promotes RecA filament disassembly [27], increased RecA filament stability and elevated SOS induction and RecA levels in Δ*recD2* cells may also contribute to the elevated mutagenesis observed in this background.

In the absence of RecD2 and MutSL, spontaneous mutagenesis was modestly increased (∼1.5-fold, *P* ∼0.01), whereas MMS-induced mutagenesis was slightly reduced (∼1.6-fold, *P* ∼0.002) relative to the Δ*mutSL* strain (Fig. 4D). Therefore, a contribution to MMR cannot be definitively excluded.

### Inactivation of *rarA* antagonises Δ*recD2* in response to MMS

As introduced above, the RecA modulators RarA and RecD2 act during RecA-mediated homology search and strand exchange, but exert opposing activities: RarA promotes RecA filament growth, whereas RecD2 facilitates RecA filament disassembly [27, 64].

In previous sections, we showed that: (i) the Δ*rarA* mutation partially suppresses the sensitivity of Δ*polA* cells to MMS (Fig. 4A); and (ii) Δ*recD2* Δ*polA* is markedly more sensitive to MMS than the Δ*polA* strain (Fig. 4C). To address the apparent antagonistic roles of RarA and RecD2, the Δ*rarA* mutation was introduced into the Δ*recD2* Δ*polA* background (see Supplementary Table S1). Deletion of *rarA* suppressed the MMS sensitivity caused by loss of *recD2* (*P* ∼0.0002), as survival of the Δ*recD2* Δ*rarA* strain was comparable to that of the Δ*rarA* single mutant (*P* > 0.05). Inactivation of RarA also counteracted the extreme MMS sensitivity imposed by the Δ*recD2* mutation in the Δ*polA* context (*P* ∼0.0001), with survival of the Δ*recD2* Δ*rarA* Δ*polA* strain restored to levels comparable to those of the Δ*polA* strain (*P* > 0.05) (Fig. 4E). Two non-mutually exclusive hypotheses may explain this outcome: (i) removal of the positive RecA modulator RarA alleviates the toxic consequences of uncontrolled RecA activity or increased SOS induction in the absence of RecD2; and/or (ii) RarA contributes to the formation of DNA intermediates that are toxic in the absence of RecD2.

### RecD2 antagonises RarA in mutagenesis in the Δ*polA* context

RarA enhances mutagenesis (Fig. 4B), whereas RecD2 reduces mutagenesis (Fig. 4D). We can hypothesise that their antagonistic role in mutagenesis may, at least in part, reflect their counteracting activities in regulating RecA filament dynamics, as previously observed following DNA damage (Fig. 4E).

The Δ*rarA* mutation partially suppressed the increase in spontaneous and MMS-induced mutagenesis observed in the Δ*recD2* background (*P* ∼0.04 and ∼0.003, respectively), although mutagenesis remained elevated relative to wt cells (*P* ∼0.04) (Fig. 4F). In contrast, mutagenesis in the Δ*recD2* Δ*rarA* Δ*polA* strain was similar to that in the Δ*recD2* Δ*polA* strain (*P* > 0.05), indicating that the antagonistic role of RarA is not apparent in the absence of PolA (Fig. 4F). Notably, in both Δ*recD2* Δ*rarA* and Δ*recD2* Δ*rarA* Δ*polA* strains, mutagenesis was significantly higher than in the corresponding Δ*rarA* and Δ*rarA* Δ*polA* strains, respectively (*P* < 0.01).

Together, these data indicate that RarA by increasing and RecD2 by reducing RecA filament growth may antagonistically regulate error-prone DDT and mutagenesis, with RecD2 exerting a dominant influence (Fig. 7). However, their impact on mutagenesis cannot be explained solely by modulation of RecA filament stability. In particular, RecA acts predominantly as an anti-mutator in the absence of PolA (Fig. 3C), and therefore increased filament stability would be expected to reduce mutagenesis; yet the opposite trend is observed. This suggests that RarA and RecD2 also influence mutagenesis through RecA-independent mechanisms, potentially by modulating access of TLS polymerases to stalled forks or the processing of recombination and repair intermediates.

### Inactivation of *polY1* or *polY2* does not affect survival of Δ*disA* cells in response to MMS

DisA, which is absent in *E. coli* cells, functions as a DNA damage checkpoint protein that scans the chromosome for lesions and recognises branched DNA intermediates formed at stalled replication forks in concert with RecA [26, 71]. Binding of DisA to these intermediates limits the activity of RecA and fork remodellers, thereby regulating the engagement of error-free and error-prone DDT sub-pathways [33, 72].

Survival of the Δ*disA* strain was reduced following chronic exposure to 3 mM MMS relative to the wt strain (*P* ∼0.002) (Fig. 5A). Survival of the Δ*disA* Δ*polY1* and Δ*disA* Δ*polY2* strains was comparable to that of the Δ*disA* single mutant (*P* > 0.05). In contrast, survival of the Δ*disA* Δ*polA* strain was significantly reduced relative to both the Δ*disA* (*P* ∼0.0004) and Δ*polA* (*P* ∼0.004) strains. Inactivation of MMR restored survival of the Δ*disA* strain to levels similar to those observed in the wt background (Fig. 5A). These results indicate that *disA* is not epistatic with *polA* or *mutSL* in response to MMS-induced DNA damage.

### DisA promotes mutagenesis

DisA interacts with RecA and PolA (Fig. 1). Loss of DisA decreased the frequency of spontaneous (∼4-fold) and MMS-induced Rif^R^ mutants (∼2-fold) relative to wt cells (*P* < 0.00001) (Fig. 5B), consistent with previous observations [33]. Inactivation of *disA* did not affect the spontaneous mutation frequency observed in the Δ*polY1* background (*P* > 0.05), which remained higher than in Δ*disA* strain (*P* ∼0.0004). By contrast, deletion of *disA* decreased spontaneous mutagenesis in the Δ*polY2* mutant (*P* ∼0.0002), to levels comparable to those observed in the Δ*disA* strain (*P* > 0.05).

MMS-induced mutation frequency was moderately reduced in the Δ*disA* Δ*polY1* strain compared with Δ*polY1* (*P* ∼0.0005) but was similar to that of the Δ*disA* strain (*P* > 0.05) (Fig. 5B). In the Δ*disA* Δ*polY2* background, MMS-induced mutagenesis was comparable to that of the Δ*polY2* strain (*P* > 0.05), but lower than that of the Δ*disA* strain (*P* < 0.00001), in agreement with previous observations [33]. The Δ*disA* Δ*polA* strain displayed spontaneous mutation frequencies similar to those of the Δ*polA* strain (*P* > 0.05), whereas MMS-induced was slightly reduced (∼2-fold, *P* ∼0.01).

Together, these data indicate that DisA, as a checkpoint protein [71], may sense DNA damage at the stalled fork and promote mutagenesis, likely by favouring distinct error-prone DDT pathways: spontaneous mutagenesis mediated by PolY1 and MMS-induced mutagenesis mediated by PolY2, including PolA-independent pathways (Fig. 7). Given that *disA* is epistatic to *recA* in response to MMS-induced DNA damage, and that DisA interacts with and regulates RecA activities [26], an indirect effect on mutagenesis via modulation of RecA cannot be excluded.

Loss of DisA did not significantly affect spontaneous mutagenesis in the Δ*mutSL* background (*P* > 0.05), but moderately reduced MMS-induced mutagenesis (∼2-fold, *P* ∼0.0001) (Fig. 5B). Nevertheless, mutation frequencies in the absence of DisA remain far below the hypermutable phenotype of Δ*mutSL*, indicating that DisA is unlikely to play a major role in modulating MMR activity.

### Inactivation of *dinG* partially suppresses the repair defect of Δ*polA* in response to MMS

DinG is a 3′ → 5′ exo(ribo)nuclease that may contribute to mitigate replication–transcription conflicts (RTCs) as *in vitro* it degrades the nascent leading strand at stalled forks and the RNA strand of RNA–DNA hybrids, thereby removing R-loops [28]. This activity is absent in $DinG_{Eco}$. Single-molecule live-cell imaging revealed that DinG forms spontaneous foci at or near replication forks, which become enriched following MMS treatment [28].

At MMS concentrations up to 3 mM, survival of the Δ*dinG* strain was comparable to that of the wt strain (*P* > 0.05). Upon chronic exposure to increasing MMS concentrations, survival of the Δ*mutSL* Δ*dinG* strain increased relative to the Δ*mutSL* strain (*P* ∼0.02) (Fig. 5C). Similarly, inactivation of *dinG* moderately increased (*P* ∼0.004) the survival of Δ*dinG* Δ*polA* cells compared with the Δ*polA* strain (Fig. 5C). In contrast, survival of the Δ*dinG* Δ*polY2* strain was moderately reduced (*P* ∼0.003), and survival of the Δ*dinG* Δ*polY1* strain was strongly reduced (*P* ∼0.001), relative to the Δ*polY2* or Δ*polY1* strain, respectively (Fig. 5C). These results suggest that: (i) *dinG* is not epistatic with *polY1* or *polY2* in response to MMS-induced DNA damage; and (ii) DinG may promote the formation of DNA intermediates that are toxic in the absence of PolA or MMR.

### DinG promotes mutagenesis

DinG physically interacts with PolA and MutS (Fig. 1), and with RecA [28]. Deletion of *dinG* reduced both spontaneous and MMS-induced mutagenesis by ∼3- and ∼2-fold, respectively, relative to the wt strain (*P* < 0.00001) (Fig. 5D), consistent with previous reports [28]. Inactivation of *dinG* significantly reduced spontaneous mutagenesis in the Δ*polY1* and Δ*polY2* backgrounds to levels comparable with those observed in Δ*dinG* cells (*P* > 0.05), whereas it did not significantly affect spontaneous mutagenesis in the Δ*polA* background (*P* > 0.05) (Fig. 5D). Deletion of *dinG* had no significant effect on MMS-induced mutagenesis in the Δ*polY2* or Δ*polA* contexts (*P* > 0.05), but significantly reduces MMS-induced mutagenesis in the Δ*polY1* background (*P* ∼0.0004), again to levels comparable with Δ*dinG* cells (*P* > 0.05). Conversely, in the Δ*mutSL* background, deletion of *dinG* markedly reduced both spontaneous and MMS-induced mutagenesis (∼17- and ∼19-fold, respectively, *P* < 0.0001) (Fig. 5D).

Together, these data indicate that DinG promotes error-prone DDT sub-pathways, likely indirectly through its interaction with PolA and/or RecA (Fig. 7). In addition, in the absence of MMR, DinG strongly contributes to mutagenesis, potentially as a consequence of defects in RTC processing, which could impact transcription-associated mutagenesis. Because mutation frequencies in Δ*dinG* cells remain far lower than those observed in Δ*mutSL* strain, DinG is unlikely to play a direct role in modulating MMR itself.

### Inactivation of *mfd* does not affect survival of Δ*polA* cells in response to MMS

The Mfd translocase associates with elongation transcription complexes and functions as a transcription-coupled repair factor [51]. In addition, Mfd operates at the interface between transcription and replication by removing stalled RNAPs. Thereby, Mfd contributes to DNA repair and resolution of RTCs, while also promoting transcription-associated mutagenesis [73]. However, depending on the cellular context, Mfd has been reported to function either as an anti-mutator or as a mutator [73].

Previous studies showed that survival of the Δ*mfd* strain is reduced following acute exposure to 10 mM MMS relative to the wt strain [51]. However, under chronic exposure to 3 mM MMS, survival of the Δ*mfd*, Δ*mfd* Δ*polY1*, and Δ*mfd* Δ*polY2* strains was comparable to that of wt cells (*P* > 0.05) (Fig. 5E). Survival of the Δ*mfd* Δ*polA* strain was similar to that of the Δ*polA* single mutant (*P* > 0.05). Survival of the Δ*mfd* Δ*mutSL* strain was modestly reduced (*P* ∼0.008) relative to the Δ*mutSL* strain, to levels comparable to the Δ*mfd* strain (*P* > 0.05) (Fig. 5E).

### Mfd inactivation inhibits mutagenesis in the Δ*polA* context

Mfd physically interacts with PolA (Fig. 1), and with RNAP [51]. Under our experimental conditions, the Δ*mfd* mutation had a modest but statistically significant effect on spontaneous and MMS-induced mutagenesis (<2-fold increase, *P* ∼0.02) relative to the wt strain. Inactivation of Mfd did not significantly affect either spontaneous or MMS-induced mutagenesis in the Δ*polY1* background (*P* > 0.05). In contrast, deletion of *mfd* in the Δ*polY2* background resulted in a strong increase in both spontaneous and MMS-induced mutagenesis (∼9- and ∼14-fold, respectively, *P* < 0.0001). This suggests that Mfd limits the mutagenic activity of PolY1 (Fig. 7).

Conversely, both spontaneous and MMS-induced mutagenesis were reduced (∼2- and ∼3-fold, *P* ∼0.01 and ∼0.0005, respectively) in the Δ*mfd* Δ*polA* strain relative to the Δ*polA* strain (Fig. 5F), suggesting that Mfd acts as a mutator in the Δ*polA* background. This is consistent with the fact that accumulation of Leu^+^ revertants during stationary-phase requires both PolA and Mfd, as it is reduced ∼8-fold in the Δ*mfd* Δ*polA* background, indicating that Mfd upon interacting with and recruiting PolA to perform error-prone repair are part of a pathway that is additional to and distinct from canonical transcription-coupled repair [73].

Notably, spontaneous and MMS-induced mutagenesis were markedly reduced (∼5- and ∼14-fold, respectively, *P* < 0.0001) in the Δ*mfd* Δ*mutSL* strain relative to Δ*mutSL* (Fig. 5F), suggesting that, in the absence of MMR, Mfd strongly promotes error-prone DDT sub-pathways and/or transcription-associated mutagenesis. A similar phenotype was observed in the Δ*dinG* Δ*mutSL* background (Fig. 5D). Given that both Mfd and DinG function in the resolution of RTCs, this phenotype may reflect defective processing of RTCs, which in turn impacts transcriptional-associated mutagenesis.

### Mutation spectra confirm the prominent role of RecA in regulating TLS

To further elucidate the mechanism of action of TLS DNAPs and their regulation, the *rpoB* gene from Rif^R^ isolates was sequenced to map mutations conferring Rif^R^ in each genetic background.

Across all strain backgrounds, most Rif^R^-conferring mutations mapped to Cluster I of RpoB, specifically within the Rif-binding pocket (Fig. 6A and B, and Supplementary Table S6) [74]. However, the mutation spectrum was not uniform. Mutations strongly clustered at a single amino acid position, H482, a well-established high-frequency Rif^R^ hotspot in *B. subtilis* and many bacteria [75]. Two substitutions, H482Y (C → T, by far the most frequent, *P* < 0.00001) and H482R (A → G), were detected in nearly every strain, regardless of genetic background or MMS treatment. Their dominance suggests that: (i) they confer the highest fitness among resistance mutations even when DNA repair pathways are altered; and (ii) most genetic backgrounds permit the underlying mutational events (transition or transversion) that generate them.

In the absence of MMS, the mutation spectrum was more diverse (Fig. 6B and Supplementary Table S6). Following MMS treatment, however, the spectrum collapsed towards H482Y (dominant, 63% [+MMS] versus 42% [−MMS], *P* < 0.00001), with occasional Q469R (A → G, 12% [+MMS] versus 7% [−MMS], *P* ∼0.002). In contrast, H482R (24% [−MMS] versus 14% [+MMS], *P* ∼0.004), H482P (A → C, 11% [−MMS] versus 5% [+MMS], *P* ∼0.0001), and Q469K (C → A, 4.3% [−MMS] versus 1.8% [+MMS], *P* < 0.00001) were less frequent after MMS exposure. Mutations V135F (G → T), L415I (C → A), Q469L (A → T), and outside *rpoB* were observed only in the absence of MMS, whereas S487L (C → T) and the double mutation H482Y+S487L were detected exclusively after MMS treatment. Therefore, MMS markedly enriched the dominance of the ‘best’ Rif^R^-conferring *rpoB* alleles (notably H482Y: wt [5 → 8 events], Δ*polA* [2 → 10], Δ*dinG* [2 → 10], and Δ*rarA* [3 → 10]). These findings indicate that MMS biases the mutational mechanisms, imposing a stronger selection pressure and/or a mutational/repair context that makes those solutions more likely to appear and/or fix.

Mutations at Q469 (Q469K/R/L) were less frequent and showed no consistent enrichment following MMS treatment, suggesting either lower fitness or distinct mutational mechanism origins (Fig. 6B and C, and Supplementary Table S6). Furthermore, in many genetic backgrounds, Q469 mutations are only observed either in the absence of MMS or following MMS treatment. Q469 substitutions are common in wt, Δ*polA*, Δ*polY2*, Δ*mutSL*, Δ*mfd*, Δ*disA*, and Δ*recD2*, but rare or absent in Δ*polY1* and Δ*recA*. This distribution suggests that DNAP usage and proofreading capacity significantly influence the mutational spectrum.

Sequencing of independent Rif^R^ clones of the wt strain revealed non-synonymous substitutions exclusively within Cluster I of RpoB, predominantly at Q469 and H482: Q469K, Q469R, H482Y, and H482P (Fig. 6C and D, and Supplementary Table S6). These substitutions are consistent with inherent replication errors and endogenous DNA metabolic processes that escape correction by PolC proofreading and MMR. This agrees with previous reports in wt *B. subtilis* and *E. coli*, where spontaneous Rif^R^ mutations are typically single nucleotide substitutions at these positions [74]. The fitness costs of these mutations were not assessed; however, Q469R and H482Y did not seem to impair growth in LB medium. MMS primarily induces purine lesions, and extension of purine:purine mismatches is generally inefficient for many DNAPs. Therefore, the mutational signature observed following MMS treatment is consistent with TLS activity rather than defects in PolC proofreading or MMR (see [59]).

In Δ*polY1* and Δ*polY2* strains, mutations remained confined to Cluster I, as in wt, with strong enrichment of H482Y, both in the absence or presence of MMS (Fig. 6C and D, and Supplementary Table S6). This suggests that these DNAPs share partial functional redundancy (Fig. 2B), as the absence of a single DNAP does not significantly affect the mutational spectrum.

In the absence of MMS, Δ*polA* displayed the broadest spectrum (six different mutations) (Fig. 6C and D, and Supplementary Table S6). Rare Cluster N mutation V135F was observed (30% of events), and Q469 substitutions were overrepresented (40% of events), including Q469K, Q469L, and Q469R. Notably, Q469L was unique to this background. These findings suggest that without PolA-mediated DNA extension following PolY1 or PolY2 activity, a broader spectrum of mutations is generated. Following MMS treatment, however, the spectrum collapsed to H482Y, indicating that MMS-induced C → T transitions override strain-specific mutational biases.

Δ*mutSL* colonies exhibited the most distinct mutational spectrum, consistent with defective MMR (Fig. 6C and D, and Supplementary Table S6). Although mutations remained within Cluster I, H482R was significantly enriched (90% [−MMS]; 60% [+MMS], *P* < 0.00001), and no enrichment of H482Y following MMS treatment was observed. Q469R was also overrepresented (30% [+MMS], *P* < 0.00001). Thus, loss of MMR significantly reshapes the effective mutational spectrum.

In Δ*recA* cells, all mutations mapped to H482, with strong enrichment of H482P (70% [−MMS]; 50% [+MMS], *P* < 0.00001), a mutation otherwise rare (Fig. 6C and D, and Supplementary Table S6). This supports a major role for RecA in regulating TLS activity, as previously suggested (Fig. 3C). Alternatively, H482P may have distinct fitness effects tolerated in the Δ*recA* background.

These distinct mutational spectra suggest that: (i) most base substitutions introduced by PolC or TLS DNAPs are normally corrected by MMR; and (ii) MMR-mediated correction of misincorporations occurs largely independently of RecA.

In Δ*disA*, all substitutions mapped to Cluster I (Fig. 6C and D, and Supplementary Table S6). The rare S487L mutation appeared after MMS treatment. Moreover, the only colony showing two point mutations (H482Y+S487L) was detected. In the absence of MMS, in contrast, H482Y predominated (80% of events). These data may indicate increased mutational load or sequential mutation accumulation in the absence of DisA.

In Δ*rarA*, H482R was enriched in the absence of MMS, similar to Δ*mutSL* (Fig. 6C and D, and Supplementary Table S6). V135F appeared in Δ*rarA*, as in Δ*polA*. One Rif^R^-conferring mutation mapped outside *rpoB*. After MMS treatment, however, the spectrum collapsed to H482Y. In the absence of RecD2, only H482 substitutions were detected. After MMS treatment, however, Q469R was strongly enriched (50% of events, *P* < 0.00001), suggesting that RecD2 contributes to suppress A → G transitions following MMS-induced DNA damage. The exclusive presence of transitions within Cluster I supports a role in regulating error-prone DDT rather than primary MMR deficiency.

Δ*dinG* colonies exhibited the most complex mutational spectrum (Fig. 6C- and D, and Supplementary Table S6). Without MMS, L415I, which was not observed in any other background, was found. Unexpectedly, 60% of Rif^R^-conferring mutations were mapped outside *rpoB* (*P* < 0.00001). This suggests that DinG contributes to maintaining mutational specificity, possibly through affecting the resolution of RTCs. However, after MMS exposure, the spectrum again collapsed to H482Y, as in Δ*rarA* or Δ*polA* cells. In Δ*mfd* cells, all mutations mapped to Cluster I. The S487L, also seen in Δ*disA*, appeared following MMS treatment.

Overall, 85% of mutations were transitions (transition:transversion ratio of ∼6:1) (Fig. 6E). Transitions accounted for 78.5% of mutations in the absence of MMS, and this percentage increased to 92.6% after MMS exposure. Most were C → T substitutions (65%), largely reflecting H482Y enrichment. However, we do not favour the interpretation that this itself reflects a general preference for C → T over other transitions. Instead, we support the view that it results from enrichment of H482Y, which likely confers the highest fitness, since other C → T transition (S487L) is less frequent than A → G transitions (Q469R or H482R). Therefore, bias in the Rif^R^-conferring mutation mechanism contributes significantly to the observed outcome. Even when H482Y was excluded (Supplementary Fig. S2A), transitions remained predominant (68.6% of events), increasing from 60% [−MMS] to 80% [+MMS], however they were nearly 100% A → G transitions. T → C or G → A transitions were not observed. These results indicate a very strong intrinsic transition bias.

Among transversions, A → C mutations are statistically overrepresented (52% of transversions, *P* < 0.00001), though all transversion types (G → T, C → A, A → T, and A → C) decreased after MMS treatment, leading to an enrichment of A → C mutations (75% of transversions, *P* < 0.00001) (Fig. 6E). T → A, G → C, C → G, and T → G transversions were not observed.

Strain-specific analyses revealed that Δ*polY1*, Δ*polY2*, Δ*mutSL*, Δ*disA*, Δ*dinG*, Δ*recD2*, and Δ*rarA* exhibited predominantly or exclusively transitions, Δ*recA* showed unusually elevated A → C transversions. wt, Δ*polA*, and Δ*mfd* displayed more mixed distributions (Fig. 6E). MMS significantly increased the transition:transversion ratio in wt, Δ*polA*, Δ*dinG*, and Δ*recA* strains (*P* ∼0.008), consistent with H482Y enrichment. If, as previously done, H482Y is excluded from the analysis, Δ*polY1*, Δ*polY2*, Δ*mutSL*, Δ*disA*, Δ*recD2*, and Δ*rarA* still show nearly all transitions, and Δ*mfd* still displays a more mixed distribution (Supplementary Fig. S2A). However, wt and Δ*polA* exhibit a high transversion rate, Δ*dinG* shows a more balanced distribution, and Δ*recA* shows only transversions. In this case, the transition:transversion ratio decreases to zero in the Δ*polA*, Δ*dinG*, Δ*rarA*, and Δ*recA* strains following MMS treatment.

To further dissect the role of one of the major determinants of mutagenesis, Δ*recA* was analysed in Δ*polY1*, Δ*polY2*, Δ*polA*, and Δ*mutSL* backgrounds. In the absence of MMS, mutation spectra of Δ*recA* Δ*polY1*, Δ*recA* Δ*polY2*, and Δ*recA* Δ*mutSL* clearly resembled those of their respective Δ*polY1*, Δ*polY2*, and Δ*mutSL* parental strains, both in terms of the specific mutations detected and the transition:transversion ratio (Supplementary Fig. S2B and C, and Supplementary Table S6). Unexpectedly, a rare H482D (C → G) mutation was detected in Δ*recA* Δ*polY2*. The Δ*recA* Δ*polA* double mutant resembled Δ*polA* rather than Δ*recA*, though Q469 mutations were predominantly Q469R transitions, increasing the transition:transversion ratio relative to Δ*polA*, in which transversions at Q469 were mostly found. Notably, H482P—the hallmark Δ*recA* mutation—was absent in all double mutants.

Altogether, these distinct mutational spectra demonstrate that: (i) in the absence of MMR or TLS, different mutations classes accumulate; and (ii) RecA, DisA, RarA, RecD2, DinG, and Mfd regulate TLS outcome, with RecA playing a central role in shaping the mutational landscape, consistent with earlier observations (Fig. 3C).

## Conclusions

The choice between error-free and error-prone DDT sub-pathways is critical for cell survival and therefore must be tightly regulated. This study provides the first systematic analysis of the regulation of DNA lesion bypass following endogenous and exogenous threats in exponentially growing *B. subtilis* cells lacking the SPβ-encoded TLS DNAP. Our work identifies key accessory proteins that may govern pathway choice at stalled replication forks and modulate mutagenesis levels, characterising their interplay with TLS DNAPs, with a particular emphasis on PolA.

In the absence of exogenous DNA damage, TLS appears to involve a two-step mechanism mediated by bipartite DNAPs, in which PolA assists the error-prone activities of PolY1 or PolY2 [22]. Following MMS treatment, however, a fraction of targeted mutagenesis may occur via PolA-independent mechanisms (Fig. 2B). Whether PolY1 or PolY2 can independently compete with transiently disassembled PolC to bypass lesions remains unknown. Moreover, the base misincorporation preferences of TLS DNAPs have not yet been characterised. Consequently, it remains unclear whether a TLS DNAP preferentially generates specific mismatches during base misincorporation or extends particular terminal mismatches generated by PolC. In both PolA-dependent and -independent mechanisms, PolC proofreading and post-replicative MMR, facilitated by DnaN-mediated recruitment of MutSL, likely act to correct misincorporated nucleotides.

In bacteria, RecA plays a central role in DDT at stalled forks and in SOS induction. In *E. coli*, inactivation of *recA* results in a non-mutable phenotype, whereas inactivation of *polA* does not affect mutagenesis [8, 15]. Specifically, the RecA*_Eco_* nucleoprotein filament promotes error-prone DDT through SOS induction and by facilitating assembly of the active Mut form of Pol V*_Eco_* (UmuD′_2_  *_Eco_*·UmuC*_Eco_*·RecA*_Eco_*·ATP). Once assembled, Pol V*_Eco_* Mut can function independently of a RecA*_Eco_* nucleoprotein filament, which is therefore only required for the assembly and activation of further Pol V*_Eco_* Mut complexes [41]. In contrast, our data suggest that RecA and PolA are key for both SOS-independent spontaneous and SOS-dependent MMS-induced mutagenesis in *B. subtilis* (Fig. 7). Indeed, RecA and PolA form foci at spontaneously or damage-induced stalled forks [20, 29] and physically interact with one another, as well as with TLS polymerases and several fork-associated factors shown here to influence TLS activity (Figs 1, 3, 4 and 5) [28]. It remains unclear whether a Mut form of PolY2 (PolY2·YqjX) exists.

RecA appears to play a pro-mutagenic role, likely by stimulating PolY1 activity and/or PolY2 activity and/or levels (Fig. 3C and D). In the simultaneous absence of RecA and PolA, PolY1 and/or PolY2 may catalyse lesion bypass in a PolA-independent manner (Fig. 3C): RecA may contribute with DnaN to load PolA at stalled forks [28, 30], and in the absence of both RecA and PolA, DnaN may instead recruit PolY1 and/or PolY2, which extend the nascent strand to bypass the lesion, fix the misincorporation, and subsequently hand off synthesis to PolC. Consistent with this, a distinct mutational landscape was observed in the absence of RecA (Fig. 6, Supplementary Fig. S2, and Supplementary Table S6). These phenotypes can be also partially rationalised by differences in SOS induction (Fig. 7). In the absence of RecA, SOS induction is abolished and mutagenesis decreases, as also observed upon inactivation of the RecA-positive modulator RarA, that decreases the formation of RecA filaments required for SOS induction (Fig.s 3C and 4B). Conversely, constitutive SOS induction in the absence of LexA or inactivation of the RecA-negative modulator RecD2, which increases RecA filament stability, resulted in elevated mutagenesis, an effect that is partially suppressed by PolA inactivation, though SOS-independent mutagenesis still occurs in the absence of both RecA and PolA (Figs 3D and 4D). Moreover, inactivation of both RarA and RecD2 produced an intermediate phenotype (Fig. 4E). Consistent with that, in the absence of LexA, survival to MMS is enhanced, while in the absence of SOS induction, it is reduced (Fig. 3B). However, the mutagenic phenotype observed in the *lexA*(Ind^−^) background cannot be fully explained by SOS regulation alone, indicating that additional, as-yet-unidentified factors likely contribute to mutagenesis control.

Beyond RecA, we demonstrate that several fork-associated proteins may modulate the balance between error-free and error-prone DDT, and thus mutagenesis (Fig. 7). We first showed that these proteins physically interact with PolA and/or RecA (Fig. 1), and alter the survival to MMS of mutants in TLS DNAPs to different extent (Figs 4 and 5). We then confirm the pro-mutagenic role of DisA, RarA, and DinG, and the anti-mutagenic role of RecD2 and Mfd. Moreover, we described the genetic interactions of these proteins with TLS DNAPs to up- or downregulate error-prone DDT pathways (Figs 4 and 5): (i) DisA may promote both PolA-dependent or independent PolY1-mediated spontaneous mutagenesis and PolY2-mediated MMS-induced mutagenesis, in addition to its potential influence on mutagenesis through modulation of RecA activity; (ii) RarA likely enhances PolA-dependent PolY2 activity, in addition to its plausible influence in mutagenesis through RecA filament dynamics and SOS response regulation; (iii) RecD2 may associate with PolA and inhibit PolY1–PolA-mediated TLS, while also influencing mutagenesis via RecA filament destabilisation and SOS response regulation; (iv) DinG may promote error-prone DDT through functional interaction with PolA and/or RecA; (v) Mfd may downregulate PolY1 activity and facilitate PolA loading upon at RTCs; (vi) in the absence of MMR, DinG and Mfd strongly promote error-prone DDT sub-pathways; and (viii) DisA, RarA, RecD2, DinG, and Mfd affect, albeit to different extents, the mutational landscape of the cell.

Collectively, our findings establish a regulatory network centred on PolA and RecA that integrates multiple accessory factors to fine-tune pathway choice between error-free and error-prone lesion bypass (Fig. 7). This work lays the foundation for future mechanistic studies dissecting how RecA, DisA, RarA, RecD2, DinG, and Mfd modulate polymerase recruitment, activity, and hand-off at stalled forks. Future studies should also investigate responses to other types of DNA damage. Elucidating these mechanisms will be essential for understanding bacterial genome evolution, speciation, and the emergence of antibiotic resistance, processes in which mutagenesis plays a pivotal role.

## Supplementary Material

gkag673_Supplemental_Files

## Data Availability

All data required to evaluate the conclusions of this study are included in the main text and/or the Supplementary materials. Raw data and materials used in this study are available from the authors upon request.

## References

[1] LeClerc JE, Li B, Payne WL et al. High mutation frequencies among *Escherichia coli* and *Salmonella* pathogens. Science. 1996;274:1208–11. https://doi.org/science.274.5290.1208 8895473 10.1126/science.274.5290.1208

[2] Rosenberg SM . Evolving responsively: adaptive mutation. Nat Rev Genet. 2001;2:504–15. 10.1038/35080556 11433357

[3] Andersson DI, Hughes D. Microbiological effects of sublethal levels of antibiotics. Nat Rev Microbiol. 2014;12:465–78. 10.1038/nrmicro3270 24861036

[4] Sanjanwala B, Ganesan AT. Genetic structure and domains of DNA polymerase III of *Bacillus subtilis*. Molec Gen Genet. 1991;226:467–72. 10.1007/BF00260660 1840638

[5] Sanders GM, Dallmann HG, McHenry CS. Reconstitution of the *B. subtilis* replisome with 13 proteins including two distinct replicases. Mol Cell. 2010;37:273–81. 10.1016/j.molcel.2009.12.025 20122408

[6] McHenry CS . Bacterial replicases and related polymerases. Curr Opin Chem Biol. 2011;15:587–94. 10.1016/j.cbpa.2011.07.018 21855395 PMC3190588

[7] Seco EM, Ayora S. *Bacillus subtilis* DNA polymerases, PolC and DnaE, are required for both leading and lagging strand synthesis in SPP1 origin-dependent DNA replication. Nucleic Acids Res. 2017;45:8302–13. 10.1093/nar/gkx493 28575448 PMC5737612

[8] Jarosz DF, Beuning PJ, Cohen SE et al. Y-family DNA polymerases in *Escherichia coli*. Trends Microbiol. 2007;15:70–7. 10.1016/j.tim.2006.12.004. 17207624

[9] Goodman MF, Woodgate R. Translesion DNA polymerases. Cold Spring Harb Perspect Biol. 2013;5:a010363. 10.1101/cshperspect.a010363 23838442 PMC3783050

[10] Fujii S, Fuchs RP. A comprehensive view of translesion synthesis in *Escherichia coli*. Microbiol Mol Biol Rev. 2020;84:e00002–20. 10.1128/MMBR.00002-20 32554755 PMC7307797

[11] Timinskas K, Kazlauskas D, Timinskas A et al. Diversity and distribution of bacterial DNA polymerases. Nucleic Acids Res. 2026;54:gkag133. 10.1093/nar/gkag133 41728957 PMC12926913

[12] O’Neal LG, Drucker MN, Lai NK et al. The *B. subtilis* replicative polymerases bind the sliding clamp with different strengths to tune their activity in DNA replication. Nucleic Acids Res. 2025;53:gkaf721. 10.1093/nar/gkaf721 40737090 PMC12309361

[13] Torres R, Carrasco B, Ayora S et al. Mechanisms of chromosomal DNA replication in *Escherichia coli* and *Bacillus subtilis*. FEMS Microbiol Rev. 2026;50:fuag021. 10.1093/femsre/fuag021 42133467 PMC13220808

[14] Le Chatelier E, Becherel OJ, d’Alencon E et al. Involvement of DnaE, the second replicative DNA polymerase from *Bacillus subtilis*, in DNA mutagenesis. J Biol Chem. 2004;279:1757–67. 10.1074/jbc.M310719200 14593098

[15] Friedberg EC, Walker GC, Siede W et al. DNA repair and mutagenesis, 2nd edn. Washington DC: ASM Press, 2006.

[16] Carrasco B, Torres R, Álamo M-D et al. Processing of stalled replication forks in *Bacillus subtilis*. FEMS Microbiol Rev. 2024;48:fuad065. 10.1093/femsre/fuad065 38052445 PMC10804225

[17] Mangiameli SM, Merrikh CN, Wiggins PA et al. Transcription leads to pervasive replisome instability in bacteria. eLife. 2017;6:e19848. 10.7554/eLife.19848 28092263 PMC5305214

[18] Huang D, Johnson AE, Sim BS et al. The *in vivo* measurement of replication fork velocity and pausing by lag-time analysis. Nat Commun. 2023;14:1762. 10.1038/s41467-023-37456-2 36997519 PMC10063678

[19] Torres R, Carrasco B, Ayora S et al. Hallmarks of DNA replication stress in *Escherichia coli* and *Bacillus subtilis*. FEMS Microbiol Rev. 2025;49:fuaf041. 10.1093/femsre/fuaf041 40874739 PMC12448304

[20] Simmons LA, Grossman AD, Walker GC. Replication is required for the RecA localization response to DNA damage in *Bacillus subtilis*. Proc Natl Acad Sci USA. 2007;104:1360–5. 10.1073/pnas.0607123104 17229847 PMC1783139

[21] Lenhart JS, Brandes ER, Schroeder JW et al. RecO and RecR are necessary for Reca loading in response to DNA damage and replication fork stress. J Bacteriol. 2014;196:2851–60. 10.1128/JB.01494-14 24891441 PMC4135682

[22] Duigou S, Ehrlich SD, Noirot P et al. DNA polymerase I acts in translesion synthesis mediated by the Y-polymerases in *Bacillus subtilis*. Mol Microbiol. 2005;57:678–90. 10.1111/j.1365-2958.2005.04725.x 16045613

[23] Walsh BW, Bolz SA, Wessel SR et al. RecD2 helicase limits replication fork stress in *Bacillus subtilis*. J Bacteriol. 2014;196:1359–68. 10.1128/JB.01475-13 24443534 PMC3993351

[24] Carrasco B, Seco EM, López-Sanz M et al. *Bacillus subtilis* RarA modulates replication restart. Nucleic Acids Res. 2018;46:7206–20. 10.1093/nar/gky541 29947798 PMC6101539

[25] Romero H, Rosch TC, Hernández-Tamayo R et al. Single molecule tracking reveals functions for RarA at replication forks but also independently from replication during DNA repair in *Bacillus subtilis*. Sci Rep. 2019;9:1997. 10.1038/s41598-018-38289-6 30760776 PMC6374455

[26] Torres R, Carrasco B, Gándara C et al. *Bacillus subtilis* DisA regulates RecA-mediated DNA strand exchange. Nucleic Acids Res. 2019;47:5141–54. 10.1093/nar/gkz219 30916351 PMC6547438

[27] Ramos C, Hernández-Tamayo R, López-Sanz M et al. The RecD2 helicase balances RecA activities. Nucleic Acids Res. 2022;50:3432–44. 10.1093/nar/gkac131 35234892 PMC8989531

[28] Carrasco B, Torres R, López-Sanz M et al. *Bacillus subtilis* DinG 3'⟶5' exo(ribo)nuclease: a helpmate to mitigate replication stress. Int J Mol Sci. 2025;26:9681. 10.3390/ijms26199681 41096947 PMC12525004

[29] Hinrichs R, Graumann PL. Visual evidence for the recruitment of four enzymes with RNase Activity to the *Bacillus subtilis* Replication Forks. Cells. 2024;13:1381. 10.3390/cells13161381 39195267 PMC11352351

[30] Marrin ME, Foster MR, Santana CM et al. The translesion polymerase Pol Y1 is a constitutive component of the *B. subtilis* replication machinery. Nucleic Acids Res. 2024;52:9613–29. 10.1093/nar/gkae637 39051562 PMC11381352

[31] Au N, Kuester-Schoeck E, Mandava V et al. Genetic composition of the *Bacillus subtilis* SOS system. J Bacteriol. 2005;187:7655–66. 10.1128/JB.187.22.7655-7666.2005 16267290 PMC1280312

[32] Carvajal-García J, Samadpour AN, Viera H et al. Oxidative stress drives mutagenesis through transcription-coupled repair in bacteria. Proc Natl Acad Sci USA. 2023;120:e2300761120. 10.1073/pnas.2300761120 37364106 PMC10318952

[33] Raguse M, Torres R, Seco EM et al. *Bacillus subtilis* DisA helps to circumvent replicative stress during spore revival. DNA Repair. 2017;59:57–68. 10.1016/j.dnarep.2017.09.006 28961460

[34] Liao Y, Schroeder JW, Gao B et al. Single-molecule motions and interactions in live cells reveal target search dynamics in mismatch repair. Proc Natl Acad Sci USA. 2015;112:E6898–6906. 10.1073/pnas.1507386112 26575623 PMC4687589

[35] Pillon MC, Lorenowicz JJ, Uckelmann M et al. Structure of the endonuclease domain of MutL: unlicensed to cut. Mol Cell. 2010;39:145–51. 10.1016/j.molcel.2010.06.027 20603082 PMC2933357

[36] Pillon MC, Babu VM, Randall JR et al. The sliding clamp tethers the endonuclease domain of MutL to DNA. Nucleic Acids Res. 2015;43:10746–59. 10.1093/nar/gkv918 26384423 PMC4678855

[37] Lenhart JS, Pillon MC, Guarne A et al. Mismatch repair in Gram-positive bacteria. Res Microbiol. 2016;167:4–12. 10.1016/j.resmic.2015.08.006 26343983

[38] Robinson A, McDonald JP, Caldas VE et al. Regulation of mutagenic DNA Polymerase V activation in space and time. PLoS Genet. 2015;11:e1005482. 10.1371/journal.pgen.1005482 26317348 PMC4552617

[39] Henrikus SS, Wood EA, McDonald JP et al. DNA polymerase IV primarily operates outside of DNA replication forks in *Escherichia coli*. PLoS Genet. 2018;14:e1007161. 10.1371/journal.pgen.1007161 29351274 PMC5792023

[40] Kiefer JR, Mao C, Hansen CJ et al. Crystal structure of a thermostable *Bacillus* DNA polymerase I large fragment at 2.1 Å resolution. Structure. 1997;5:95–108. 10.1016/s0969-2126(97)00169-x 9016716

[41] Jiang Q, Karata K, Woodgate R et al. The active form of DNA polymerase V is UmuD′(2)C-RecA-ATP. Nature. 2009;460:359–63. 10.1038/nature08178 19606142 PMC2731490

[42] Patlan AG, Corona SU, Ayala-García VM et al. Non-canonical processing of DNA photodimers with *Bacillus subtilis* UV-endonuclease YwjD, 5'→3' exonuclease YpcP and low-fidelity DNA polymerases YqjH and YqjW. DNA Repair. 2018;70:1–9. 10.1016/j.dnarep.2018.07.007 30096406

[43] Duigou S, Ehrlich SD, Noirot P et al. Distinctive genetic features exhibited by the Y-family DNA polymerases in *Bacillus subtilis*. Mol Microbiol. 2004;54:439–51. 10.1111/j.1365-2958.2004.04259.x 15469515

[44] Valero-Rello A, López-Sanz M, Quevedo-Olmos A et al. Molecular mechanisms that contribute to horizontal transfer of plasmids by the bacteriophage SPP1. Front Microbiol. 2017;8:1816. 10.3389/fmicb.2017.01816 29018417 PMC5615212

[45] Karimova G, Pidoux J, Ullmann A et al. A bacterial two-hybrid system based on a reconstituted signal transduction pathway. Proc Natl Acad Sci USA. 1998;95:5752–6. 10.1073/pnas.95.10.5752 9576956 PMC20451

[46] Lindahl T, Wood RD. Quality control by DNA repair. Science. 1999;286:1897–905. 10.1126/science.286.5446.1897 10583946

[47] Sánchez H, Carrasco B, Cozar MC et al. *Bacillus subtilis* RecG branch migration translocase is required for DNA repair and chromosomal segregation. Mol Microbiol. 2007;65:920–35. 10.1111/j.1365-2958.2007.05835.x 17640277

[48] Cárdenas PP, Carrasco B, Defeu Soufo C et al. RecX facilitates homologous recombination by modulating RecA activities. PLoS Genet. 2012;8:e1003126. 10.1371/journal.pgen.1003126 23284295 PMC3527212

[49] Carrasco B, Serrano E, Martín-González A et al. *Bacillus subtilis* MutS modulates RecA-mediated DNA strand exchange between divergent DNA sequences. Front Microbiol. 2019;10:237. 10.3389/fmicb.2019.00237 30814990 PMC6382021

[50] Sedgwick B . Repairing DNA-methylation damage. Nat Rev Mol Cell Biol. 2004;5:148–57. 10.1038/nrm1312 15040447

[51] Ayora S, Rojo F, Ogasawara N et al. The Mfd protein of *Bacillus subtilis* 168 is involved in both transcription-coupled DNA repair and DNA recombination. J Mol Biol. 1996;256:301–18. 10.1006/jmbi.1996.0087 8594198

[52] Alonso JC, Tailor RH, Lüder G. Characterization of recombination-deficient mutants of *Bacillus subtilis*. J Bacteriol. 1988;170:3001–7. 10.1128/jb.170.7.3001-3007.1988 3133357 PMC211241

[53] Gándara C, Alonso JC. DisA and c-di-AMP act at the intersection between DNA-damage response and stress homeostasis in exponentially growing *Bacillus subtilis* cells. DNA Repair. 2015;27:1–8. 10.1016/j.dnarep.2014.12.007 25616256

[54] Torres R, Romero H, Rodríguez-Cerrato V et al. Interplay between *Bacillus subtilis* RecD2 and the RecG or RuvAB helicase in recombinational repair. DNA Repair. 2017;55:40–6. 10.1016/j.dnarep.2017.05.004 28527403

[55] Romero H, Torres R, Hernández-Tamayo R et al. *Bacillus subtilis* RarA acts at the interplay between replication and repair-by-recombination. DNA Repair. 2019;78:27–36. 10.1016/j.dnarep.2019.03.010 30954900

[56] Luria SE, Delbrück M. Mutations of bacteria from virus sensitivity to virus resistance. Genetics. 1943;28:491–511. 10.1093/genetics/28.6.491 17247100 PMC1209226

[57] Drake JW . A constant rate of spontaneous mutation in DNA-based microbes. Proc Natl Acad Sci USA. 1991;88:7160–4. 10.1073/pnas.88.16.7160 1831267 PMC52253

[58] Gassel M, Alonso JC. Expression of the *recE* gene during induction of the SOS response in *Bacillus subtilis* recombination-deficient strains. Mol Microbiol. 1989;3:1269–76. 10.1111/j.1365-2958.1989.tb00277.x 2507872

[59] Tanneur I, Dervyn E, Guerin C et al. The mutational landscape of *Bacillus subtilis* conditional hypermutators shows how proofreading skews DNA polymerase error rates. Nucleic Acids Res. 2025;53:gkaf147. 10.1093/nar/gkaf147 40057377 PMC11890065

[60] Cox MM . Motoring along with the bacterial RecA protein. Nat Rev Mol Cell Biol. 2007;8:127–38. 10.1038/nrm2099 17228330

[61] Bell JC, Kowalczykowski SC. RecA: regulation and mechanism of a molecular search engine. Trends Biochem Sci. 2016;41:491–507. 10.1016/j.tibs.2016.04.002 27156117 PMC4892382

[62] Schroeder JW, Hurto RL, Randall JR et al. RNase H genes cause distinct impacts on RNA:DNA hybrid formation and mutagenesis genome wide. Sci Adv. 2023;9:eadi5945. 10.1126/sciadv.adi5945 37494439 PMC10371020

[63] Vlasic I, Mertens R, Seco EM et al. *Bacillus subtilis* RecA and its accessory factors, RecF, RecO, RecR and RecX, are required for spore resistance to DNA double-strand break. Nucleic Acids Res. 2014;42:2295–307. 10.1093/nar/gkt1194 24285298 PMC3936729

[64] Romero H, Serrano E, Hernández-Tamayo R et al. *Bacillus subtilis* RarA acts as a positive Reca accessory protein. Front Microbiol. 2020;11:92. 10.3389/fmicb.2020.00092 32117122 PMC7031210

[65] Lowder FC, Kendal AH, Simmons LA. DNA polymerase I is an efficient reverse transcriptase that mediates RNA-templated DNA repair synthesis. bioRxiv, 10.64898/2025.12.22.696026, 23 December 2025, preprint: not peer reviewed.

[66] Modrich P . DNA mismatch correction. Annu Rev Biochem. 1987;56:435–66. 10.1146/annurev.bi.56.070187.002251 3304141

[67] Modrich P . Mechanisms in *E. coli* and human mismatch repair (Nobel Lecture). Angew Chem Int Ed. 2016;55:8490–501. 10.1002/anie.201601412 PMC5193110 27198632

[68] Costes A, Lecointe F, McGovern S et al. The C-terminal domain of the bacterial SSB protein acts as a DNA maintenance hub at active chromosome replication forks. PLoS Genet. 2010;6:e1001238. 10.1371/journal.pgen.1001238 21170359 PMC3000357

[69] Yoshimura A, Seki M, Enomoto T. The role of WRNIP1 in genome maintenance. Cell Cycle. 2017;16:515–21. 10.1080/15384101.2017.1282585 28118071 PMC5384577

[70] Hormeño S, Ramos C, Mendia-García J et al. Bacterial RecD2 is a processive single-stranded DNA translocase with strand-switching capacity at DNA forks. Nucleic Acids Res. 2025;53:gkaf459. 10.1093/nar/gkaf459 40479711 PMC12143592

[71] Bejerano-Sagie M, Oppenheimer-Shaanan Y, Berlatzky I et al. A checkpoint protein that scans the chromosome for damage at the start of sporulation in *Bacillus subtilis*. Cell. 2006;125:679–90. 10.1016/j.cell.2006.03.039 16713562

[72] Torres R, Serrano E, Tramm K et al. *Bacillus subtilis* RadA/Sms contributes to chromosomal transformation and DNA repair in concert with RecA and circumvents replicative stress in concert with DisA. DNA Repair. 2019;77:45–57. 10.1016/j.dnarep.2019.03.002 30877841

[73] Selby CP, Lindsey-Boltz LA, Li W et al. Molecular mechanisms of transcription-coupled repair. Annu Rev Biochem. 2023;92:115–44. 10.1146/annurev-biochem-041522-034232 37001137

[74] Campbell EA, Korzheva N, Mustaev A et al. Structural mechanism for rifampicin inhibition of bacterial RNA polymerase. Cell. 2001;104:901–12. 10.1016/s0092-8674(01)00286-0 11290327

[75] Ingham CJ, Furneaux PA. Mutations in the β subunit of the *Bacillus subtilis* RNA polymerase that confer both rifampicin resistance and hypersensitivity to NusG. Microbiology (Reading). 2000;146:3041–9. 10.1099/00221287-146-12-3041 11101662

