# Supplementary material for: Genetic dissection of DNA damage tolerance in Bacillus subtilis: RecA and recombination functions regulate translesion synthesis
Source: Nucleic Acids Res. 2026 Jul 6;54(13):gkag673. doi: 10.1093/nar/gkag673 (PMC13335488; doi:10.1093/nar/gkag673)
Supplement: gkag673_Supplemental_Files [file gkag673_supplemental_files.zip › Torres and Alonso Supplementary Materials File 1.pdf]

Genetic dissection of DNA damage tolerance in *Bacillus subtilis*: RecA and recombination functions regulate translesion synthesis

Rubén Torres* and Juan C. Alonso*

Department of Microbial Biotechnology, Centro Nacional de Biotecnología (CNB-CSIC), 3 Darwin Str, 28049, Madrid, Spain.

Table S1. *Bacillus subtilis* strains used in this study.

Strain	Relevant genotype	Source
BG214	parental strain (*trpCE metA*5 *amyE*1 *ytsJ*1 *rsbV*37 *xre*1 *xkdA*1 $att^{SP\beta}$ att^{ICEBs1})	Laboratory strain
BG905	+ Δ*polY1*	(1)
BG907	+ Δ*polY2*	(1)
BG1993	+ Δ*polA*	(2)
BG1393	+ Δ*mutSL*	(3)
BG1501	+ Δ*polY1* Δ*polY2*	(1)
BG1997	+ Δ*polY1* Δ*polA*	This work
BG1999	+ Δ*polY2* Δ*polA*	This work
BG1873	+ Δ*recA*	(3)
BG2065	+ Δ*recA* Δ*polY1*	This work
BG2057	+ Δ*recA* Δ*polY2*	This work
BG2063	+ Δ*recA* Δ*polA*	This work
BG2059	+ Δ*recA* Δ*mutSL*	This work
BG1047	+ Δ*lexA*	(4)
BG2027	+ Δ*lexA* Δ*polA*	This work
BG1519	+ Δ*upp lexA*(Ind^-)	This work
BG2029	+ Δ*upp lexA*(Ind^-) Δ*polA*	This work
BG1221	+ Δ*disA*	(5)
BG1513	+ Δ*disA* Δ*polY1*	(1)
BG1497	+ Δ*disA* Δ*polY2*	(1)
BG2007	+ Δ*disA* Δ*polA*	This work
BG2033	+ Δ*disA* Δ*mutSL*	This work
BG1067	+ Δ*rarA*	(6)
BG1401	+ Δ*rarA* Δ*polY1*	(7)
BG1403	+ Δ*rarA* Δ*polY2*	(7)
BG2001	+ Δ*rarA* Δ*polA*	This work
BG2023	+ Δ*rarA* Δ*mutSL*	This work
BG1455	+ Δ*recD2*	(8)
BG1511	+ Δ*recD2* Δ*polY1*	This work
BG1495	+ Δ*recD2* Δ*polY2*	This work
BG2003	+ Δ*recD2* Δ*polA*	This work
BG2021	+ Δ*recD2* Δ*mutSL*	This work
BG1421	+ Δ*recD2* Δ*rarA*	This work
BG2055	+ Δ*recD2* Δ*rarA* Δ*polA*	This work
BG1605	+ Δ*dinG*	(8)
BG1987	+ Δ*dinG* Δ*polY1*	(2)
BG1991	+ Δ*dinG* Δ*polY2*	(2)
BG1995	+ Δ*dinG* Δ*polA*	(2)
BG2031	+ Δ*dinG* Δ*mutSL*	This work
BG339	+ Δ*mfd*	(9)
BG1911	+ Δ*mfd* Δ*polY1*	This work
BG1913	+ Δ*mfd* Δ*polY2*	This work
BG2005	+ Δ*mfd* Δ*polA*	This work
BG2025	+ Δ*mfd* Δ*mutSL*	This work

Table S2. Survival and mutation frequency data.

Strain	LD_{90} to MMS (mM)[a]	Mutation frequency[b] - MMS	Mutation frequency[b] 3 mM MMS
wt	1.8 ± 0.12	$2.7 \times 10^{-9} \pm 8.2 \times 10^{-10}$	$5.9 \times 10^{-9} \pm 8.0 \times 10^{-10}$
Δ*polY1*	1.6 ± 0.11	$1.8 \times 10^{-9} \pm 5.4 \times 10^{-10}$	$3.7 \times 10^{-9} \pm 4.6 \times 10^{-10}$
Δ*polY2*	1.5 ± 0.09	$1.6 \times 10^{-9} \pm 6.7 \times 10^{-10}$	$1.9 \times 10^{-9} \pm 7.4 \times 10^{-10}$
Δ*polA*	1.0 ± 0.17	$4.4 \times 10^{-10} \pm 1.5 \times 10^{-10}$	$1.9 \times 10^{-9} \pm 8.5 \times 10^{-10}$
Δ*mutSL*	2.4 ± 0.06	$3.1 \times 10^{-7} \pm 1.3 \times 10^{-7}$	$8.9 \times 10^{-7} \pm 5.4 \times 10^{-10}$
Δ*polY1* Δ*polY2*	1.4 ± 0.12	$3.1 \times 10^{-10} \pm 1.5 \times 10^{-10}$	$6.4 \times 10^{-10} \pm 2.5 \times 10^{-10}$
Δ*polY1* Δ*polA*	0.9 ± 0.17	$3.9 \times 10^{-10} \pm 9.2 \times 10^{-11}$	$9.3 \times 10^{-10} \pm 1.3 \times 10^{-10}$
Δ*polY2* Δ*polA*	1.2 ± 0.93	$3.4 \times 10^{-10} \pm 3.0 \times 10^{-11}$	$1.3 \times 10^{-9} \pm 2.0 \times 10^{-10}$
Δ*recA*	0.038 ± 0.0019	$6.2 \times 10^{-10} \pm 1.6 \times 10^{-10}$	$7.3 \times 10^{-10} \pm 1.2 \times 10^{-11}$
Δ*recA* Δ*polY1*	0.025 ± 0.0027	$8.4 \times 10^{-10} \pm 3.8 \times 10^{-10}$	$8.6 \times 10^{-10} \pm 4.6 \times 10^{-10}$
Δ*recA* Δ*polY2*	0.026 ± 0.0013	$3.9 \times 10^{-10} \pm 3.2 \times 10^{-11}$	$3.3 \times 10^{-10} \pm 4.9 \times 10^{-11}$
Δ*recA* Δ*polA*	0.016 ± 0.0017	$2.2 \times 10^{-9} \pm 7.3 \times 10^{-10}$	$5.0 \times 10^{-9} \pm 1.8 \times 10^{-9}$
Δ*recA* Δ*mutSL*	0.034 ± 0.0015	$5.0 \times 10^{-7} \pm 2.6 \times 10^{-7}$	$6.6 \times 10^{-7} \pm 3.0 \times 10^{-9}$
Δ*lexA*	2.1 ± 0.12	$4.6 \times 10^{-8} \pm 2.4 \times 10^{-8}$	$5.4 \times 10^{-8} \pm 2.8 \times 10^{-8}$
Δ*lexA* Δ*polA*	1.1 ± 0.08	$7.0 \times 10^{-10} \pm 2.3 \times 10^{-10}$	$3.3 \times 10^{-9} \pm 8.9 \times 10^{-10}$
lexA(Ind^-)	1.2 ± 0.07	$4.0 \times 10^{-8} \pm 2.8 \times 10^{-8}$	$5.5 \times 10^{-9} \pm 9.5 \times 10^{-10}$
lexA(Ind^-) Δ*polA*	0.3 ± 0.11	$3.7 \times 10^{-9} \pm 3.0 \times 10^{-10}$	$1.2 \times 10^{-9} \pm 6.5 \times 10^{-10}$
Δ*disA*	1.7 ± 0.20	$7.2 \times 10^{-10} \pm 2.5 \times 10^{-10}$	$2.9 \times 10^{-9} \pm 5.0 \times 10^{-10}$
Δ*disA* Δ*polY1*	1.7 ± 0.09	$1.9 \times 10^{-9} \pm 7.6 \times 10^{-10}$	$2.5 \times 10^{-9} \pm 6.6 \times 10^{-10}$
Δ*disA* Δ*polY2*	1.3 ± 0.10	$5.7 \times 10^{-10} \pm 1.7 \times 10^{-10}$	$1.4 \times 10^{-9} \pm 5.0 \times 10^{-10}$
Δ*disA* Δ*polA*	0.6 ± 0.15	$4.1 \times 10^{-10} \pm 1.1 \times 10^{-10}$	$8.9 \times 10^{-10} \pm 6.9 \times 10^{-10}$
Δ*disA* Δ*mutSL*	1.5 ± 0.12	$3.1 \times 10^{-7} \pm 1.1 \times 10^{-7}$	$3.8 \times 10^{-7} \pm 1.3 \times 10^{-7}$
Δ*rarA*	1.9 ± 0.19	$6.7 \times 10^{-10} \pm 1.5 \times 10^{-10}$	$2.2 \times 10^{-9} \pm 8.6 \times 10^{-10}$
Δ*rarA* Δ*polY1*	1.7 ± 0.25	$1.4 \times 10^{-10} \pm 9.5 \times 10^{-11}$	$8.2 \times 10^{-10} \pm 2.6 \times 10^{-10}$
Δ*rarA* Δ*polY2*	1.8 ± 0.12	$1.9 \times 10^{-9} \pm 7.7 \times 10^{-10}$	$2.6 \times 10^{-9} \pm 8.6 \times 10^{-10}$
Δ*rarA* Δ*polA*	1.1 ± 0.21	$1.2 \times 10^{-10} \pm 6.9 \times 10^{-11}$	$3.3 \times 10^{-10} \pm 1.3 \times 10^{-10}$
Δ*rarA* Δ*mutSL*	2.4 ± 0.19	$4.8 \times 10^{-7} \pm 3.2 \times 10^{-7}$	$7.2 \times 10^{-7} \pm 2.3 \times 10^{-7}$
Δ*recD2*	1.3 ± 0.12	$1.2 \times 10^{-8} \pm 3.7 \times 10^{-9}$	$2.3 \times 10^{-8} \pm 6.1 \times 10^{-9}$
Δ*recD2* Δ*polY1*	1.2 ± 0.14	$1.7 \times 10^{-9} \pm 1.4 \times 10^{-9}$	$1.2 \times 10^{-8} \pm 3.6 \times 10^{-9}$
Δ*recD2* Δ*polY2*	1.5 ± 0.13	$9.3 \times 10^{-9} \pm 2.5 \times 10^{-9}$	$1.6 \times 10^{-8} \pm 4.4 \times 10^{-9}$
Δ*recD2* Δ*polA*	0.2 ± 0.16	$9.0 \times 10^{-10} \pm 3.4 \times 10^{-10}$	$5.7 \times 10^{-9} \pm 4.1 \times 10^{-9}$
Δ*recD2* Δ*mutSL*	1.3 ± 0.19	$4.7 \times 10^{-7} \pm 1.1 \times 10^{-7}$	$5.4 \times 10^{-7} \pm 1.3 \times 10^{-7}$
Δ*recD2* Δ*rarA*	1.7 ± 0.18	$6.9 \times 10^{-9} \pm 5.9 \times 10^{-9}$	$1.2 \times 10^{-8} \pm 7.9 \times 10^{-9}$
Δ*recD2* Δ*rarA* Δ*polA*	1.2 ± 0.22	$1.3 \times 10^{-9} \pm 1.1 \times 10^{-10}$	$3.6 \times 10^{-9} \pm 8.1 \times 10^{-10}$
Δ*dinG*	1.7 ± 0.11	$8.1 \times 10^{-10} \pm 3.5 \times 10^{-10}$	$2.8 \times 10^{-9} \pm 7.5 \times 10^{-10}$
Δ*dinG* Δ*polY1*	1.4 ± 0.14	$9.3 \times 10^{-10} \pm 4.1 \times 10^{-10}$	$2.5 \times 10^{-9} \pm 6.5 \times 10^{-10}$
Δ*dinG* Δ*polY2*	1.8 ± 0.13	$7.5 \times 10^{-10} \pm 3.1 \times 10^{-10}$	$1.8 \times 10^{-9} \pm 6.7 \times 10^{-10}$
Δ*dinG* Δ*polA*	1.2 ± 0.18	$4.5 \times 10^{-9} \pm 1.5 \times 10^{-10}$	$2.0 \times 10^{-9} \pm 6.8 \times 10^{-10}$
Δ*dinG* Δ*mutSL*	2.8 ± 0.11	$4.8 \times 10^{-7} \pm 1.3 \times 10^{-8}$	$4.7 \times 10^{-8} \pm 1.7 \times 10^{-8}$
Δ*mfd*	1.7 ± 0.13	$3.7 \times 10^{-9} \pm 4.7 \times 10^{-10}$	$9.6 \times 10^{-9} \pm 2.1 \times 10^{-9}$
Δ*mfd* Δ*polY1*	1.3 ± 0.19	$2.4 \times 10^{-9} \pm 1.9 \times 10^{-9}$	$3.7 \times 10^{-9} \pm 2.8 \times 10^{-9}$
Δ*mfd* Δ*polY2*	1.7 ± 0.15	$1.6 \times 10^{-8} \pm 2.3 \times 10^{-9}$	$2.8 \times 10^{-8} \pm 1.1 \times 10^{-9}$
Δ*mfd* Δ*polA*	1.1 ± 0.13	$2.2 \times 10^{-10} \pm 1.9 \times 10^{-11}$	$6.4 \times 10^{-10} \pm 2.5 \times 10^{-10}$
Δ*mfd* Δ*mutSL*	1.8 ± 0.21	$5.7 \times 10^{-8} \pm 1.6 \times 10^{-8}$	$5.4 \times 10^{-8} \pm 1.3 \times 10^{-8}$

[a]The lethal dose that kills 90% of the bacterial population (LD_{90}) values following chronic exposure to MMS (0.5-3.0 mM) is shown. Data represent the mean ± SD from at least three independent experiments.
[b]Spontaneous and MMS-induced mutagenesis in TLS DNAPs or MMR mutants in the absence of functions regulating DDT. Luria-Delbruck fluctuation analyses were performed by growing the indicated strains to OD_{560}= 0.8 in LB medium. A 10 ml aliquot of each culture was exposed to 3 mM MMS for 15 min. Cultures were then plated on LB or LB supplemented with rifampicin, and mutation frequencies were calculated as Rif^R CFUs normalised to total CFUs obtained on LB from untreated cells. Data represent the mean ± SD from at least five independent experiments.

Table S3. Statistical analysis of LD_{90} values following chronic exposure to MMS (*P* values[a] for pairwise *t*-test comparisons).

	wt	Δ*polY1*	Δ*polY2*	Δ*polA*	Δ*mutSL*	Δ*recA*	Δ*lexA*	*lexA*(Ind^{-})	Δ*disA*	Δ*rarA*	Δ*recD2*	Δ*recD2* Δ*rarA*	Δ*dinG*	Δ*mfd*
Δ*polY1*	0.100436													
Δ*polY2*	0.141552	0.280774												
Δ*polA*	<0.000001	0.006827	0.010367											
Δ*mutSL*	0.003576	0.000404	0.000126	0.000182										
Δ*polY1* Δ*polY2*	0.002068	0.100436	0.304155	0.029120	0.000219									
Δ*polY1* Δ*polA*	0.001698	0.003912		0.511109										
Δ*polY2* Δ*polA*	0.329896		0.607590	0.000162										
Δ*recA*	0.000014													
Δ*recA* Δ*polY1*	0.000014	0.000016				0.254161								
Δ*recA* Δ*polY2*	0.000014		0.000007			0.283415								
Δ*recA* Δ*polA*	0.000014			0.055714		0.004215								
Δ*recA* Δ*mutSL*	0.000014				<0.000001	0.458360								
Δ*lexA*	0.003759													
Δ*lexA* Δ*polA*	0.000212			0.409735			0.000280							
lexA(Ind^{-})	<0.000001													
lexA(Ind^{-}) Δ*polA*	<0.000001			0.003912				0.000294						
Δ*disA*	0.002466													
Δ*disA* Δ*polY1*	0.315889	0.293640							0.999999					
Δ*disA* Δ*polY2*	0.005176		0.057614						0.056278					
Δ*disA* Δ*polA*	0.000414			0.003781					0.000492					
Δ*disA* Δ*mutSL*	0.037590				0.000331				0.011664					
Δ*rarA*	0.038384													
Δ*rarA* Δ*polY1*	0.566098	0.560438								0.331848				
Δ*rarA* Δ*polY2*	0.999999		0.024155							0.483854				
Δ*rarA* Δ*polA*	0.007423			0.005563						0.008085				
Δ*rarA* Δ*mutSL*	0.009848				0.999999					0.032188				
Δ*recD2*	0.000596													
Δ*recD2* Δ*polY1*	0.004878	<0.000001									0.000600			
Δ*recD2* Δ*polY2*	0.042514		0.029899								0.000752			
Δ*recD2* Δ*polA*	0.000157			0.004039							0.000678			
Δ*recD2* Δ*mutSL*	0.018244				0.000684						0.216693			
Δ*recD2* Δ*rarA*	0.468196									0.256226	0.000265			
Δ*recD2* Δ*rarA* Δ*polA*	0.014295			0.280774						0.014021	0.527482	0.038152		
Δ*dinG*	0.347299													
Δ*dinG* Δ*polY1*	<0.000001	<0.000001											<0.000001	
Δ*dinG* Δ*polY2*	0.999999		0.028691										0.366624	
Δ*dinG* Δ*polA*	0.008624			0.234339									0.014788	
Δ*dinG* Δ*mutSL*	0.000442				0.005528								0.000255	
Δ*mfd*	0.383006													
Δ*mfd* Δ*polY1*	0.018244	0.001091												0.039573
Δ*mfd* Δ*polY2*	0.418216		0.003900											0.999999
Δ*mfd* Δ*polA*	0.002373			0.054637										0.004826
Δ*mfd* Δ*mutSL*	0.999999				0.008290									0.521778

[a]Purple, highly statistically significant difference (*P* <0.01); orange, statistically significant difference (*P* <0.05); black, not statistically significant difference (*P* >0.05).

Table S4. Statistical analysis of mutagenesis frequencies in the absence of MMS (*P* values[a] for pairwise *t*-test comparisons).

	wt	Δ*polY1*	Δ*polY2*	Δ*polA*	Δ*mutSL*	Δ*recA*	Δ*lexA*	*lexA*(Ind^{-})	Δ*disA*	Δ*rarA*	Δ*recD2*	Δ*recD2* Δ*rarA*	Δ*dinG*	Δ*mfd*
Δ*polY1*	0.011387													
Δ*polY2*	0.008019	0.653348												
Δ*polA*	<0.000001	0.000002	0.000074											
Δ*mutSL*	<0.000001	0.000003	0.000003	0.000003										
Δ*polY1* Δ*polY2*	<0.000001	<0.000001	0.000024	0.085857	0.000003									
Δ*polY1* Δ*polA*	0.000038	0.001322		0.881677										
Δ*polY2* Δ*polA*	<0.000001		0.000067	0.406798										
Δ*recA*	<0.000001													
Δ*recA* Δ*polY1*	0.000013	0.000639				0.134884								
Δ*recA* Δ*polY2*	<0.000001		0.000117			0.076312								
Δ*recA* Δ*polA*	0.176206			0.000003		0.000012								
Δ*recA* Δ*mutSL*	0.000027				0.073499	0.000011								
Δ*lexA*	<0.000001													
Δ*lexA* Δ*polA*	0.000003			0.012712			0.000036							
lexA(Ind^{-})	0.001143													
lexA(Ind^{-}) Δ*polA*	0.342714			0.004507				0.001489						
Δ*disA*	0.000003													
Δ*disA* Δ*polY1*	0.046489	0.682121							0.000425					
Δ*disA* Δ*polY2*	0.000001		0.000246						0.152336					
Δ*disA* Δ*polA*	<0.000001			0.747792					0.004011					
Δ*disA* Δ*mutSL*	<0.000001				0.976813				<0.000001					
Δ*rarA*	0.000002													
Δ*rarA* Δ*polY1*	<0.000001	<0.000001								<0.000001				
Δ*rarA* Δ*polY2*	0.046686		0.45208							0.000224				
Δ*rarA* Δ*polA*	<0.000001			0.000036						<0.000001				
Δ*rarA* Δ*mutSL*	0.000385				0.168238					0.00037				
Δ*recD2*	0.000002													
Δ*recD2* Δ*polY1*	0.084662	0.900819									<0.000001			
Δ*recD2* Δ*polY2*	0.000001		<0.000001								0.109649			
Δ*recD2* Δ*polA*	0.000016			0.001724							<0.000001			
Δ*recD2* Δ*mutSL*	<0.000001				0.013952						<0.000001			
Δ*recD2* Δ*rarA*	0.048659									0.005646	0.048992			
Δ*recD2* Δ*rarA* Δ*polA*	0.006429			0.044785						0.001378	<0.000001	0.011722		
Δ*dinG*	0.000009													
Δ*dinG* Δ*polY1*	0.000028	0.001939											0.488598	
Δ*dinG* Δ*polY2*	0.000006		0.002343										0.745221	
Δ*dinG* Δ*polA*	<0.000001			0.859291									0.012893	
Δ*dinG* Δ*mutSL*	<0.000001				0.000022								<0.000001	
Δ*mfd*	0.005468													
Δ*mfd* Δ*polY1*	0.617193	0.38328												0.441945
Δ*mfd* Δ*polY2*	0.100943		0.000071											0.133834
Δ*mfd* Δ*polA*	<0.000001			0.017582										0.040675
Δ*mfd* Δ*mutSL*	<0.000001				0.000034									<0.000001

[a]Purple, highly statistically significant difference (*P* <0.01); orange, statistically significant difference (*P* <0.05); black, not statistically significant difference (*P* >0.05).

Table S5. Statistical analysis of mutagenesis frequencies in the presence of 3 mM MMS (*P* values[a] for pairwise *t*-test comparisons).

	wt	Δ*polY1*	Δ*polY2*	Δ*polA*	Δ*mutSL*	Δ*recA*	Δ*lexA*	*lexA*(Ind$^-$)	Δ*disA*	Δ*rarA*	Δ*recD2*	Δ*recD2* Δ*rarA*	Δ*dinG*	Δ*mfd*
Δ*polY1*	0.000011													
Δ*polY2*	<0.000001	0.000011												
Δ*polA*	<0.000001	0.000051	0.896325											
Δ*mutSL*	<0.000001	<0.000001	<0.000001	<0.000001										
Δ*polY1* Δ*polY2*	<0.000001	<0.000001	0.000288	0.00043	<0.000001									
Δ*polY1* Δ*polA*	<0.000001	<0.000001		0.014095										
Δ*polY2* Δ*polA*	0.000009		0.434591	0.405334										
Δ*recA*	<0.000001													
Δ*recA* Δ*polY1*	<0.000001	<0.000001				0.405246								
Δ*recA* Δ*polY2*	<0.000001		0.000101			0.026513								
Δ*recA* Δ*polA*	0.159672			0.000296		0.000003								
Δ*recA* Δ*mutSL*	<0.000001				0.022228	<0.000001								
Δ*lexA*	<0.000001													
Δ*lexA* Δ*polA*	0.000006			0.003761			0.000049							
lexA(Ind$^-$)	0.290686													
lexA(Ind$^-$) Δ*polA*	<0.000001			0.063992				<0.000001						
Δ*disA*	<0.000001													
Δ*disA* Δ*polY1*	<0.000001	0.000532							0.197066					
Δ*disA* Δ*polY2*	<0.000001		0.120404						0.000007					
Δ*disA* Δ*polA*	<0.000001			0.014017					0.000003					
Δ*disA* Δ*mutSL*	<0.000001				0.000093				<0.000001					
Δ*rarA*	<0.000001													
Δ*rarA* Δ*polY1*	<0.000001	<0.000001								0.000233				
Δ*rarA* Δ*polY2*	<0.000001		0.075013							0.412128				
Δ*rarA* Δ*polA*	<0.000001			0.000055						0.000007				
Δ*rarA* Δ*mutSL*	<0.000001				0.157829					<0.000001				
Δ*recD2*	<0.000001													
Δ*recD2* Δ*polY1*	0.000161	0.000004									0.000197			
Δ*recD2* Δ*polY2*	0.000002		<0.000001								0.016165			
Δ*recD2* Δ*polA*	0.883689			0.001380							0.000002			
Δ*recD2* Δ*mutSL*	<0.000001				0.002954						<0.000001			
Δ*recD2* Δ*rarA*	0.046198									0.002618	0.003124			
Δ*recD2* Δ*rarA* Δ*polA*	0.393072			0.550416						0.006320	0.003124	<0.000001		
Δ*dinG*	<0.000001													
Δ*dinG* Δ*polY1*	<0.000001	0.000401											0.410683	
Δ*dinG* Δ*polY2*	<0.000001		0.788308										0.007682	
Δ*dinG* Δ*polA*	<0.000001			0.800894									0.028336	
Δ*dinG* Δ*mutSL*	0.000002				<0.000001								<0.000001	
Δ*mfd*	0.006101													
Δ*mfd* Δ*polY1*	0.030688	0.987823												0.414385
Δ*mfd* Δ*polY2*	0.000027		0.000004											0.036899
Δ*mfd* Δ*polA*	<0.000001			0.000597										0.220057
Δ*mfd* Δ*mutSL*	<0.000001				<0.000001									0.000054

[a]Purple, highly statistically significant difference (*P* <0.01); orange, statistically significant difference (*P* <0.05); black, not statistically significant difference (*P* >0.05).

Table S6. Spectrum of *rpoB* mutations identified in Rif^R colonies.

Strain	Condition[a]	Cluster N[b]	new[b]	Cluster I[b]									Non-*rpoB*
		G→T, V135F	C→A, L415I	C→A, Q469K	A→G, Q469R	A→T, Q469L	C→T, H482Y	A→C, H482P	A→G, H482R	C→G, H482D	C→T, S487L	C→T, H482Y + C→T, S487L	
wt	-			1	1		5	5					
	+						8		2				
Δ*polY1*	-						8		2				
	+			1	1		5		2				
Δ*polY2*	-				3		7						
	+						7		3				
Δ*polA*	-	3		2	1	1	2		1				
	+						10						
Δ*mutSL*	-				1				9				
	+				3		1		6				
Δ*disA*	-				1		8		1				
	+				3		3	1			2	1	
Δ*rarA*	-	1					3		6				1
	+						10						
Δ*recD2*	-						7		3				
	+				5		5						
Δ*dinG*	-		1		1		2		1				6
	+						10						
Δ*mfd*	-			2			4		4				
	+			1	1		4		3		1		
Δ*recA*	-						3	7					
	+						5	5					
Δ*recA* Δ*polY1*	-						8		2				
Δ*recA* Δ*polY2*	-						9			1			
Δ*recA* Δ*polA*	-				7		3						
Δ*recA* Δ*mutSL*	-				1		2		9				

[a]In the absence (-) or presence (+) of 3 mM MMS. [b]Nucleotide change and corresponding amino acid position and substitution. Orange colour, transition mutation; purple colour, transversion mutation.

REFERENCES

1. Raguse, M., Torres, R., Seco, E.M., Gándara, C., Ayora, S., Moeller, R. and Alonso, J.C. (2017) *Bacillus subtilis* DisA helps to circumvent replicative stress during spore revival. *DNA Repair (Amst)*, 59, 57-68. https://doi.org/10.1016/j.dnarep.2017.09.006
2. Carrasco, B., Torres, R., López-Sanz, M., Hernández-Tamayo, R., Graumann, P.L. and Alonso, J.C. (2025) *Bacillus subtilis* DinG 3'⟶5' Exo(ribo)nuclease: A Helpmate to Mitigate Replication Stress. *Int J Mol Sci*, 26, 9681. https://doi.org/10.3390/ijms26199681
3. Carrasco, B., Serrano, E., Martín-González, A., Moreno-Herrero, F. and Alonso, J.C. (2019) *Bacillus subtilis* MutS Modulates RecA-Mediated DNA Strand Exchange Between Divergent DNA Sequences. *Front Microbiol*, 10, 237. https://doi.org/10.3389/fmicb.2019.00237
4. Cárdenas, P.P., Carrasco, B., Defeu Soufo, C., César, C.E., Herr, K., Kaufenstein, M., Graumann, P.L. and Alonso, J.C. (2012) RecX facilitates homologous recombination by modulating RecA activities. *PLoS Genet*, 8, e1003126. https://doi.org/10.1371/journal.pgen.1003126
5. Gándara, C. and Alonso, J.C. (2015) DisA and c-di-AMP act at the intersection between DNA-damage response and stress homeostasis in exponentially growing *Bacillus subtilis* cells. *DNA Repair (Amst)*, 27, 1-8. https://doi.org/10.1016/j.dnarep.2014.12.007
6. Carrasco, B., Seco, E.M., López-Sanz, M., Alonso, J.C. and Ayora, S. (2018) *Bacillus subtilis* RarA modulates replication restart. *Nucleic Acids Res*, 46, 7206-7220. https://doi.org/10.1093/nar/gky541
7. Romero, H., Torres, R., Hernández-Tamayo, R., Carrasco, B., Ayora, S., Graumann, P.L. and Alonso, J.C. (2019) *Bacillus subtilis* RarA acts at the interplay between replication and repair-by-recombination. *DNA Repair (Amst)*, 78, 27-36. https://doi.org/10.1016/j.dnarep.2019.03.010
8. Torres, R., Romero, H., Rodríguez-Cerrato, V. and Alonso, J.C. (2017) Interplay between *Bacillus subtilis* RecD2 and the RecG or RuvAB helicase in recombinational repair. *DNA Repair (Amst)*, 55, 40-46. https://doi.org/10.1016/j.dnarep.2017.05.004
9. Ayora, S., Rojo, F., Ogasawara, N., Nakai, S. and Alonso, J.C. (1996) The Mfd protein of *Bacillus subtilis* 168 is involved in both transcription-coupled DNA repair and DNA recombination. *J Mol Biol*, 256, 301-318. https://doi.org/10.1006/jmbi.1996.0087

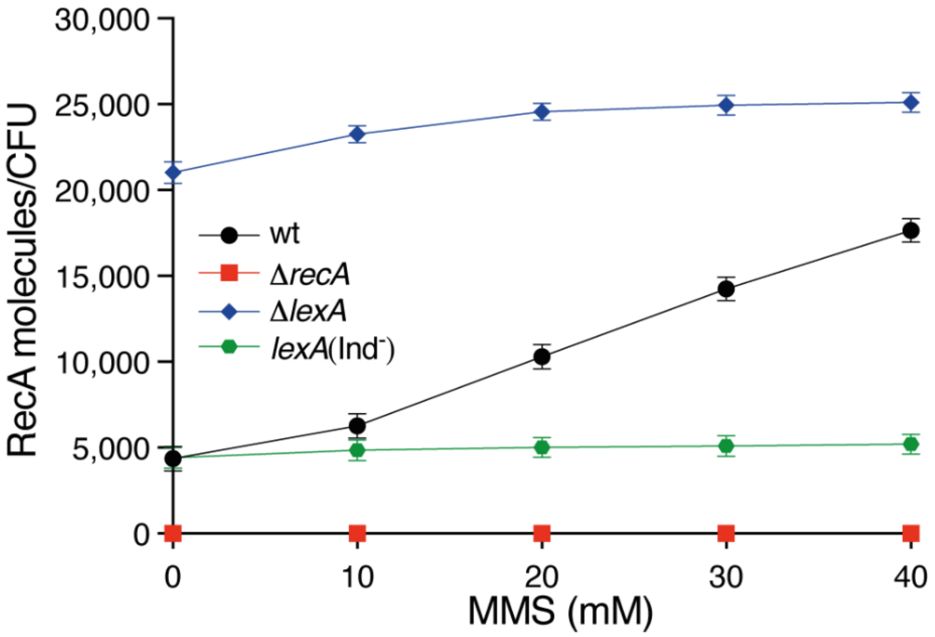

Figure S1. SOS induction is constitutive in the absence of LexA and abolished in the presence of a non-cleavable LexA variant. The indicated strains were grown to exponential phase (OD_{560}= 0.4) in LB medium at 37 ºC and treated with increasing concentrations of MMS for 30 min. Cells were lysed, and RecA levels, used as a proxy for SOS response induction, were determined by Western blotting. Data represent the mean ± SD of >3 independent experiments.

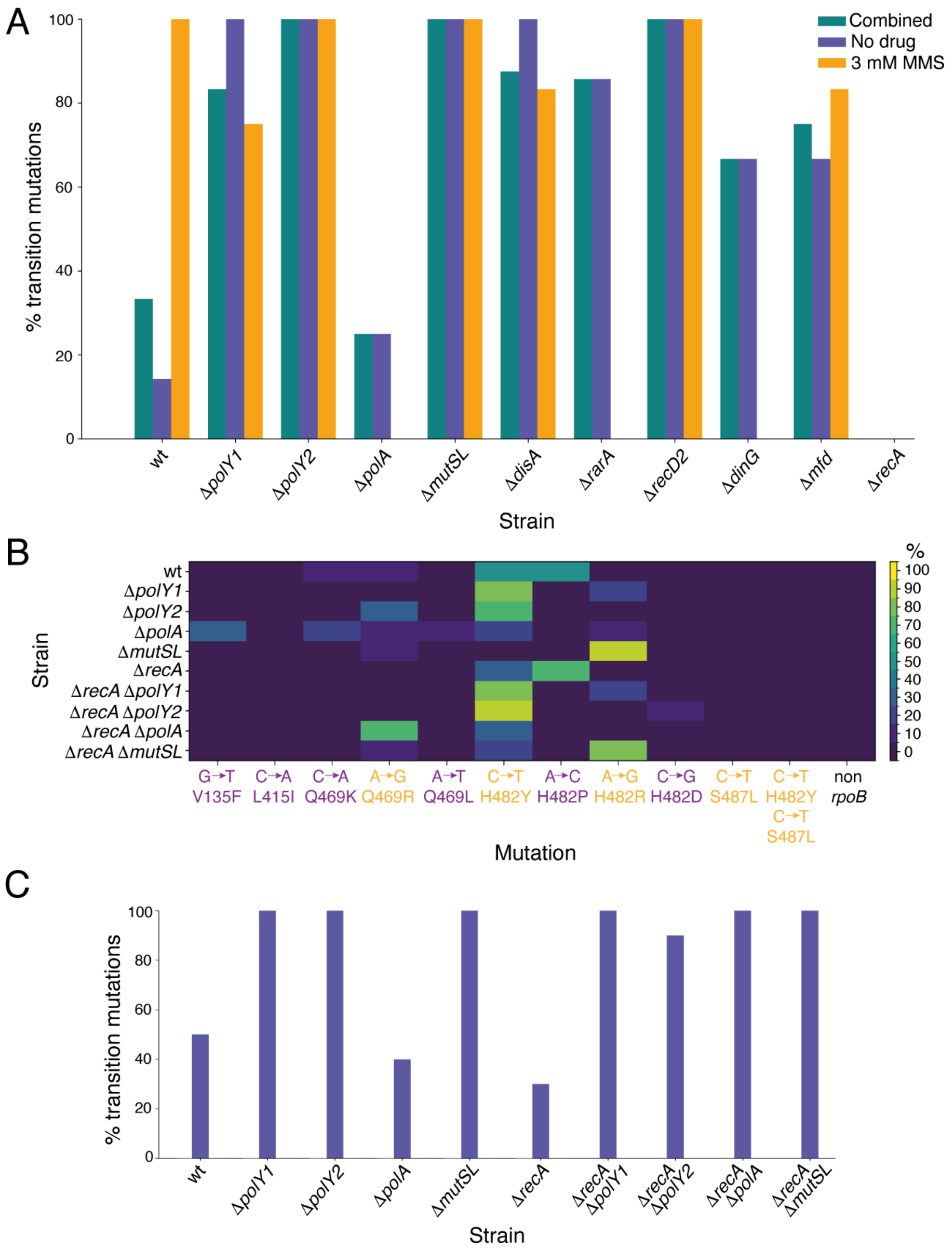

Figure S2. Spontaneous mutational landscape in TLS DNAPs or MMR mutants in the absence of RecA. (A) Bar graph showing the proportion of transition mutations in each strain, combining both conditions (green), or separately in the absence (blue) or presence (orange) of MMS, excluding the H482Y mutation. (B) Heat map showing the proportion of each mutation detected in each strain in the absence of MMS. Orange colour, transition mutation; purple colour, transversion mutation. (C) Bar graph showing the proportion of transition mutations in each strain in the absence of MMS.
