# Supplementary material for: Genetic dissection of DNA damage tolerance in Bacillus subtilis: RecA and recombination functions regulate translesion synthesis
Source: Nucleic Acids Res. 2026 Jul 6;54(13):gkag673. doi: 10.1093/nar/gkag673 (PMC13335488; doi:10.1093/nar/gkag673)

Index

page nº	PolA	PolY1	PolY2	MutL	MutS	DnaN	RecA	DisA	Mfd	RecD2	RarA	DinG	- control
PolA	2	3	4	5	6	7	8	9	10	11	12	13	80
PolY1	3	14	15	16	17	18	19	20	21	22	23	24	80
PolY2	4	15	25	26	27	28	29	30	31	32	33	34	81
MutL	5	16	26	35	36	37	38	39	40	41	42	43	81
MutS	6	17	27	36	44	45	46	47	48	49	50	51	82
DnaN	7	18	28	37	45	52	53	54	55	56	57	58	82
RecA	8	19	29	38	46	53	59	60	61	62	63	64	83
DisA	9	20	30	39	47	54	60	65	66	67	68	69	83
Mfd	10	21	31	40	48	55	61	66	70	71	72	73	84
RecD2	11	22	32	41	49	56	62	67	71	74	75	76	84
RarA	12	23	33	42	50	57	63	68	72	75	77	78	85
DinG	13	24	34	43	51	58	64	69	73	76	78	79	85
- control	80	80	81	81	82	82	83	83	84	84	85	85	86

PolA – PolA

1. pUT18-*polA* – pKT25-*polA*
2. pU18C-*polA* – pKT25-*polA*
3. pUT18-*polA* – pKNT25-*polA*
4. pU18C-*polA* – pKNT25-*polA*

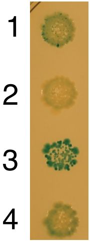

PolA – PolY1

1. pUT18-*polY1* – pKT25-*polA*
2. pUT18-*polY1* – pKNT25-*polA*
3. pKT25-*polY1* – pUT18-*polA*
4. pKNT25-*polY1* – pUT18-*polA*
5. pKT25-*polY1* – pUT18C-*polA*
6. pKNT25-*polY1* – pUT18C-*polA*

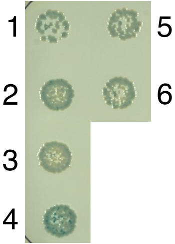

PolA – PolY2

1. pUT18-*polY2* – pKT25-*polA*
2. pU18C-*polY2* – pKT25-*polA*
3. pUT18-*polY2* – pKNT25-*polA*
4. pU18C-*polY2* – pKNT25-*polA*
5. pKT25-*polY2* – pUT18-*polA*
6. pKNT25-*polY2* – pUT18-*polA*
7. pKT25-*polY2* – pUT18C-*polA*
8. pKNT25-*polY2* – pUT18C-*polA*

1
2
3
4

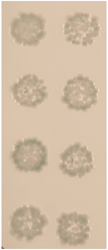

5
6
7
8

PolA – MutL

1. pUT18-*mutL* – pKT25-*polA*
2. pU18C-*mutL* – pKT25-*polA*
3. pUT18-*mutL* – pKNT25-*polA*
4. pU18C-*mutL* – pKNT25-*polA*
5. pKT25-*mutL* – pUT18-*polA*
6. pKNT25-*mutL* – pUT18-*polA*
7. pKT25-*mutL* – pUT18C-*polA*
8. pKNT25-*mutL* – pUT18C-*polA*

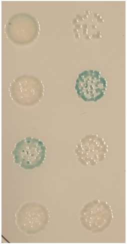

PolA – MutS

1. pUT18-*mutS* – pKT25-*polA*
2. pU18C-*mutS* – pKT25-*polA*
3. pUT18-*mutS* – pKNT25-*polA*
4. pU18C-*mutS* – pKNT25-*polA*
5. pKT25-*mutS* – pUT18-*polA*
6. pKNT25-*mutS* – pUT18-*polA*
7. pKT25-*mutS* – pUT18C-*polA*
8. pKNT25-*mutS* – pUT18C-*polA*

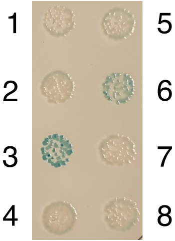

PolA – DnaN

1. pUT18-*polA* – pKT25-*dnaN*
2. pU18C-*polA* – pKT25-*dnaN*
3. pUT18-*polA* – pKNT25-*dnaN*
4. pU18C-*polA* – pKNT25-*dnaN*
5. pKT25-*polA* – pUT18-*dnaN*
6. pKNT25-*polA* – pUT18-*dnaN*
7. pKT25-*polA* – pUT18C-*dnaN*
8. pKNT25-*polA* – pUT18C-*dnaN*

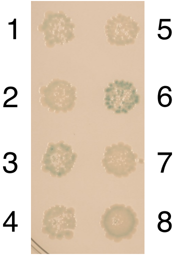

PolA – RecA

1. pUT18-*polA* – pKT25-*recA*
2. pU18C-*polA* – pKT25-*recA*
3. pUT18-*polA* – pKNT25-*recA*
4. pU18C-*polA* – pKNT25-*recA*
5. pKT25-*polA* – pUT18-*recA*
6. pKNT25-*polA* – pUT18-*recA*
7. pKT25-*polA* – pUT18C-*recA*
8. pKNT25-*polA* – pUT18C-*recA*

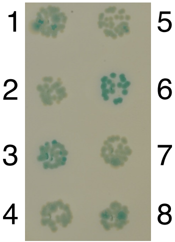

PolA – DisA

1. pUT18-*disA* – pKT25-*polA*
2. pU18C-*disA* – pKT25-*polA*
3. pUT18-*disA* – pKNT25-*polA*
4. pU18C-*disA* – pKNT25-*polA*
5. pKT25-*disA* – pUT18-*polA*
6. pKNT25-*disA* – pUT18-*polA*
7. pKT25-*disA* – pUT18C-*polA*
8. pKNT25-*disA* – pUT18C-*polA*

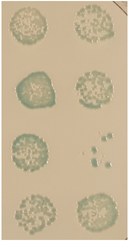

1 5
2 6
3 7
4 8

PolA – Mfd

1. pUT18-*polA* – pKT25-*mfd*
2. pU18C-*polA* – pKT25-*mfd*
3. pUT18-*polA* – pKNT25-*mfd*
4. pU18C-*polA* – pKNT25-*mfd*
5. pKT25-*polA* – pUT18-*mfd*
6. pKNT25-*polA* – pUT18-*mfd*
7. pKT25-*polA* – pUT18C-*mfd*
8. pKNT25-*polA* – pUT18C-*mfd*

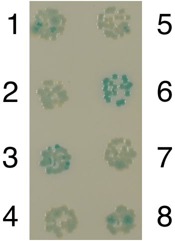

PolA – RecD2

1. pUT18-*polA* – pKT25-*recD2*
2. pU18C-*polA* – pKT25-*recD2*
3. pUT18-*polA* – pKNT25-*recD2*
4. pU18C-*polA* – pKNT25-*recD2*
5. pKT25-*polA* – pUT18-*recD2*
6. pKNT25-*polA* – pUT18-*recD2*
7. pKT25-*polA* – pUT18C-*recD2*
8. pKNT25-*polA* – pUT18C-*recD2*

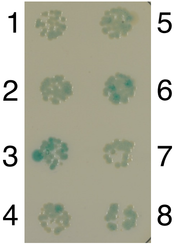

PolA – RarA

1. pUT18-*polA* – pKT25-*rarA*
2. pU18C-*polA* – pKT25-*rarA*
3. pUT18-*polA* – pKNT25-*rarA*
4. pU18C-*polA* – pKNT25-*rarA*
5. pKT25-*polA* – pUT18-*rarA*
6. pKNT25-*polA* – pUT18-*rarA*
7. pKT25-*polA* – pUT18C-*rarA*
8. pKNT25-*polA* – pUT18C-*rarA*

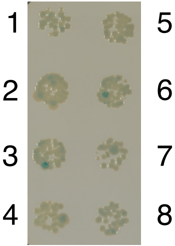

PolA – DinG

1. pUT18-*polA* – pKT25-*dinG*
2. pU18C-*polA* – pKT25-*dinG*
3. pUT18-*polA* – pKNT25-*dinG*
4. pU18C-*polA* – pKNT25-*dinG*
5. pKT25-*polA* – pUT18-*dinG*
6. pKNT25-*polA* – pUT18-*dinG*
7. pKT25-*polA* – pUT18C-*dinG*
8. pKNT25-*polA* – pUT18C-*dinG*

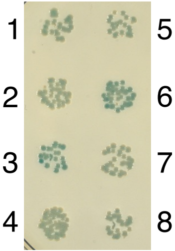

PolY1 – PolY1

1. pUT18-*polY1* – pKT25-*polY1*
2. pUT18-*polY1* – pKNT25-*polY1*

1

2

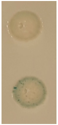

PolY1 – PolY2

1. pUT18-*polY2* – pKT25-*polY1*
2. pU18C-*polY2* – pKT25-*polY1*
3. pUT18-*polY2* – pKNT25-*polY1*
4. pU18C-*polY2* – pKNT25-*polY1*
5. pKT25-*polY2* – pUT18-*polY1*
6. pKNT25-*polY2* – pUT18-*polY1*

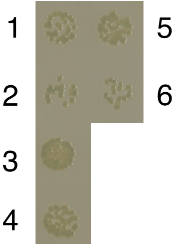

PolY1 – MutL

1. pUT18-*mutL* – pKT25-*polY1*
2. pU18C-*mutL* – pKT25-*polY1*
3. pUT18-*mutL* – pKNT25-*polY1*
4. pU18C-*mutL* – pKNT25-*polY1*
5. pKT25-*mutL* – pUT18-*polY1*
6. pKNT25-*mutL* – pUT18-*polY1*

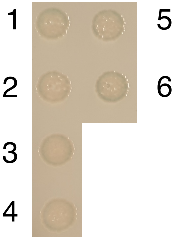

PolY1 – MutS

1. pUT18-*mutS* – pKT25-*polY1*
2. pU18C-*mutS* – pKT25-*polY1*
3. pUT18-*mutS* – pKNT25-*polY1*
4. pU18C-*mutS* – pKNT25-*polY1*
5. pKT25-*mutS* – pUT18-*polY1*
6. pKNT25-*mutS* – pUT18-*polY1*

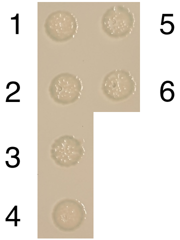

PolY1 – DnaN

1. pUT18-*polY1* – pKT25-*dnaN*
2. pUT18-*polY1* – pKNT25-*dnaN*
3. pKT25-*polY1* – pUT18-*dnaN*
4. pKNT25-*polY1* – pUT18-*dnaN*
5. pKT25-*polY1* – pUT18C-*dnaN*
6. pKNT25-*polY1* – pUT18C-*dnaN*

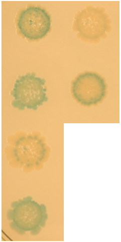

PolY1 – RecA

1. pUT18-*polY1* – pKT25-*recA*
2. pUT18-*polY1* – pKNT25-*recA*
3. pKT25-*polY1* – pUT18-*recA*
4. pKNT25-*polY1* – pUT18-*recA*
5. pKT25-*polY1* – pUT18C-*recA*
6. pKNT25-*polY1* – pUT18C-*recA*

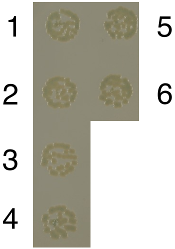

PolY1 – DisA

1. pUT18-*polY1* – pKT25-*disA*
2. pUT18-*polY1* – pKNT25-*disA*
3. pKT25-*polY1* – pUT18-*disA*
4. pKNT25-*polY1* – pUT18-*disA*
5. pKT25-*polY1* – pUT18C-*disA*
6. pKNT25-*polY1* – pUT18C-*disA*

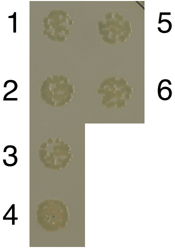

PolY1 – Mfd

1. pUT18-*polY1* – pKT25-*mfd*
2. pUT18-*polY1* – pKNT25-*mfd*
3. pKT25-*polY1* – pUT18-*mfd*
4. pKNT25-*polY1* – pUT18-*mfd*
5. pKT25-*polY1* – pUT18C-*mfd*
6. pKNT25-*polY1* – pUT18C-*mfd*

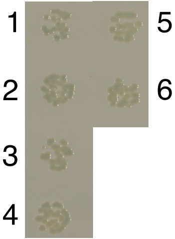

PolY1 – RecD2

1. pUT18-*polY1* – pKT25-*recD2*
2. pUT18-*polY1* – pKNT25-*recD2*
3. pKT25-*polY1* – pUT18-*recD2*
4. pKNT25-*polY1* – pUT18-*recD2*
5. pKT25-*polY1* – pUT18C-*recD2*
6. pKNT25-*polY1* – pUT18C-*recD2*

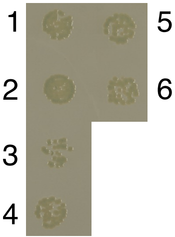

PolY1 – RarA

1. pUT18-*polY1* – pKT25-*rarA*
2. pUT18-*polY1* – pKNT25-*rarA*
3. pKT25-*polY1* – pUT18-*rarA*
4. pKNT25-*polY1* – pUT18-*rarA*
5. pKT25-*polY1* – pUT18C-*rarA*
6. pKNT25-*polY1* – pUT18C-*rarA*

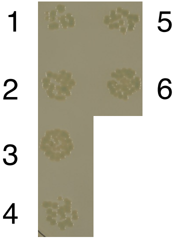

PolY1 – DinG

1. pUT18-*polY1* – pKT25-*dinG*
2. pUT18-*polY1* – pKNT25-*dinG*
3. pKT25-*polY1* – pUT18-*dinG*
4. pKNT25-*polY1* – pUT18-*dinG*
5. pKT25-*polY1* – pUT18C-*dinG*
6. pKNT25-*polY1* – pUT18C-*dinG*

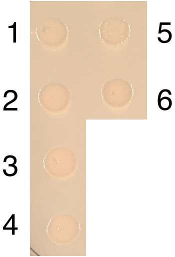

PolY2 – PolY2

1. pUT18-*polY2* – pKT25-*polY2*
2. pU18C-*polY2* – pKT25-*polY2*
3. pUT18-*polY2* – pKNT25-*polY2*
4. pU18C-*polY2* – pKNT25-*polY2*

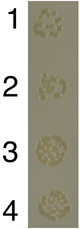

PolY2 – MutL

1. pUT18-*mutL* – pKT25-*polY2*
2. pU18C-*mutL* – pKT25-*polY2*
3. pUT18-*mutL* – pKNT25-*polY2*
4. pU18C-*mutL* – pKNT25-*polY2*
5. pKT25-*mutL* – pUT18-*polY2*
6. pKNT25-*mutL* – pUT18-*polY2*
7. pKT25-*mutL* – pUT18C-*polY2*
8. pKNT25-*mutL* – pUT18C-*polY2*

1 5
2 6
3 7
4 8

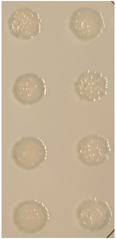

PolY2 – MutS

1. pUT18-*mutS* – pKT25-*polY2*
2. pU18C-*mutS* – pKT25-*polY2*
3. pUT18-*mutS* – pKNT25-*polY2*
4. pU18C-*mutS* – pKNT25-*polY2*
5. pKT25-*mutS* – pUT18-*polY2*
6. pKNT25-*mutS* – pUT18-*polY2*
7. pKT25-*mutS* – pUT18C-*polY2*
8. pKNT25-*mutS* – pUT18C-*polY2*

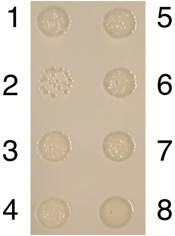

PolY2 – DnaN

1. pUT18-*polY2* – pKT25-*dnaN*
2. pU18C-*polY2* – pKT25-*dnaN*
3. pUT18-*polY2* – pKNT25-*dnaN*
4. pU18C-*polY2* – pKNT25-*dnaN*
5. pKT25-*polY2* – pUT18-*dnaN*
6. pKNT25-*polY2* – pUT18-*dnaN*
7. pKT25-*polY2* – pUT18C-*dnaN*
8. pKNT25-*polY2* – pUT18C-*dnaN*

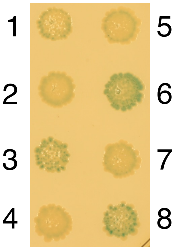

PolY2 – RecA

1. pUT18-*polY2* – pKT25-*recA*
2. pU18C-*polY2* – pKT25-*recA*
3. pUT18-*polY2* – pKNT25-*recA*
4. pU18C-*polY2* – pKNT25-*recA*
5. pKT25-*polY2* – pUT18-*recA*
6. pKNT25-*polY2* – pUT18-*recA*
7. pKT25-*polY2* – pUT18C-*recA*
8. pKNT25-*polY2* – pUT18C-*recA*

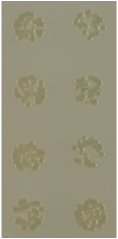

1 5
2 6
3 7
4 8

PolY2 – DisA

1. pUT18-*polY2* – pKT25-*disA*
2. pU18C-*polY2* – pKT25-*disA*
3. pUT18-*polY2* – pKNT25-*disA*
4. pU18C-*polY2* – pKNT25-*disA*
5. pKT25-*polY2* – pUT18-*disA*
6. pKNT25-*polY2* – pUT18-*disA*
7. pKT25-*polY2* – pUT18C-*disA*
8. pKNT25-*polY2* – pUT18C-*disA*

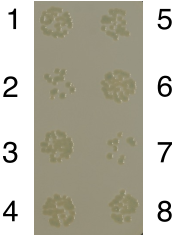

PolY2 – Mfd

1. pUT18-*polY2* – pKT25-*mfd*
2. pU18C-*polY2* – pKT25-*mfd*
3. pUT18-*polY2* – pKNT25-*mfd*
4. pU18C-*polY2* – pKNT25-*mfd*
5. pKT25-*polY2* – pUT18-*mfd*
6. pKNT25-*polY2* – pUT18-*mfd*
7. pKT25-*polY2* – pUT18C-*mfd*
8. pKNT25-*polY2* – pUT18C-*mfd*

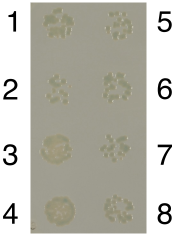

PolY2 – RecD2

1. pUT18-*polY2* – pKT25-*recD2*
2. pU18C-*polY2* – pKT25-*recD2*
3. pUT18-*polY2* – pKNT25-*recD2*
4. pU18C-*polY2* – pKNT25-*recD2*
5. pKT25-*polY2* – pUT18-*recD2*
6. pKNT25-*polY2* – pUT18-*recD2*
7. pKT25-*polY2* – pUT18C-*recD2*
8. pKNT25-*polY2* – pUT18C-*recD2*

1 5
2 6
3 7
4 8

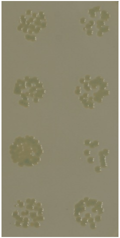

PolY2 – RarA

1. pUT18-*polY2* – pKT25-*rarA*
2. pU18C-*polY2* – pKT25-*rarA*
3. pUT18-*polY2* – pKNT25-*rarA*
4. pU18C-*polY2* – pKNT25-*rarA*
5. pKT25-*polY2* – pUT18-*rarA*
6. pKNT25-*polY2* – pUT18-*rarA*
7. pKT25-*polY2* – pUT18C-*rarA*
8. pKNT25-*polY2* – pUT18C-*rarA*

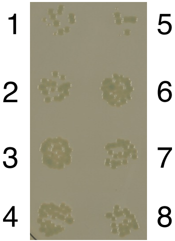

PolY2 – DinG

1. pUT18-*polY2* – pKT25-*dinG*
2. pU18C-*polY2* – pKT25-*dinG*
3. pUT18-*polY2* – pKNT25-*dinG*
4. pU18C-*polY2* – pKNT25-*dinG*
5. pKT25-*polY2* – pUT18-*dinG*
6. pKNT25-*polY2* – pUT18-*dinG*
7. pKT25-*polY2* – pUT18C-*dinG*
8. pKNT25-*polY2* – pUT18C-*dinG*

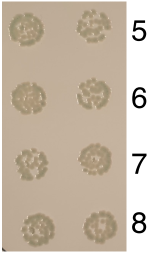

MutL – MutL

1. pUT18-*mutL* – pKT25-*mutL*
2. pU18C-*mutL* – pKT25-*mutL*
3. pUT18-*mutL* – pKNT25-*mutL*
4. pU18C-*mutL* – pKNT25-*mutL*

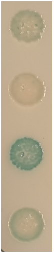

MutL – MutS

1. pUT18-*mutL* – pKT25-*mutS*
2. pU18C-*mutL* – pKT25-*mutS*
3. pUT18-*mutL* – pKNT25-*mutS*
4. pU18C-*mutL* – pKNT25-*mutS*
5. pKT25-*mutL* – pUT18-*mutS*
6. pKNT25-*mutL* – pUT18-*mutS*
7. pKT25-*mutL* – pUT18C-*mutS*
8. pKNT25-*mutL* – pUT18C-*mutS*

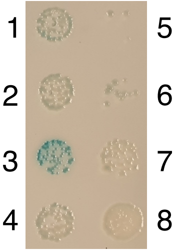

MutL – DnaN

1. pUT18-*dnaN* – pKT25-*mutL*
2. pU18C-*dnaN* – pKT25-*mutL*
3. pUT18-*dnaN* – pKNT25-*mutL*
4. pU18C-*dnaN* – pKNT25-*mutL*
5. pKT25-*dnaN* – pUT18-*mutL*
6. pKNT25-*dnaN* – pUT18-*mutL*
7. pKT25-*dnaN* – pUT18C-*mutL*
8. pKNT25-*dnaN* – pUT18C-*mutL*

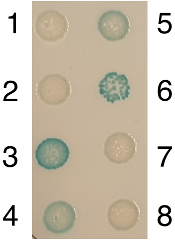

MutL – RecA

1. pUT18-*recA* – pKT25-*mutL*
2. pU18C-*recA* – pKT25-*mutL*
3. pUT18-*recA* – pKNT25-*mutL*
4. pU18C-*recA* – pKNT25-*mutL*
5. pKT25-*recA* – pUT18-*mutL*
6. pKNT25-*recA* – pUT18-*mutL*
7. pKT25-*recA* – pUT18C-*mutL*
8. pKNT25-*recA* – pUT18C-*mutL*

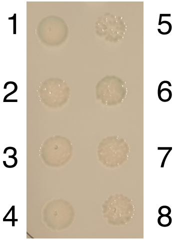

MutL – DisA

1. pUT18-*mutL* – pKT25-*disA*
2. pU18C-*mutL* – pKT25-*disA*
3. pUT18-*mutL* – pKNT25-*disA*
4. pU18C-*mutL* – pKNT25-*disA*
5. pKT25-*mutL* – pUT18-*disA*
6. pKNT25-*mutL* – pUT18-*disA*
7. pKT25-*mutL* – pUT18C-*disA*
8. pKNT25-*mutL* – pUT18C-*disA*

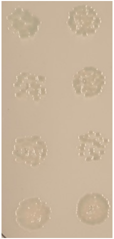

MutL – Mfd

1. pUT18-*mutL* – pKT25-*mfd*
2. pU18C-*mutL* – pKT25-*mfd*
3. pUT18-*mutL* – pKNT25-*mfd*
4. pU18C-*mutL* – pKNT25-*mfd*
5. pKT25-*mutL* – pUT18-*mfd*
6. pKNT25-*mutL* – pUT18-*mfd*
7. pKT25-*mutL* – pUT18C-*mfd*
8. pKNT25-*mutL* – pUT18C-*mfd*

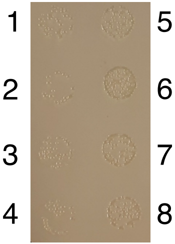

MutL – RecD2

1. pUT18-*mutL* – pKT25-*recD2*
2. pU18C-*mutL* – pKT25-*recD2*
3. pUT18-*mutL* – pKNT25-*recD2*
4. pU18C-*mutL* – pKNT25-*recD2*
5. pKT25-*mutL* – pUT18-*recD2*
6. pKNT25-*mutL* – pUT18-*recD2*
7. pKT25-*mutL* – pUT18C-*recD2*
8. pKNT25-*mutL* – pUT18C-*recD2*

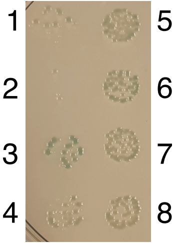

MutL – RarA

1. pUT18-*mutL* – pKT25-*rarA*
2. pU18C-*mutL* – pKT25-*rarA*
3. pUT18-*mutL* – pKNT25-*rarA*
4. pU18C-*mutL* – pKNT25-*rarA*
5. pKT25-*mutL* – pUT18-*rarA*
6. pKNT25-*mutL* – pUT18-*rarA*
7. pKT25-*mutL* – pUT18C-*rarA*
8. pKNT25-*mutL* – pUT18C-*rarA*

1 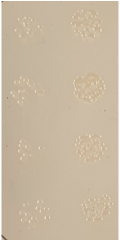 5

2 6

3 7

4 8

MutL – DinG

1. pUT18-*mutL* – pKT25-*dinG*
2. pU18C-*mutL* – pKT25-*dinG*
3. pUT18-*mutL* – pKNT25-*dinG*
4. pU18C-*mutL* – pKNT25-*dinG*
5. pKT25-*mutL* – pUT18-*dinG*
6. pKNT25-*mutL* – pUT18-*dinG*
7. pKT25-*mutL* – pUT18C-*dinG*
8. pKNT25-*mutL* – pUT18C-*dinG*

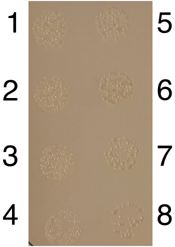

MutS – MutS

1. pUT18-*mutS* – pKT25-*mutS*
2. pU18C-*mutS* – pKT25-*mutS*
3. pUT18-*mutS* – pKNT25-*mutS*
4. pU18C-*mutS* – pKNT25-*mutS*

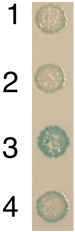

MutS – DnaN

1. pUT18-*mutS* – pKT25-*dnaN*
2. pU18C-*mutS* – pKT25-*dnaN*
3. pUT18-*mutS* – pKNT25-*dnaN*
4. pU18C-*mutS* – pKNT25-*dnaN*
5. pKT25-*mutS* – pUT18-*dnaN*
6. pKNT25-*mutS* – pUT18-*dnaN*
7. pKT25-*mutS* – pUT18C-*dnaN*
8. pKNT25-*mutS* – pUT18C-*dnaN*

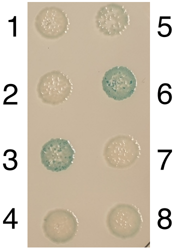

MutS – RecA

1. pUT18-*recA* – pKT25-*mutS*
2. pU18C-*recA* – pKT25-*mutS*
3. pUT18-*recA* – pKNT25-*mutS*
4. pU18C-*recA* – pKNT25-*mutS*
5. pKT25-*recA* – pUT18-*mutS*
6. pKNT25-*recA* – pUT18-*mutS*
7. pKT25-*recA* – pUT18C-*mutS*
8. pKNT25-*recA* – pUT18C-*mutS*

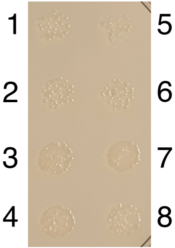

MutS – DisA

1. pUT18-*mutS* – pKT25-*disA*
2. pU18C-*mutS* – pKT25-*disA*
3. pUT18-*mutS* – pKNT25-*disA*
4. pU18C-*mutS* – pKNT25-*disA*
5. pKT25-*mutS* – pUT18-*disA*
6. pKNT25-*mutS* – pUT18-*disA*
7. pKT25-*mutS* – pUT18C-*disA*
8. pKNT25-*mutS* – pUT18C-*disA*

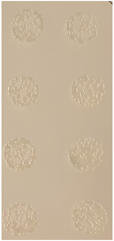

MutS – Mfd

1. pUT18-*mutS* – pKT25-*mfd*
2. pU18C-*mutS* – pKT25-*mfd*
3. pUT18-*mutS* – pKNT25-*mfd*
4. pU18C-*mutS* – pKNT25-*mfd*
5. pKT25-*mutS* – pUT18-*mfd*
6. pKNT25-*mutS* – pUT18-*mfd*
7. pKT25-*mutS* – pUT18C-*mfd*
8. pKNT25-*mutS* – pUT18C-*mfd*

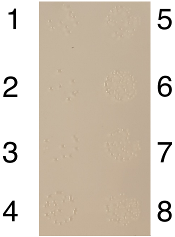

MutS – RecD2

1. pUT18-*mutS* – pKT25-*recD2*
2. pU18C-*mutS* – pKT25-*recD2*
3. pUT18-*mutS* – pKNT25-*recD2*
4. pU18C-*mutS* – pKNT25-*recD2*
5. pKT25-*mutS* – pUT18-*recD2*
6. pKNT25-*mutS* – pUT18-*recD2*
7. pKT25-*mutS* – pUT18C-*recD2*
8. pKNT25-*mutS* – pUT18C-*recD2*

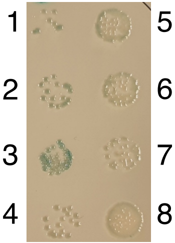

MutS – RarA

1. pUT18-*mutS* – pKT25-*rarA*
2. pU18C-*mutS* – pKT25-*rarA*
3. pUT18-*mutS* – pKNT25-*rarA*
4. pU18C-*mutS* – pKNT25-*rarA*
5. pKT25-*mutS* – pUT18-*rarA*
6. pKNT25-*mutS* – pUT18-*rarA*
7. pKT25-*mutS* – pUT18C-*rarA*
8. pKNT25-*mutS* – pUT18C-*rarA*

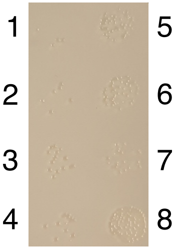

MutS – DinG

1. pUT18-*mutS* – pKT25-*dinG*
2. pU18C-*mutS* – pKT25-*dinG*
3. pUT18-*mutS* – pKNT25-*dinG*
4. pU18C-*mutS* – pKNT25-*dinG*
5. pKT25-*mutS* – pUT18-*dinG*
6. pKNT25-*mutS* – pUT18-*dinG*
7. pKT25-*mutS* – pUT18C-*dinG*
8. pKNT25-*mutS* – pUT18C-*dinG*

1 2 3 4

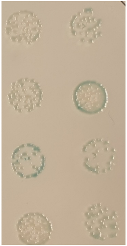

5 6 7 8

DnaN – DnaN

1. pUT18-*dnaN* – pKT25-*dnaN*
2. pU18C-*dnaN* – pKT25-*dnaN*
3. pUT18-*dnaN* – pKNT25-*dnaN*
4. pU18C-*dnaN* – pKNT25-*dnaN*

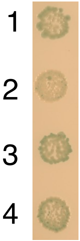

DnaN – RecA

1. pUT18-*recA* – pKT25-*dnaN*
2. pU18C-*recA* – pKT25-*dnaN*
3. pUT18-*recA* – pKNT25-*dnaN*
4. pU18C-*recA* – pKNT25-*dnaN*
5. pKT25-*recA* – pUT18-*dnaN*
6. pKNT25-*recA* – pUT18-*dnaN*
7. pKT25-*recA* – pUT18C-*dnaN*
8. pKNT25-*recA* – pUT18C-*dnaN*

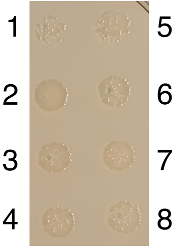

DnaN – DisA

1. pUT18-*dnaN* – pKT25-*disA*
2. pU18C-*dnaN* – pKT25-*disA*
3. pUT18-*dnaN* – pKNT25-*disA*
4. pU18C-*dnaN* – pKNT25-*disA*
5. pKT25-*dnaN* – pUT18-*disA*
6. pKNT25-*dnaN* – pUT18-*disA*
7. pKT25-*dnaN* – pUT18C-*disA*
8. pKNT25-*dnaN* – pUT18C-*disA*

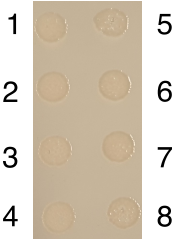

DnaN – Mfd

1. pUT18-*dnaN* – pKT25-*mfd*
2. pU18C-*dnaN* – pKT25-*mfd*
3. pUT18-*dnaN* – pKNT25-*mfd*
4. pU18C-*dnaN* – pKNT25-*mfd*
5. pKT25-*dnaN* – pUT18-*mfd*
6. pKNT25-*dnaN* – pUT18-*mfd*
7. pKT25-*dnaN* – pUT18C-*mfd*
8. pKNT25-*dnaN* – pUT18C-*mfd*

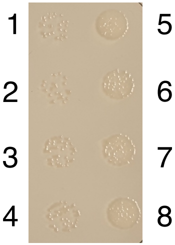

DnaN – RecD2

1. pUT18-*recD2* – pKT25-*dnaN*
2. pU18C-*recD2* – pKT25-*dnaN*
3. pUT18-*recD2* – pKNT25-*dnaN*
4. pU18C-*recD2* – pKNT25-*dnaN*
5. pKT25-*recD2* – pUT18-*dnaN*
6. pKNT25-*recD2* – pUT18-*dnaN*
7. pKT25-*recD2* – pUT18C-*dnaN*
8. pKNT25-*recD2* – pUT18C-*dnaN*

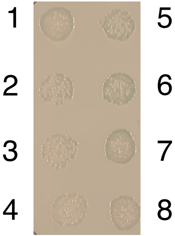

DnaN – RarA

1. pUT18-*rarA* – pKT25-*dnaN*
2. pU18C-*rarA* – pKT25-*dnaN*
3. pUT18-*rarA* – pKNT25-*dnaN*
4. pU18C-*rarA* – pKNT25-*dnaN*
5. pKT25-*rarA* – pUT18-*dnaN*
6. pKNT25-*rarA* – pUT18-*dnaN*
7. pKT25-*rarA* – pUT18C-*dnaN*
8. pKNT25-*rarA* – pUT18C-*dnaN*

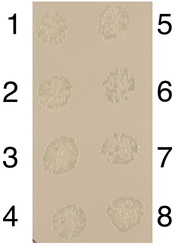

DnaN – DinG

1. pUT18-*dnaN* – pKT25-*dinG*
2. pU18C-*dnaN* – pKT25-*dinG*
3. pUT18-*dnaN* – pKNT25-*dinG*
4. pU18C-*dnaN* – pKNT25-*dinG*
5. pKT25-*dnaN* – pUT18-*dinG*
6. pKNT25-*dnaN* – pUT18-*dinG*
7. pKT25-*dnaN* – pUT18C-*dinG*
8. pKNT25-*dnaN* – pUT18C-*dinG*

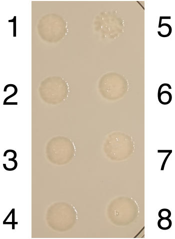

RecA – RecA

1. pUT18-*recA* – pKT25-*recA*
2. pU18C-*recA* – pKT25-*recA*
3. pUT18-*recA* – pKNT25-*recA*
4. pU18C-*recA* – pKNT25-*recA*

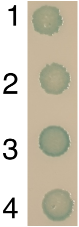

RecA – DisA

1. pUT18-*disA* – pKT25-*recA*
2. pU18C-*disA* – pKT25-*recA*
3. pUT18-*disA* – pKNT25-*recA*
4. pU18C-*disA* – pKNT25-*recA*
5. pKT25-*disA* – pUT18-*recA*
6. pKNT25-*disA* – pUT18-*recA*
7. pKT25-*disA* – pUT18C-*recA*
8. pKNT25-*disA* – pUT18C-*recA*

1 5
2 6
3 7
4 8

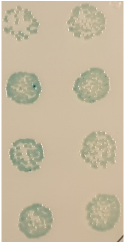

RecA – Mfd

1. pUT18-*recA* – pKT25-*mfd*
2. pU18C-*recA* – pKT25-*mfd*
3. pUT18-*recA* – pKNT25-*mfd*
4. pU18C-*recA* – pKNT25-*mfd*
5. pKT25-*recA* – pUT18-*mfd*
6. pKNT25-*recA* – pUT18-*mfd*
7. pKT25-*recA* – pUT18C-*mfd*
8. pKNT25-*recA* – pUT18C-*mfd*

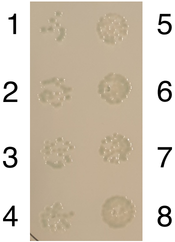

RecA – RecD2

1. pUT18-*recA* – pKT25-*recD2*
2. pU18C-*recA* – pKT25-*recD2*
3. pUT18-*recA* – pKNT25-*recD2*
4. pU18C-*recA* – pKNT25-*recD2*
5. pKT25-*recA* – pUT18-*recD2*
6. pKNT25-*recA* – pUT18-*recD2*
7. pKT25-*recA* – pUT18C-*recD2*
8. pKNT25-*recA* – pUT18C-*recD2*

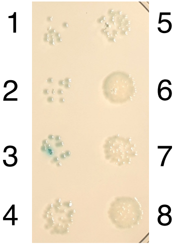

RecA – RarA

1. pUT18-*recA* – pKT25-*rarA*
2. pU18C-*recA* – pKT25-*rarA*
3. pUT18-*recA* – pKNT25-*rarA*
4. pU18C-*recA* – pKNT25-*rarA*
5. pKT25-*recA* – pUT18-*rarA*
6. pKNT25-*recA* – pUT18-*rarA*
7. pKT25-*recA* – pUT18C-*rarA*
8. pKNT25-*recA* – pUT18C-*rarA*

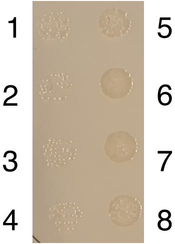

RecA – DinG

1. pUT18-*dinG* – pKT25-*recA*
2. pU18C-*dinG* – pKT25-*recA*
3. pUT18-*dinG* – pKNT25-*recA*
4. pU18C-*dinG* – pKNT25-*recA*
5. pKT25-*dinG* – pUT18-*recA*
6. pKNT25-*dinG* – pUT18-*recA*
7. pKT25-*dinG* – pUT18C-*recA*
8. pKNT25-*dinG* – pUT18C-*recA*

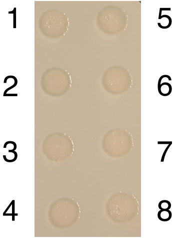

DisA – DisA

1. pUT18-*disA* – pKT25-*disA*
2. pU18C-*disA* – pKT25-*disA*
3. pUT18-*disA* – pKNT25-*disA*
4. pU18C-*disA* – pKNT25-*disA*

1
2
3
4

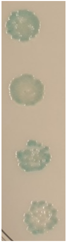

DisA – Mfd

1. pUT18-*disA* – pKT25-*mfd*
2. pU18C-*disA* – pKT25-*mfd*
3. pUT18-*disA* – pKNT25-*mfd*
4. pU18C-*disA* – pKNT25-*mfd*
5. pKT25-*disA* – pUT18-*mfd*
6. pKNT25-*disA* – pUT18-*mfd*
7. pKT25-*disA* – pUT18C-*mfd*
8. pKNT25-*disA* – pUT18C-*mfd*

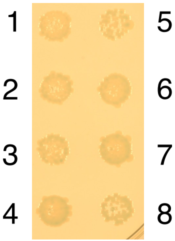

DisA – RecD2

1. pUT18-*disA* – pKT25-*recD2*
2. pU18C-*disA* – pKT25-*recD2*
3. pUT18-*disA* – pKNT25-*recD2*
4. pU18C-*disA* – pKNT25-*recD2*
5. pKT25-*disA* – pUT18-*recD2*
6. pKNT25-*disA* – pUT18-*recD2*
7. pKT25-*disA* – pUT18C-*recD2*
8. pKNT25-*disA* – pUT18C-*recD2*

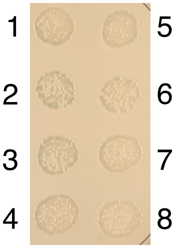

DisA – RarA

1. pUT18-*disA* – pKT25-*rarA*
2. pU18C-*disA* – pKT25-*rarA*
3. pUT18-*disA* – pKNT25-*rarA*
4. pU18C-*disA* – pKNT25-*rarA*
5. pKT25-*disA* – pUT18-*rarA*
6. pKNT25-*disA* – pUT18-*rarA*
7. pKT25-*disA* – pUT18C-*rarA*
8. pKNT25-*disA* – pUT18C-*rarA*

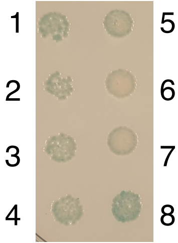

DisA – DinG

1. pUT18-*disA* – pKT25-*dinG*
2. pU18C-*disA* – pKT25-*dinG*
3. pUT18-*disA* – pKNT25-*dinG*
4. pU18C-*disA* – pKNT25-*dinG*
5. pKT25-*disA* – pUT18-*dinG*
6. pKNT25-*disA* – pUT18-*dinG*
7. pKT25-*disA* – pUT18C-*dinG*
8. pKNT25-*disA* – pUT18C-*dinG*

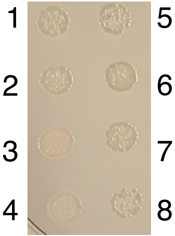

Mfd – Mfd

1. pUT18-*mfd* – pKT25-*mfd*
2. pU18C-*mfd* – pKT25-*mfd*
3. pUT18-*mfd* – pKNT25-*mfd*
4. pU18C-*mfd* – pKNT25-*mfd*

1
2
3
4

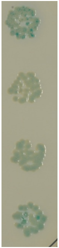

Mfd – RecD2

1. pUT18-*mfd* – pKT25-*recD2*
2. pU18C-*mfd* – pKT25-*recD2*
3. pUT18-*mfd* – pKNT25-*recD2*
4. pU18C-*mfd* – pKNT25-*recD2*
5. pKT25-*mfd* – pUT18-*recD2*
6. pKNT25-*mfd* – pUT18-*recD2*
7. pKT25-*mfd* – pUT18C-*recD2*
8. pKNT25-*mfd* – pUT18C-*recD2*

1 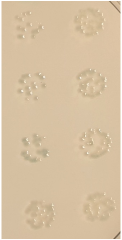 5

2 6

3 7

4 8

Mfd – RarA

1. pUT18-*mfd* – pKT25-*rarA*
2. pU18C-*mfd* – pKT25-*rarA*
3. pUT18-*mfd* – pKNT25-*rarA*
4. pU18C-*mfd* – pKNT25-*rarA*
5. pKT25-*mfd* – pUT18-*rarA*
6. pKNT25-*mfd* – pUT18-*rarA*
7. pKT25-*mfd* – pUT18C-*rarA*
8. pKNT25-*mfd* – pUT18C-*rarA*

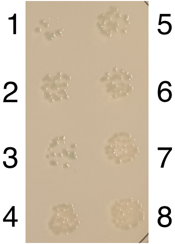

Mfd – DinG

1. pUT18-*mfd* – pKT25-*dinG*
2. pU18C-*mfd* – pKT25-*dinG*
3. pUT18-*mfd* – pKNT25-*dinG*
4. pU18C-*mfd* – pKNT25-*dinG*
5. pKT25-*mfd* – pUT18-*dinG*
6. pKNT25-*mfd* – pUT18-*dinG*
7. pKT25-*mfd* – pUT18C-*dinG*
8. pKNT25-*mfd* – pUT18C-*dinG*

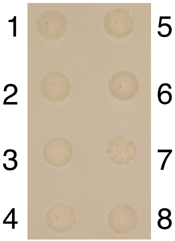

RecD2 – RecD2

1. pUT18-*recD2* – pKT25-*recD2*
2. pU18C-*recD2* – pKT25-*recD2*
3. pUT18-*recD2* – pKNT25-*recD2*
4. pU18C-*recD2* – pKNT25-*recD2*

1
2
3
4

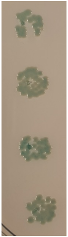

RecD2 – RarA

1. pUT18-*recD2* – pKT25-*rarA*
2. pU18C-*recD2* – pKT25-*rarA*
3. pUT18-*recD2* – pKNT25-*rarA*
4. pU18C-*recD2* – pKNT25-*rarA*
5. pKT25-*recD2* – pUT18-*rarA*
6. pKNT25-*recD2* – pUT18-*rarA*
7. pKT25-*recD2* – pUT18C-*rarA*
8. pKNT25-*recD2* – pUT18C-*rarA*

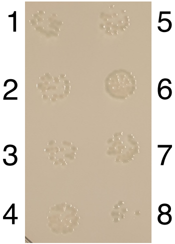

RecD2 – DinG

1. pUT18-*recD2* – pKT25-*dinG*
2. pU18C-*recD2* – pKT25-*dinG*
3. pUT18-*recD2* – pKNT25-*dinG*
4. pU18C-*recD2* – pKNT25-*dinG*
5. pKT25-*recD2* – pUT18-*dinG*
6. pKNT25-*recD2* – pUT18-*dinG*
7. pKT25-*recD2* – pUT18C-*dinG*
8. pKNT25-*recD2* – pUT18C-*dinG*

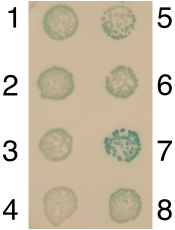

RarA – RarA

1. pUT18-*rarA* – pKT25-*rarA*
2. pU18C-*rarA* – pKT25-*rarA*
3. pUT18-*rarA* – pKNT25-*rarA*
4. pU18C-*rarA* – pKNT25-*rarA*

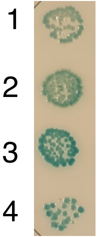

RarA – DinG

1. pUT18-*rarA* – pKT25-*dinG*
2. pU18C-*rarA* – pKT25-*dinG*
3. pUT18-*rarA* – pKNT25-*dinG*
4. pU18C-*rarA* – pKNT25-*dinG*
5. pKT25-*rarA* – pUT18-*dinG*
6. pKNT25-*rarA* – pUT18-*dinG*
7. pKT25-*rarA* – pUT18C-*dinG*
8. pKNT25-*rarA* – pUT18C-*dinG*

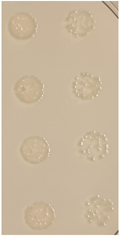

1 5
2 6
3 7
4 8

DinG – DinG

1. pUT18-*dinG* – pKT25-*dinG*
2. pU18C-*dinG* – pKT25-*dinG*
3. pUT18-*dinG* – pKNT25-*dinG*
4. pU18C-*dinG* – pKNT25-*dinG*

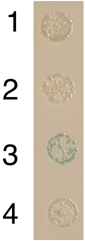

- control PolA

1. pUT18-*polA* – pKT25
2. pU18C-*polA* – pKT25
3. pKT25-*polA* – pUT18
4. pKNT25-*polA* – pUT18

1
2
3
4

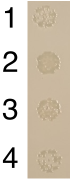

- control PolY1

5. pUT18-*polY1* – pKT25
6. pKT25-*polY1* – pUT18
7. pKNT25-*polY1* – pUT18

5
6
7

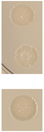

- control PolY2

1. pUT18-*polY2* – pKT25
2. pU18C-*polY2* – pKT25
3. pKT25-*polY2* – pUT18
4. pKNT25-*polY2* – pUT18

1
2
3
4

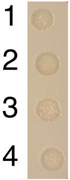

- control MutL

5. pUT18-*mutL* – pKT25
6. pU18C-*mutL* – pKT25
7. pKT25-*mutL* – pUT18
8. pKNT25-*mutL* – pUT18

5
6
7
8

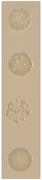

- control MutS

1. pUT18-*mutS* – pKT25
2. pU18C-*mutS* – pKT25
3. pKT25-*mutS* – pUT18
4. pKNT25-*mutS* – pUT18

1
2
3
4

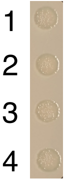

- control DnaN

5. pUT18-*dnaN* – pKT25
6. pU18C-*dnaN* – pKT25
7. pKT25-*dnaN* – pUT18
8. pKNT25-*dnaN* – pUT18

5
6
7
8

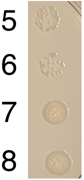

- control RecA

1. pUT18-*recA* – pKT25
2. pU18C-*recA* – pKT25
3. pKT25-*recA* – pUT18
4. pKNT25-*recA* – pUT18

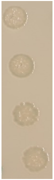

- control DisA

5. pUT18-*disA* – pKT25
6. pU18C-*disA* – pKT25
7. pKT25-*disA* – pUT18
8. pKNT25-*disA* – pUT18

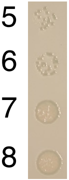

- control Mfd

1. pUT18-*mfd* – pKT25
2. pU18C-*mfd* – pKT25
3. pKT25-*mfd* – pUT18
4. pKNT25-*mfd* – pUT18

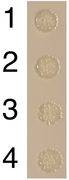

- control RecD2

5. pUT18-*recD2* – pKT25
6. pU18C-*recD2* – pKT25
7. pKT25-*recD2* – pUT18
8. pKNT25-*recD2* – pUT18

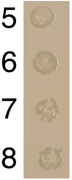

- control RarA

1. pUT18-*rarA* – pKT25
2. pU18C-*rarA* – pKT25
3. pKT25-*rarA* – pUT18
4. pKNT25-*rarA* – pUT18

1
2
3
4

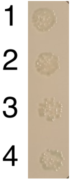

- control DinG

5. pUT18-*dinG* – pKT25
6. pU18C-*dinG* – pKT25
7. pKT25-*dinG* – pUT18
8. pKNT25-*dinG* – pUT18

5
6
7
8

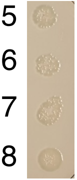

Controls

1. pUT18 – pKT25
2. pU18C-*zip* – pKT25-*zip*

1
2
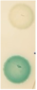

Supplement: gkag673_Supplemental_Files [file gkag673_supplemental_files.zip › Torres and Alonso Supplementary Materials File 2.pdf]
